# Deciphering the biological underpinnings behind prognostic MRI-based imaging signatures in breast cancer: a systematic review

**DOI:** 10.1186/s12967-025-07341-1

**Published:** 2025-12-17

**Authors:** Nianshi Song, Chen Gao, Xinjing Lou, Ziqing Han, Shantian Wan, Yizhen He, Zhen Fang, Yongyu An, Linyu Wu, Changyu Zhou

**Affiliations:** 1 Department of Radiology, The First Affiliated Hospital of Zhejiang Chinese Medical University (Zhejiang Provincial Hospital of Chinese Medicine), 54 Youdian Road, Hangzhou, 310006 P.R. China; 2 The First School of Clinical Medicine, Zhejiang Chinese Medical University, Hangzhou, China

**Keywords:** Radiomics, Prognosis, Breast neoplasms, Deep learning, Biological basis

## Abstract

**Objective:**

To explore the biological foundations of MRI-based prognostic imaging signatures (including radiomics and deep learning signatures) in breast cancer, and to assess the methodological quality of existing studies.

**Methods:**

This review identified studies through a comprehensive search of PubMed, Embase, Web of Science Core Collection, and the Cochrane Library through February 25, 2025. Studies on MRI-based prognostic radiomics or deep learning models with elaborated biological relevance were included. The Radiomics Quality Score (RQS), Newcastle-Ottawa Scale (NOS), and Quality Assessment of Prognostic Accuracy Studies (QUAPAS) were employed to appraise the quality of studies. Data extraction included details on study characteristics, specifics of radiomics or deep learning models, and methods leveraged for biological analysis.

**Results:**

Sixteen studies published from 2015 to 2025, comprising 61-2279 breast cancer patients, were included. Most studies employed supervised machine learning methods, with a few utilizing unsupervised machine learning methods. The underlying biological correlations mainly focused on genomic, tumor microenvironment-related, and multiomics data. The median RQS was 12.5 (range 5–17), and the mean NOS score was 7.3, reflecting limited methodological rigor. The overall risk of bias (ROB) among the studies was high, according to QUAPAS.

**Conclusion:**

The underlying biological associations of prognostic imaging signatures are mainly elucidated through genomic and transcriptomic factors. Further in-depth exploration is essential to facilitate personalized and precise treatment.

**Supplementary Information:**

The online version contains supplementary material available at 10.1186/s12967-025-07341-1.

## Introduction

Worldwide, breast cancer is the most prevalent cancer and the leading cause of cancer-related mortality among women [1]. It is estimated that new breast cancer cases will reach approximately 2.3 million in 2022, representing 11.6% of all cancer cases [2]. Admittedly, with expanding access to early detection and advancement in treatment, the breast cancer mortality rate has decreased by 44% from 1989 to 2022 [3]. Even so, breast cancer remains a great health burden for women [4]. It is highly heterogeneous, which complicates clinical decision-making and management [5, 6]. Furthermore, correlating key diagnostic indicators or histopathological findings to clinical prognosis remains an elusive challenge [7, 8]. Hence, it is necessary to develop a robust method of estimating the prognosis of breast cancer patients.

In the era of technological transformation, radiomics non-invasively assesses the heterogeneity of tumors and assists in predicting the clinical outcome with numerous high-throughput quantitative imaging features [9–11]. Particularly, many studies indicate that radiomics have great competence in decoding the intratumor heterogeneity to provide prognostic value [12–14]. Likewise, deep learning utilizes artificial neural networks to transform the raw data into a representation at a higher and more abstract level [15]. In addition to radiomics, deep learning has been widely used in oncology and has shown exceptional predictive performance in prognoses [16–18].

However, a major obstacle to the clinical application of radiomics and deep learning models is their limited interpretability [9, 15]. Most current studies focus predominantly on improving predictive accuracy, yet provide little insight into the underlying biological rationale of survival-associated features. This disconnection hampers clinical translation and limits the ability to guide personalized treatment strategies. Therefore, investigating the biological basis behind survival signatures is a pressing need for future studies. Emerging studies have begun to explore the biological basis of radiomics and deep learning features, and explainable AI has been recognized as a future research topic [19, 20]. However, these studies varied in their quality. To the best of our knowledge, no comprehensive review has yet systematically summarized the current findings or critically discussed their implications. This gap hinders researchers and clinicians to obtain a clear overview of the progress achieved and the challenges that remain. Therefore, this study aims to provide a systematic summary of the available evidence and pinpoint crucial future research directions.

This study investigates the potential biological foundations of prognostic radiomics and deep learning models through a systematic review of existing research, thereby paving the way for enhanced clinical practice and personalized treatment approaches.

## Methods

### Protocol and registration

This systematic review was implemented in accordance with the Preferred Reporting Items for Systematic Reviews and Meta-analyses (PRISMA) statement [21]. Prior to commencement, the review protocol was registered with the International Prospective Register of Systematic Reviews (PROSPERO; CRD420250656208).

### Data sources and search strategy

Using search terms related to breast cancer, artificial intelligence, deep learning, genomics, and prognosis, a comprehensive search was conducted in the PubMed, Cochrane Library, Embase, and Web of Science databases between their inception and Feburary 25, 2025, to find studies published in English. The search terms used were MeSH and free-text synonyms connected with logical operators. Details of the complete search strategy were displayed in the supplementary materials. We manually screened the reference lists of relevant systematic reviews and included articles to identify additional eligible studies.

### Eligibility criteria and study selection

Only original research corresponding to our study’s topic could be included without restrictions on the country of publication source. The inclusion criteria are: (1) Studies involving breast cancer patients undergoing MRI scans. (2) Studies developing and validating prognostic radiomics or deep learning models. (3) Studies uncovering the biological underpinnings behind the prognostic radiomics or DL features. The exclusion criteria involved: (1) systematic reviews, conference abstracts, guidelines, and other analogous studies. (2) The subjects of studies included animals, or other types of cancers. (3) Studies focusing solely on unrelated topics instead of prognosis, such as diagnosis or classification. (4) Studies without radiomics or deep learning models. (5) Prediction models influenced by genomics or lacking biological analysis based on prognostic signatures.

### Data extraction

Two authors independently conducted data extraction with a standardized protocol, and extracted information including the following: (1) Study baseline characteristics: first authors, publication year, study country, study institution, study design, and sample size; (2) MRI image parameters and details of radiomics or deep learning model: MRI image parameters, treatment methods, prognostic outcomes, methods and software for segmentation and features extraction, number of features extracted, cutoff analysis, and results for biological correlation analysis; (3) Biological data: features/clusters/survival models to explore the biological basis, data sources for samples, and types of biological information; (4) Methods of investigating the biological meaning. Subsequently, the results were cross-checked by two authors, and any discrepancies were addressed through consultation with a third author.

### Quality assessment

Radiomics Quality Score (RQS) [22], Newcastle-Ottawa Scale (NOS) [23]and Quality Assessment of Prognostic Accuracy Studies (QUAPAS) [24]were adopted to evaluate the methodological quality of each study. The RQS assessment contains 16 key components. The total scores ranged from − 8 to 36, which aims to minimize bias and enhance the applicability of prediction models [22]. As an “easy to use” quality score, NOS scale contains three dimensions including selection, comparability, and—depending on the study type—outcome (cohort studies) or exposure (case-control studies) [23]QUAPAS is an adaptation of Quality Assessment of Diagnostic Accuracy Studies-2 (QUADAS-2) to assess risk of bias (ROB) and applicability in systematic reviews of prognostic accuracy studies, encompassing five domains: participants, index test, outcome, flow and timing, and analysis [24]. Two reviewers assessed the risk of bias in all enrolled studies and any disagreement was resolved by discussion and consensus with the guidance of third investigator. This study utilized the Intraclass Correlation Coefficient (ICC) and Kappa statistics to assess the inter-rater reliability.

## Results

### Study selection

**Fig. 1:**
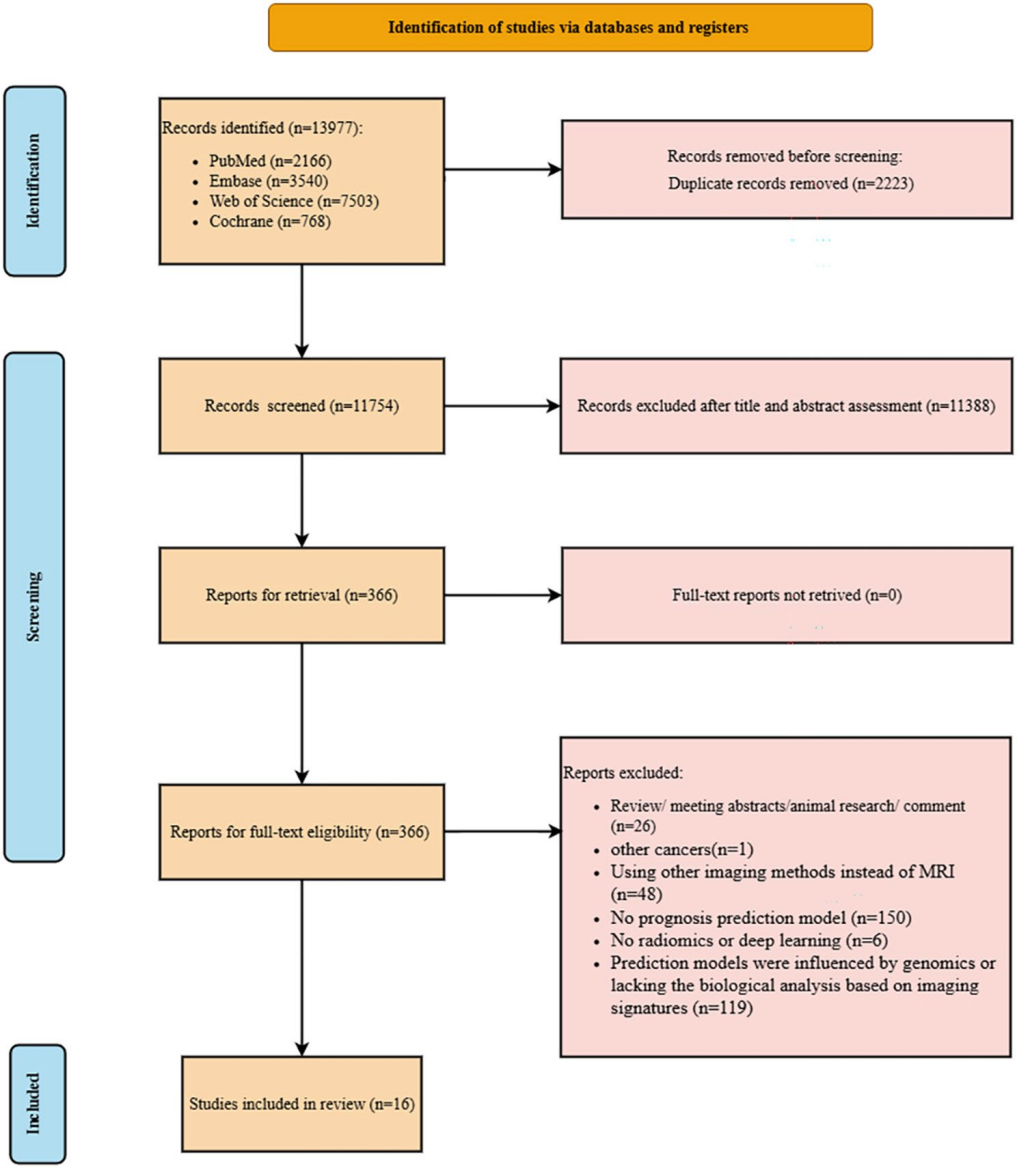
Flowchart of the study screening and selection process

The initial literature search collected 13,977 papers from four main databases, and subsequently, duplicate papers were removed. After initial screening using the title and abstract of the literature, 366 papers were identified for full-text review. Ultimately, 16 eligible papers were included in this systematic review [25–40]. Figure 1 illustrates the detailed flowchart of literature screening.

### General characteristics of eligible studies

The general characteristics of the included studies are depicted Table 1. All studies were published between 2015 and 2025, with the majority between 2022 and 2024. In terms of sample size, the number of patients ranged from 61 to 2279. Most studies were from China [27, 40], while only two studies were conducted in the US [25, 26]. While all included studies were retrospective, the predictive models from two investigations were further validated in prospective cohorts [28, 39]. Six studies were multi-center [28, 30, 31, 36, 39, 40], four were single-center [25, 26, 29, 35], and the remaining six were based on public databases [27, 32, 34, 37, 38].

**Table 1:** General characteristics of included studies

study ID	Country	Study design	Institution	Data sources for samples	No.patient	No.cohort	type of cohort(training/validation)	type of patient	type of treatment	prognostic outcome
Shota Yamamoto, 2015 [25]	US	R	1 center*	LncRNA from tumor tissues	61	2	training set(*n* = 19) validation(*n* = 42)	IBC stage IIIC or lower	surgery or surgery + CT	MFS
Jia Wu, 2017 [26]	US	R	1 center&+TCIA(TCGA-BRCA) + GEO(GSE1456)	TCGA: RNA-seq data of tumor and adjacent parenchyma from frozen tissue samples GSE1456: microarray gene expression data from frozen tumor tissue obtained by surgical excision	1314	4	imaging biomarker discovery cohort(*n* = 60) TCGA-training corhort(*n* = 126) TCGA-testing cohort(*n* = 879) GSE1456 cohort(*n* = 159)	imaging biomarker discovery cohort: stage II or III LABC TCGA, GSE1456: IBC	imaging biomarker discovery cohort: NACT + surgery TCGA: pharmaceutical therapy/ RT/ surgery GSE1456:NA	RFS, OS
Ming Fan, 2019 [27]	China	R	TCIA(Breast MRI-NACT pilot; ISPY-1; TCGA-BRCA)	reproducibility cohort: gene expression data from frozen primary tumors TCGA: RNA-seq data from frozen primary tumor samples	321	4	training cohort(*n* = 61) reproducibility cohort(*n* = 173) radiogenomic cohort(*n* = 87) TCGA cohort(*n* = 1010)	training: stage II or III LABC reproducibility: stage II or III IBC radiogenomic, TCGA: IBC	training: NACT + surgery reproducibility: NACT + surgery radiogenomic, TCGA: pharmaceutical therapy/ RT/ surgery	RFS, OS
Yunfang Yu, 2021 [28]	China	P-R	4 centers^@^+ TCIA(TCGA-BRCA)	TCGA: transcriptome data from frozen primary tumor tissues	1179	4	training cohort (*n* = 803) prospective-retrospective validation cohort (*n* = 106) external validation cohort (*n* = 179) TCGA cohort (*n* = 91)	training cohort, prospective-retrospective validation cohort: stage I-III IBC TCGA: IBC	training cohort, prospective-retrospective validation cohort: surgery TCGA: pharmaceutical therapy/ RT/ surgery	ALN status
Xuanyi Wang, 2022 [29]	China	R	1 center (FUSCC) + TCIA(TCGA-BRCA) + GSE118527	TCGA: RNA-seq and microRNA-seq data from frozen primary tumor specimens GSE118527: RNA-seq data from fresh frozen primary tumor tissues	729	4	training set(*n* = 139) validation set(*n* = 139) external validation cohort (*n* = 91) FUSCC-TNBC cohort (*n* = 360)	training, validation cohort: clinical stage II–III LABC external validation cohort : IBC FUSCC-TNBC cohort : TNBC	trianing, validation: NACT and postoperative RT/ HER2-targeted therapy/ HT external validation, FUSCC-TNBC: pharmaceutical therapy/ RT/ surgery	DFS, RFS
Wenlong Ming, 2022 [30]	China	R	local database + TCIA(TCGA-BRCA) + GEO (GSE1456, GSE3494, GSE7390, GSE20685, GSE25055, GSE25065)	imaging-subtype discovery cohort: RNA-seq data from frozen tumor tissues imaging-subtype validation cohort: RNA-seq data from frozen primary tumor specimens GSE1456: gene expression data from frozen tumor tissues GSE3494: gene expression data from freshly frozen breast tumors GSE7390: gene expression profiling data from frozen samples GSE20685: gene expression profiles data from breast cancer samples GSE25055,GSE25065: gene expression data from pre-treatment tumor biopsies	1689	8	imaging-subtype discovery cohort (*n* = 174) imaging-subtype validation cohort (*n* = 72) six external datasets (*n* = 1443)	imaging-subtype discovery cohort, imaging-subtype validation cohort: stageI-III IBC GSE1456: stageI-III IBC GSE3494: primary breast cancers GSE7390: node-negative, T1-T2 (≤ 5 cm) GSE20685:IBC GSE25055/GSE25065: HER2-negative IBC	imaging-subtype discovery cohort and imaging-subtype validation cohort: NA GSE1456: surgery + pharmacotherapy/RT GSE3494: the Uppsala cohort (*n* = 67): surgery + adjuvent therapy; others: NA GSE7390:untreated GSE20685: sugery + RT/adjuvant CT/ HT/ NACT GSE25055/GSE25065: surgery + adjuvant therapy/ ET	DFS, OS
Yunfang YU, 2023 [31]	China	R	4center$	RNA-seq from FFPE biopsy tissues	1113	3	training cohort (*n* = 698) validation cohort (*n* = 171) testing cohort (*n* = 244)	stage I–III IBC	surgery + ET/HER2-targeted therapy/NACT	RFS
Guan-Hua Su, 2023 [32]	China	R	1 center (FUSCC) + DUKE + TCIA(TCGA-BRCA)	multi-omics data—including RNA sequencing, genomic DNA, total RNA, polar metabolites, and lipid metabolites—derived from fresh-frozen tumor tissues	1474	3	FUCSS cohort (*n* = 711), DUKE cohort (*n* = 641) TCGA cohort (*n* = 122)	FUCSS, DUKE, TCGA: IBC	FUCSS: NA DUKE: NACT/ surgery/CT/ HER2-targeted therapy/RT/ET TCGA: pharmaceutical therapy/ RT/ surgery	RFS, OS
Chao You, 2024 [33]	Chnia	R	1centers(FUSCC) + DUKE + ISPY-1	RNA sequencing, genomic DNA, polar metabolites, Lipid metabolites of multiomic data fresh frozen tumor tissues	762	3	FUSCC cohort (*n* = 420) DUKE cohort (*n* = 217) ISPY-1 cohort (*n* = 125)	FUSCC, DUKE: IBC ISPY-1: stage 2 or 3 IBC	FUSCC: untreated DUKE: NACT/ surgery/CT/HER2-targeted therapy/RT/ET ISPY-1: NACT + surgery	RFS, OS
Xinyu Zhang, 2024 [34]	China	R	ISPY-2 (GSE194040)#	mRNA expression data from fresh-frozen pre-treatment breast cancer tumors	785	2	training corhort (*n* = 588) testing corhort (*n* = 197)	stage II or stage III LABC	NACT + surgery	pCR
Ming Fan, 2024 [35]	China	R	1 center + ISPY-1 + GEO (GSE32063)	gene expression data were obtained from both post-treatment biopsy specimens (24–72 h after initiation) and from tumor specimens surgically resected after CT	429	2	development dataset (*n* = 255) prognostic validation dataset (*n* = 174)	development dataset: IBC prognostic validation dataset: stage 2 or 3 IBC	NACT + surgery	pCR, CS
Mingping Hong, 2024 [36]	China	R	4 center + TCIA(TCGA-BRCA) + DUKE	TCGA: RNA transcriptomics data from frozen primary tumor specimen	1536	6	training cohort (*n* = 532) three testing cohorts (*n* = 113,185,381) genomics cohort (*n* = 99) prognostic cohort (*n* = 226)	training and testing: early-stage IBC genomics/ prognosis cohort: IBC	training and testing: NA genomics: pharmaceutical therapy/ RT/ surgery prognosis: NACT/ surgery/ CT/ HER2-targeted therapy/ RT/ ET	ALN status
Xue Li, 2024 [37]	China	R	TCIA-TCGA	mRNA expression data from frozen primary tumor specimens	90	2	training cohort(*n* = 62) testing cohort(*n* = 28)	IBC	pharmaceutical therapy/ RT/ surgery	ALNM
Wenci Liu, 2024 [38]	China	R	TCIA(TCGA-BRCA, Breast-MRI-NACT Pilot)	TCGA: RNA transcriptomics data from cohort frozen primary tumor specimens	145	2	training cohort, *n* = 88 validation cohort, *n* = 57	TCGA: IBC Breast-MRI-NACT Pilot: stage II or III LABC	TCGA: pharmaceutical therapy/ RT/ surgery Breast NACT-Pilot: NACT + surgery	LNM
Ziyin Li, 2024 [39]	China	R-P	6 centers^**+**^	RNA sequencing data from biopsy tissue samples	1145	5	training and validation corhorts (*n* = 506) internal test corhort(*n* = 127) pooled external test corhort (*n* = 414) prospective corhort(*n* = 98)	IBC with ipsilateral ALN metastasis	NACT + surgery, NACT	axillary pCR
Yuhong Huang, 2025 [40]	China	R	12 centers	RNA-seq and scRNA-seq of fresh tumor samples were obtained from using ultrasound-guided fine-needle aspiration before treatment	2279	4	training cohort (*n* = 431) three validation cohorts (*n* = 1595) the immunotherapy cohort (*n* = 88) multi-omics cohort (*n* = 165)	unilateral IBC	NACT + surgery	pCR

*:The Seoul National University Hospital; &:University of Washington Breast Cancer Specialty Center; %: the First Affiliated Hospital of Nanjing Medical University; @:Sun Yat-sen Memorial Hospital of Sun Yat-sen University; Sun Yat-sen University Cancer Center; Shunde Hospital of Southern Medical University, and Tungwah Hospital of Sun Yat-sen University; $:the national hospitals Sun Yat-sen Memorial Hospital of Sun Yat-sen University, Sun Yat-sen University Cancer center, the Shunde Hospital of Southern Medical University, the Tungwah Hospital of Sun Yat-sen University; #: over 22 clinical study sites around the USA; **+**, Fudan University Shanghai Cancer Center, Guilin Municipal Hospital of Traditional Chinese Medicine, Beijing Cancer Hospital, Affiliated Hospital of Qingdao University, and Binzhou Medical University Hospital, Yantai Yuhuangding Hospital

FUSCC, The Fudan University Shanghai Cancer Center; IBC, invasive breast cancer; LABC, locally advanced invasive breast cancer; CT, chemotherapy; NACT, neoadjuvant chemotherapy; PORT, Postoperative radiotherapy; RT, radiotherapy; ET, endocrine therapy; MFS, metastasis-free survival; RFS, recurrence-free survival; OS, overall survival; DFS, Disease-free survival; pCR, Pathological complete response; CS, concentric shrinkage; NA, no avaliable. ALN, Axillary Lymph Node; FFPE, formalin-fixed paraffin-embedded; ALNM, Axillary Lymph Node Metastasis; LNM, Lymph Node Metastasis

Among the included studies, all of them focused on invasive breast cancer regardless of its subtype. Additionally, patients’ treatments were diverse, including surgery, chemotherapy, radiotherapy, or surgery combined with chemotherapy or radiotherapy. We also found that recurrence-free survival (RFS) was frequently used as a key indicator for assessing prognostic outcomes in six studies [26, 27, 29, 31–33]. Furthermore, other indicators such as overall survival (OS), disease-free survival (DFS), metastasis-free survival (MFS), pathological complete response (pCR) and Axillary lymph node metastasis (ALNM) were adopted independently or in conjunction with RFS [25, 26, 28, 30, 32, 40]. It is worthwhile to notice that every article included a development and validation cohort and some even owned external validation cohorts.

### Characteristics of model construction

Regarding the region of interest (ROI) segmentation method, three articles utilized manual methods [27, 29, 38], ten employed semi-automatic methods [26, 28, 30–34, 36, 40], and two utilized an automatic method [35, 39]. 3D-slicer and Pyradiomics were the most leveraged software for segmentation or extracting image features [28–35, 37, 38].

Among articles that constructed predictive models [25–29, 31, 33–40], most of them developed prognostic radiomics models by supervised machine learning methods, utilizing the Cox proportional hazards model or random forest for model construction. Meanwhile, cutoff values in two articles were identified by median value [25, 33], while cutoff values in three articles were identified by the smallest log-rank P value [26, 27, 35]. In addition, three studies [29, 36, 39] selected the Youden index as optimal thresholds to stratify different risk groups, while other three studies applied deep learning methods. Specifically, Yu et al. [31] employed “DeepSurv” to develop the RDeepNet model for predicting individual recurrence risk, with cutoff values determined using the R package ggsurvminer. Two deep-learning models, GoogLeNet and ResNet50, were trained for predicting axillary lymph-node metastasis by Liu et al. [38]. Additionally, Li et al. [39] also constructed a fully automated integrated system based on deep learning (FAIS-DL).

In contrast to the supervised studies, clustering analyses were conducted in all unsupervised studies, although their specific methods varied. For instance, Ming et al. [30] adopted the consensus clustering algorithm to identify three de novo subtypes., while Su et al. [32]conducted spectral clustering to pinpoint the optimal number of clusters (k = 2).

### Acquisition of biological information

Among the included studies, samples from frozen primary tumor tissues were utilized, with these biospecimens typically obtained through surgical resection or needle biopsy. Concerning the types of biological data analyzed, all articles investigated transcriptomics data [25, 40]. A study by Yamamoto et al. [25] determined the lncRNA expression through extracting next-generation RNA sequencing. Furthermore, Su et al. [32], You et al. [33] and Li et al. [39] also investigated the multi-omics information related to genomic DNA, polar metabolites, pathology, and lipid metabolites, whereas transcriptomic data and immune microenvironment were reported in six studies [28–31, 36, 40].

### Strategies for feature-based biological analysis

Various biological analyses were adopted in these included studies to explore the potential biological meaning of prognostic signatures. We found that it was universal to leverage gene set enrichment analysis (GSEA) or other functional enrichment analysis to identify enriched Kyoto Encyclopedia of Genes and Genomes (KEGG) pathways, Gene Ontology (GO) Biological Processes or Hallmark gene sets. Three studies [31, 37, 38] utilized gene set variation analysis (GSVA) to identify enriched pathways, among which two studies also employed Weighted Gene Co-expression Network Analysis (WGCNA) [37, 38]. In comparison, to assess the immune cell composition, CIBERSORT was used by three studies [28–30] and the Microenvironment Cell Populations-counter (MCP-counter) adopted by Fan et al. [35] and Huang et al. [40]. It is worth mentioning that three studies performed a comprehensive multiomics analysis to inquire about the biological foundation of radiomic signatures, including metabolomic, methylation, and pathological pathways. Various statistical methods, including correlation analysis and differential analysis (e.g., Student’s t test, Wilcoxon rank-sum test, Kolmogorov–Smirnov test, ANOVA, and Fisher’s exact test), were applied to explore the potential biological basis of imaging biomarkers [25, 27, 31–34, 37, 39, 40]. The radiomic and biological information are summarized in Table 2.

**Table 2:** Radiomics and biological information of the included studies

study ID	segmentation method	Software(segmentation or features extracted)	No. Radiomics features extracted	Raiomics features selection methods	No. Radiomics features in the final survival model	Cut-off analysis	Outcomes for Biological Relevance Analysis	Futures/Clusters/Survival models to explore the biological basis	Statistic method to investigate the biological meaning	type of biological information
Shota Yamamoto, 2015 [25]	semi-automatic	ImagePrism4D	47	Pearson correlation, Cox regression analysis	21	median value	model	1(radiomics model)	differential expression analysis+; Spearman correlation analysis; lncRNA pathway enrichment analysis	LncRNA expression, pathways
Jia Wu, 2017 [26]	semi-automatic	MATLAB	10	Pearson correlation analysis, multivariate Cox regression model	10	the smallest log-rank P value	features	1 feature	KEGG pathway enrichment analysis	gene module, pathways
Ming Fan, 2019 [27]	semi-automatic	MATLAB	14	Pearson correlation coefficient, a multivariate Cox regression analysis	5	the smallest log-rank p value	features	3 features	WGCNA; Pearson correlation analysis; KEGG pathway analysis	gene module, KEGG pathways
Yunfang Yu, 2021 [28]	semi-automatic	3D-Slicer, PyRadiomics	5178	t-test, random forest algorithm	180	the R package cutpointr	features, model	138 features, 1 (radiomics model)	The CIBERSORT algorithm, differential analysis, GO and KEGG enrichiment analyses, Random forest algorithm, correlation analysis	immune cells(M0 macrophages, B naïve cells, and neutrophils), types of methylated sites, corrlated long non-coding RNAs DEGs, GO terms, KEGG pathways
Xuanyi Wang, 2022 [29]	manual	3D-Slicer, PyRadiomics	850	ICC, Univarable Cox proportional harzards model, LASSO	15	Youden index	Model	1(radiomics model)	GSEA,differential expression analysis GO enrichment and KEGG pathway analyses; ESTIMATE method; The CIBERSORTx web tool; differential analysis	DEGs(mRNA, lncRNA, and miRNA), GO terms, KEGG pathways, immunophenotype difference
Wenlong Ming, 2022 [30]	semi-automatic	3D-Slicer, Pyradiomics	174	Unsupervised the consensus clustering algorithm(k = 3)	NA	NA	clusters	3 clusters	RNA Sequencing and Transcriptomic Analysis^$^	DEGs, pathways, the abundance of 15 cell types
Yunfang Yu, 2023 [31]	semi-automatic	3D-Slicer, Pyradiomics	3452	NA	3452	the R package ggsurvimier	Model	1(deep learning model)	ssGSEA algorithm, The t test and limma package, Spearman’s rank correlation analysis, GO and KEGG analyses, GSVA, variation analysis of immune cells	DEGs, GO terms, KEGG pathways, immune cells
Guan-Hua Su, 2023 [32]	semi-automatic	3D-Slicer, PyRadiomics	1968	spectral Clustering (k = 2)	NA	NA	clusters	2 clusters	DA score, GSEA, ssGSEA, Comprehensive metabolomic and lipidomic analyses	the abundance of polar metabolites and lipids, pathways, expression profiles of biological processes crucial for provoking ferroptosis
Chao You, 2024 [33]	semi-automatic	3D-Slicer, Pyradiomics	120	Lasso-Cox regression	13	median value	model	1(radiomics model)	GSEA and ssGSEA, DA score, differential analysis	gene mutation frequency, the abundance of polar metabolites and lipids, pathways
Xinyu Zhang, 2024 [34]	semi-automatic	NA; Pyradiomics	C: 660;D: 1232	ICC, Variances analysis, MannWhitney U test, LASSO regression, mRMR	C:4; D:10	An absolute log-2 fold change larger than 0.25 and a p value smaller than 0.05	model	1(dynamic radiomic model)	GSEA, DEGs analysis	DEGs, KEGG pathways, GO terms
Ming Fan, 2024 [35]	automatic	NA, PyRadiomics	102(each image sequence)	multitask RF model	17	the smallest log-rank P value	model	1(radiomics model)	GSEA, correlation analysis	pathways; genes
Mingping Hong, 2024 [36]	semi-automatic	The U2Net network architecture deep learning model, PyRadiomics	944	independent sample t-tests or Mann–Whitney U tests, correlation analysis, elastic-logistic regression analysis	9	the maximum Youden index and minimum log-rank P value	model	1(deep learning model)	DEGs analysis, GO enrichment analysis, GSEA, immune infiltration analysis-correlation analysis	DEGs, pathways immune cell scores eight types of RNA-based immune markers
Xue Li, 2024 [37]	NA	NA, PyRadiomics	455	LASSO algorithm	13	NA	features	13 features	WGCNA Functional Enrichment Analysis; GSVA, correlation analysis	gene module, GO Biological Processes, pathways, Reactome Gene Sets, gene activation scores
Wenci Liu, 2024 [38]	manual	ITK-snap, PyRadiomics	radiomics: 1781 GoogLeNetnet:1024 ResNet50:2048	PCA relief algorithm	Radiomics: 41 GoogLeNetnet: 67 ResNet50: 39	Radiomics: RF GoogLeNetnet: NA ResNet50: NA	model	1 (DLRN model)	WGCNA KEGG pathway analysis GSVA	gene module, pathways
Ziyin Li, 2024 [39]	automatic	FAIS-DL	NA	NA	NA	The Youden index	model	1 (FAIS-DL model)	DEGs analysis, gene enriched pathways, immune microenvironment analyses, Wilcoxon rank-sum test	DEGs, pathways, the proportions of distinct cell types within a mixed cellular population, immunohistochemical results
Yu Hong Huang, 2025 [40]	manual	3D-Slicer, NA	9784*	ICC > 0.8, Mann–Whitney U test, Spearman correlation analysis, LASSO regression	32^@^	NA	model, features	15 features 1(radiomics model)	DEGs analysis, GSEA, immune cell infiltration analysis, correlation analysis, Pathway enrichment analysis, ssGSEA	DEGs, immune cell infiltration indices, pathways, immune cell makeup and B cell enrichment

*:1223 pre-NAT TR, 3669 pre-NAT SHR, 1223 mid-NAT TR, and 3669 mid-NAT SHR features; +: ifferentially abundant lncRNA transcripts; C:convential radiomics model features; $:DESeq2, ANOVA, GSEA, CIBERSORT, The Student’s t-test; D:dynamic radiomics model features, @: 8 pre-NAT TR, 7 pre-NAT SHR, 9 mid-NAT TR, and 8 mid-NAT SHR features

LASSO, The least absolute shrinkage and selection operator; mRMR, maximum relevance algorithm; RF, random forest; KEGG, Kyoto Encyclopedia of Genes and Genomes; GSEA, gene set enrichment analysis; GO, Gene Ontology; ANOVA, Analysis of variance; ssGSEA, single sample gene set enrichment analysis; GSVA, Gene Set Variation Analysis; UMAP, clustering with Uniform Manifold Approximation and Projection; DA, Differential abundance; WGCNA, weighted gene coexpression network analysis; DEGs, differentially expressed genes; TMB, tumor mutational burden; MCP-counter, Microenvironment Cell Populations-counter; FAIS-DL, fully automated integrated system based on deep learning

### Interpretation of biological outcomes

Differentially expressed genes and KEGG pathways have been investigated in prior studies. For example, Zhang et al. [34] performed the DEG analysis to identify 196 up-regulated genes and 101 down-regulated genes in high-risk group and many enriched pathways in GSEA by KEGG were related to immune system. Similarly, Huang et al. [40] implemented GSEA analysis that highlighted significant differences in chromosome organization and immune response between the two groups.

Five studies [28, 36–39] have identified that metabolic and cell adhesion pathways were associated with ALNM. Apart from the genome and pathways, seven studies also investigated the tumor microenvironment (TME), mainly focusing on immune cell composition [28–31, 36, 39, 40]. As an example, Yu et al. [31] explored the association between radiomic features and TME by performing a variation analysis of immune cells, which revealed differential immune cell abundance between high- and low-risk groups. Additionally, You et al. [33] and Su et al. [32] also studied the abundance of polar metabolites and lipids, while Yamamoto et al. [25] focused on the expression of lncRNA.

### Quality appraisal

**Fig. 2:**
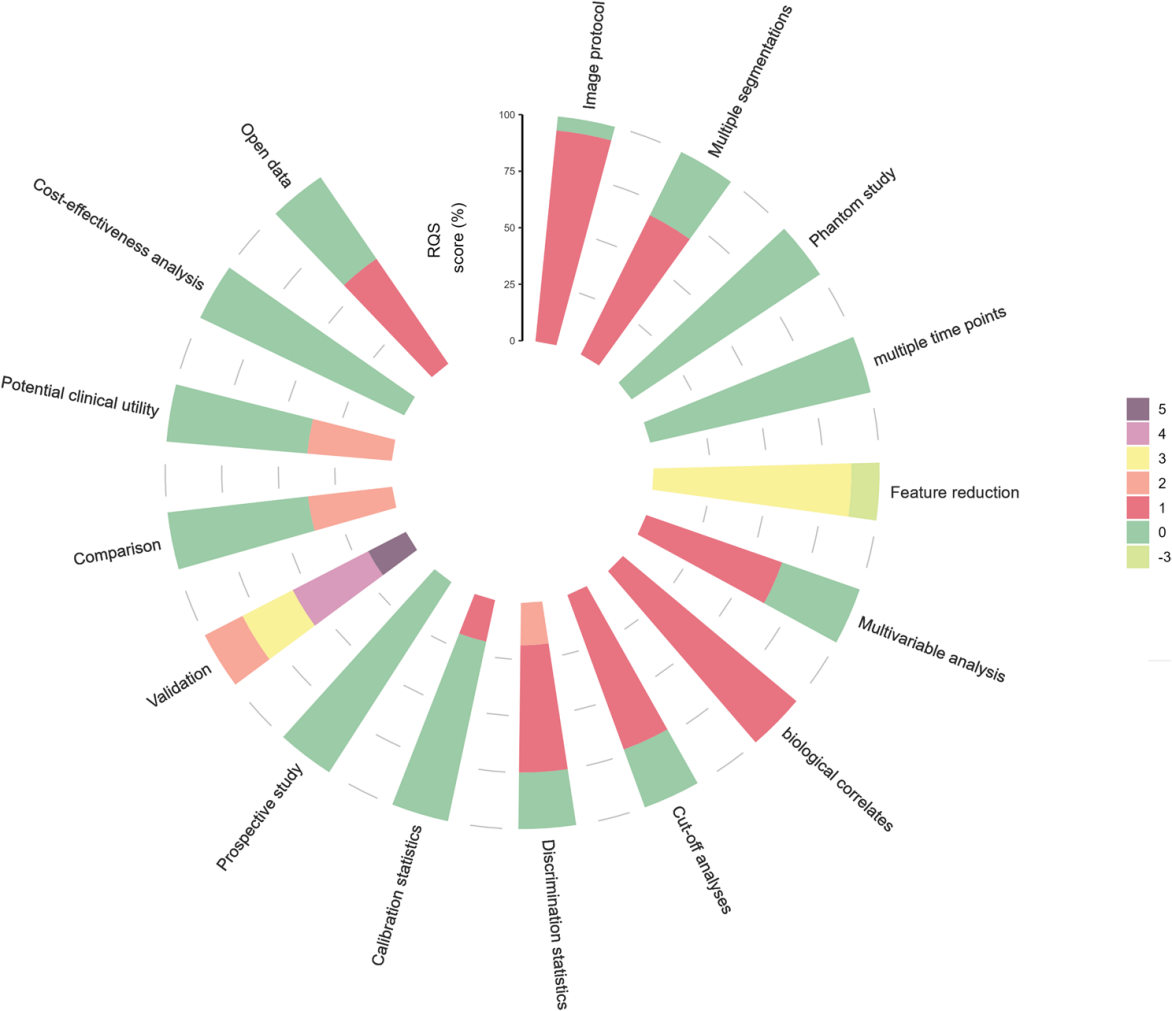
The radiomic quality scores percentage of scores of included studies

Although the number of published articles describing the use of radiomics and deep learning to understand breast cancer is increasing, the overall quality of research on their biological significance remains suboptimal. The median RQS across all included studies was 12.5 (range: 5–17), corresponding to a percentage of 35% (range: 14%-47%). Table 3 presents the total RQS, the percentage of RQS for each study, and the individual scores for each of the 16 components. Figure 2 illustrates the percentage scores for each of the 16 RQS components across the studies.

**Table 3:** Radiomics quality scores for the included studies

RQS Items	Total scores	Shota Yamamoto, 2015 [25]	Jia Wu,2017 [26]	Ming Fan,2019 [27]	Yunfang Yu, 2021 [28]	Xuanyi Wang, 2022 [29]	Wenlong Ming,2022 [30]	Yunfang YU, 2023 [31]	Guan-Hua Su, 2023 [32]	Chao You, 2024 [33]	Xinyu Zhang,2024 [34]	Ming Fan, 2024 [35]	Mingping Hong, 2024 [36]	Xue Li, 2024 [37]	Wenci Liu, 2024 [38]	Ziyin Li,2024 [39]	Yuhong Huang,2025 [40]
Image protocol quality	2	1	1	1	1	0	1	1	1	1	1	1	1	1	1	1	1
Imaging at multiple time points	1	0	0	0	0	0	0	0	0	0	0	0	0	0	0	0	0
Phantom study on all scanners	1	0	0	0	0	0	0	0	0	0	0	0	0	0	0	0	0
Multiple segmentations	1	1	1	0	1	1	1	0	0	1	1	0	1	0	1	1	1
Feature reduction	3 or -3	3	3	3	3	3	3	-3	3	3	3	3	3	3	3	-3	3
Multivariable analysis	1	0	0	0	1	1	1	0	1	1	1	1	1	0	0	1	1
biological correlates	1	1	1	1	1	1	1	1	1	1	1	1	1	1	1	1	1
Cut-off analyses	1	1	1	1	1	1	0	1	0	1	1	1	1	0	1	1	0
Discrimination statistics	2	0	0	0	1	1	1	1	0	2	1	2	1	1	1	2	1
Calibration statistics	2	0	0	0	0	1	0	0	0	0	0	0	0	1	1	0	0
Prospective study	0 or 7	0	0	0	0	0	0	0	0	0	0	0	0	0	0	0	0
Validation	5 or -5	2	4	4	4	3	3	4	4	4	2	3	5	2	3	5	5
Comparison to ‘gold standard’	2	0	0	0	0	2	0	0	2	2	0	0	2	0	0	2	2
Potential clinical utility	2	0	0	0	2	2	0	0	0	0	2	0	0	2	2	0	2
Cost-effectiveness analysis	1	0	0	0	0	0	0	0	0	0	0	0	0	0	0	0	0
Open data	4	0	0	1	1	0	0	0	1	1	1	0	1	1	1	1	0
% RQS (total score)	100(36)	25(9)	31(11)	31 (11)	44(16)	44(16)	31(11)	14(5)	36(13)	44(16)	39(14)	33(12)	47(17)	33(12)	42(15)	33(12)	47(17)

**Fig. 3:**
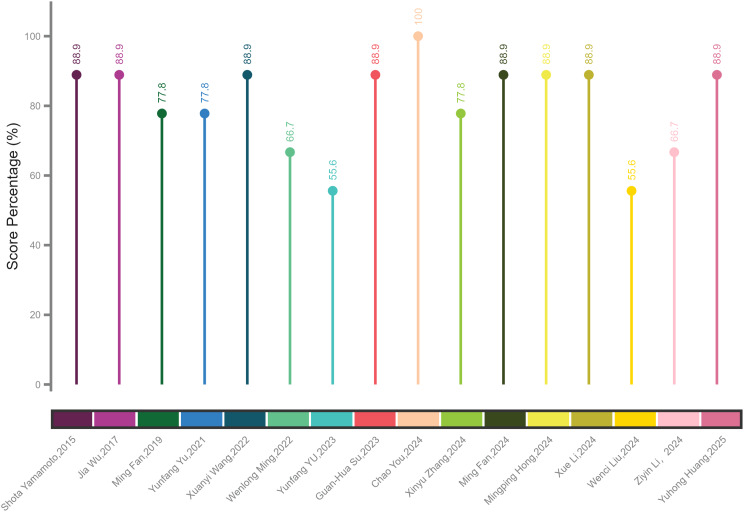
Percentage of average score fulfilling the Newcastle-Ottawa Scale in the included studies

**Fig. 4:**
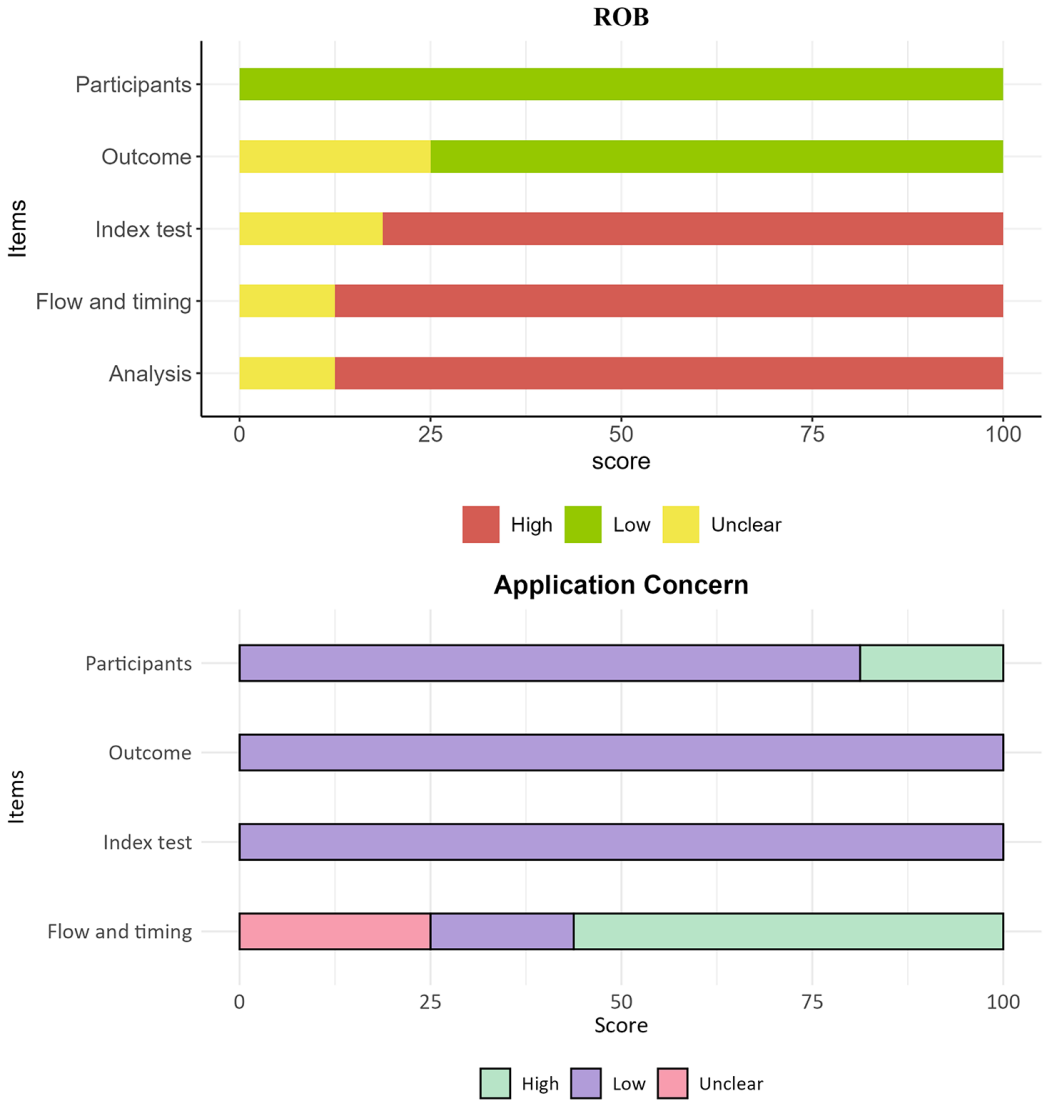
The risk of bias and applicability concerns of the included studies using the quality assessment of prognostic accuracy studies

Table S1 displays the NOS (Newcastle-Ottawa Scale) score for 16 of the included studies. Figure 3 shows the percentage of the NOS score fulfilled by each study, with a mean score of 7.3 (81.1% of the total score of 9). Areas with the lowest scores included evaluation, data, and reference standards. On the other hand, study design, data partitions, and model development scored the highest. According to the QUAPAS guidelines, all studies were categorized as having a high risk of bias (ROB) (Fig. 4; Table 4). Additionally, all domains showed low concern for applicability, except for domain 1. Notably, two studies focused solely on patients with locally advanced breast cancer and triple-negative breast cancer (TNBC), raising concerns about the generalizability of the findings. In the concordance analysis, there was good agreement between the two reviewers (ICC > 0.75, average Kappa > 0.8). The details can be found in the supplementary materials.

**Table 4:** Quality assessment of prognostic accuracy studies assessment results of included studies

Study ID	Risk of Bias					Applicability Concern				Overall
	Participants	Index Test	Outcome	Flow and Timing	Analysis	Participants	Index Test	Outcome	Flow and Timing	
Shota Yamamoto, 2015 [25]	Low	High	Low	High	High	High	Low	Low	Low	High
Jia Wu, 2017 [26]	Low	High	Unclear	High	High	Low	Low	Low	High	High
Ming Fan, 2019 [27]	Low	High	Low	High	High	Low	Low	Low	Low	High
Yunfang Yu, 2021 [28]	Low	High	Low	Unclear	High	Low	Low	Low	Low	High
Xuanyi Wang, 2022 [29]	Low	Unclear	Low	Unclear	High	Low	Low	Low	Unclear	High
Wenlong Ming, 2022 [30]	Low	High	Unclear	High	Unclear	Low	Low	Low	High	High
Yunfang YU, 2023 [31]	Low	High	Low	High	High	Low	Low	Low	High	High
Guan-Hua Su, 2023 [32]	Low	High	Low	High	High	Low	Low	Low	Low	High
Chao You, 2024 [33]	Low	High	Low	High	High	High	Low	Low	Low	High
Xinyu Zhang, 2024 [34]	Low	High	Low	High	High	Low	Low	Low	Unclear	High
Ming Fan, 2024 [35]	Low	High	Low	High	Unclear	High	Low	Low	Low	High
Mingping Hong, 2024 [36]	Low	High	Low	High	High	Low	Low	Low	Unclear	High
Xue Li, 2024 [37]	Low	Unclear	Unclear	High	High	Low	Low	Low	Low	High
Wenci Liu, 2024 [38]	Low	High	Low	High	High	Low	Low	Low	Low	High
Ziyin Li, 2024 [39]	Low	High	Low	High	High	Low	Low	Low	Low	High
Yuhong Huang, 2025 [40]	Low	High	Unclear	High	High	Low	Low	Low	Low	High

## Discussion

**Fig. 5:**
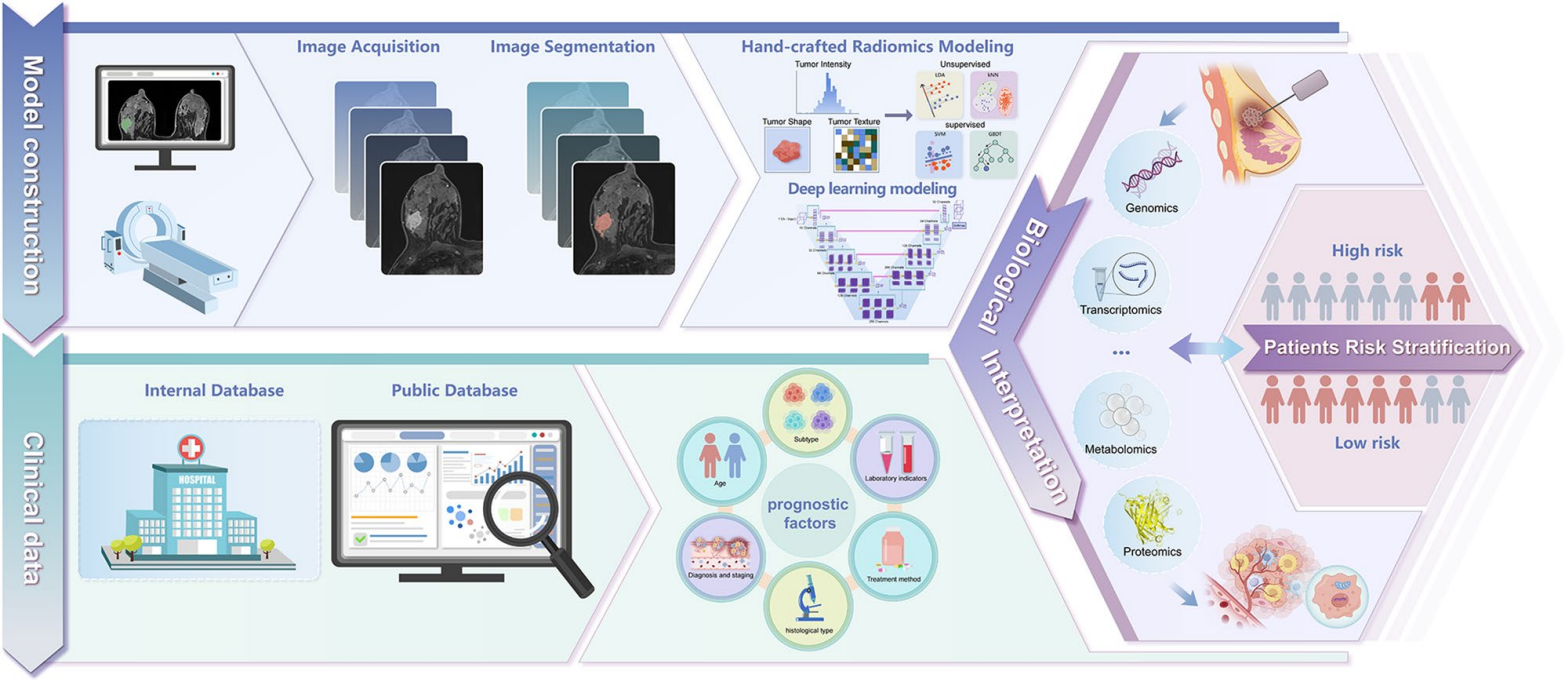
The pipeline of exploration in biological explanation for radiomics or deep learning models in the prognosis of patients with breast cancer. For building radiomic or deep learning models, radiomic features were mainly extracted from MRI images. Then, predictive models were created using various methods, including supervised and unsupervised approaches, as well as deep learning algorithms. Clinical information was also typically collected and integrated into these models. Next, we conducted specialized analysis using various biological data, including genomics, transcriptomics, metabolomics, and proteomics, to investigate the underlying biological mechanisms

We systematically reviewed prognostic studies for breast cancer that used radiomics and deep learning signatures built on the biological foundation and retrieved 16 studies from four main databases (Fig. 5). The results point out the potential to predict prognostic outcomes through data-driven models based on the biological foundation. Moreover, it highlighted significant heterogeneity in the predictive modeling strategies and approaches for biological exploration.

Before conducting biological information analyses, a cut point is always required to dichotomize prognostic biomarker levels of individuals at high risk and those not [41]. An optimal cutoff value considerably impacts the model’s performance, accuracy, and reliability [42, 43]. Conventional fixed cut-point methods, encompassing empirical rules, mean value method, and quantile analysis, are frequently insufficient to handle complex and heterogeneous data distributions [44]. Researchers have developed nuanced and responsive approaches to cut-point settings in response to limitations [45, 46]. Generally, two methods based on the ROC curve, including the Youden index and the point on the ROC curve closest to (0,1), are widely employed for establishing the optimal cut-point. Similarly, X-tile software [47] or R packages (including “survminer,” “CatPredi,” “OptimalCutpoints,” etc.) are common cut-point selection methods. Meanwhile, with the development of machine learning, some data mining tools like Decision Tree (DT) [48] Neural Network (NN) [49] and ensemble-based methods [50] could automatically identify the optimal cutoff value and strengthen the robustness of risk stratification. Notably, intelligent bio-inspired algorithms could be utilized with suitable machine learning algorithms to improve generalization in complex environments and enhance decision-making robustness significantly [51–53]. Each approach has strengths and weaknesses, and the cut-point decision depends on the specific application scenarios.

A clear biological understanding enhances the credibility of imaging findings and facilitates their clinical application. Recent studies have shown that radiomic features correlate with gene expression signatures related to angiogenesis, immune response, and proliferation [34, 54, 55]. Furthermore, transcriptome integration has further linked imaging characteristics to key biological processes, such as cell cycle regulation, DNA repair, and immune-related pathways [25–40]. In parallel, radiomic-based prognostic models have been associated with TME components, including immune cell infiltration, angiogenesis, and extracellular matrix (ECM) remodeling [28, 56, 57]. By connecting imaging features to the tumor microenvironment (TME), these studies have the potential to assess immune status noninvasively and predict immunotherapy response. In contrast, the integration of radiomics with metabolomics in breast cancer is still in its early stages. Only a handful of studies suggest that radiomic features may reflect metabolic diversity, especially in terms of lipid metabolism (such as glycerophospholipid metabolism), glycan biosynthesis, energy metabolism, and iron metabolism. In addition, the integration of genomics, transcriptomics, proteomics, or metabolomics with imaging features provides a comprehensive perspective and capture tumor complexity [58–60]. Aligned with the analysis of included studies [32, 33], highly invasive tumors tended to be highly proliferative and energy-consuming. These integrative approaches facilitate a comprehensive understanding of the biological mechanism behind prognostic imaging features, thereby accelerating the implementation of precision medicine.

Similarly, various approaches could be employed to dive into the potential relevance of radiomics or deep learning features to tumor biology. Fuctional enrichment analysis has emerged as a prevalent method, including GSEA, which is widely utilized [61]. GSEA can identify significantly enriched pathways or gene sets in different risk groups, providing profound insights into the molecular mechanisms of diseases [62, 63]. However, because it relies on defined gene sets, GSEA probably fails to discover those gene sets or pathways that have not been defined and is apt to overlook the intricate relationships between genes. Moreover, differential expression genes (DEGs) analysis is effective and universal. Utilized tools to conduct DEGs analysis usually included EdgeR or DESeq2 or MAST, etc [64–66]. Meanwhile, immune infiltration analysis is crucial in understanding tumor immune evasion mechanisms and guiding immunotherapy. For example, Etienne Becht et al. developed MCP-counter [67], which enabled the assessment of immune and stromal components in the tumor, inferring tumor composition and understanding its potential relevance to prognosis. Furthermore, recent studies have indicated that cell-cell communication (CCC) and gene trajectory inference are essential tools for offering deeper insights into complex and variable tumor development based on the single-cell level [68–70].

It is worth mentioning that a study utilized a prospective cohort for external validation of a deep learning model predicting axillary pCR and revealed the connection between FAIS-DL and immunity pathways [39]. Additionally, Yu et al. [28] conducted a multicenter study and validated their model in a prospective–retrospective clinical trial cohort (NCT04003558), thereby confirming its robustness and clinical applicability. They identified two significant features associated with lymph node metastasis(T1 + C-wavelet-LLL-GLCM-Inverse Difference Moment Normalized and DWI-ADC-wavelet-HHH-GLSZM-Large Area Low Gray Level Emphasis), which are linked to immune response and metabolic pathways. Although most signatures are still in the validation phase, these studies show the ongoing efforts to translate radiomics biomarkers into clinical practice.

The included studies exhibited a high ROB. Their generalizability is significantly limited by small sample sizes, reliance on single-center, or unstandardized pipelines. This underlines the urgent need for the establishment of large-scale databases. The Cancer Imaging Archive (TCIA, https://www.cancerimagingarchive.net/) is an exemplary platform for effective data sharing, integrating clinical information and imaging data. Nevertheless, there remains a pressing need for high-quality datasets to advance radiomics research. Besides, variations in MRI sequences may also contribute to inconsistency. Dynamic contrast-enhanced (DCE) MRI was the most widely employed sequence across the included studies. Our research revealed that radiomics features from DCE-MRI are often connected to biological pathways tied to tumor growth, invasion, and metastasis. However, since different MRI sequences highlight unique tissue features, other sequences may hold additional biological insights that have yet to be explored. For instance, DCE-MRI shows tumor angiogenesis and perfusion characteristics, while diffusion-weighted imaging (DWI) and Apparent Diffusion Coefficient (ADC) maps provide a look at tumor biology by evaluating cellular density and tissue microstructural integrity [71]. Therefore, future studies should also place greater emphasis on exploring multi-sequence MRI.

Beyond the above, the study has the following limitations. Firstly, given the risk of confounding radiomics features’ independent contribution with underlying genomic effects, we also excluded studies that used genomic information before biological analysis for feature selection or model construction. However, we recognized that excluding these studies might limit the number of articles we can analyze and introduce potential biases. Secondly, the high ROB and low RQS score of most studies considerably limits the reliability and generalizability of their findings. Ultimately, the number of prospective studies remains limited, and most models or signatures are still at the validation stage.

## Conclusion

This review highlights the connections between imaging features that predict patient outcomes and biological changes, mainly involving genomic and transcriptomic factors. These biological connections support the clinical usefulness of imaging signatures in improving outcomes and tailoring treatment to individual patients with breast cancer. Further validation through large-scale, prospective, and multi-omics studies remains crucial.

## Supplementary Information

Below is the link to the electronic supplementary material.

Supplementary Material 1

## Data Availability

As this is a systematic review, all included subjects have been previously reported in published articles.

## Abbreviations

RQS: Radiomics Quality Score
NOS: Newcastle-Ottawa Scale
QUAPAS: Quality Assessment of Prognostic Accuracy Studies
ROB: Risk of bias
TME: Tumor microenvironment
PRISMA: Preferred Reporting Items for Systematic Reviews and Meta-analyses
QUADAS-2: Quality Assessment of Diagnostic Accuracy Studies-2
OS: Overall survival
RFS: Recurrence-free survival
DFS: Disease-free survival
pCR: Pathological complete response
MFS: Metastasis-free survival
ROI: Region of interest
XGBoost: eXtreme Gradient Boosting
FUSCC: The Fudan University Shanghai Cancer Center
IBC: Invasive breast cancer
LABC: Locally advanced invasive breast cancer
CT: Chemotherapy
NACT: Neoadjuvant chemotherapy
PORT: Postoperative radiotherapy
RT: Radiotherapy
ET: Endocrine therapy
CS: Concentric shrinkage
TCGA: The Cancer Genome Atlas
TCIA: The Cancer Imaging Archive
LASSO: The least absolute shrinkage and selection operator
mRMR: maximum relevance algorithm
RF: Random forest
KEGG: Kyoto Encyclopedia of Genes and Genomes
GSEA: Gene set enrichment analysis
GO: Gene Ontology
ANOVA: Analysis of variance
ssGSEA: single sample gene set enrichment analysis
GSVA: Gene Set Variation Analysis
UMAP: Uniform Manifold Approximation and Projection
DA: Differential abundance
WGCNA: Weighted gene coexpression network analysis
DEGs: Differentially expressed genes
TMB: Tumor mutational burden
TNBC: Triple-negative breast cancer
DT: Decision Tree
NN: Neural Network
MCP-counter: Microenvironment Cell Populations-counter
CCC: Cell-cell communication
IBSI: Image Biomarker Standardization Initiative
ALNM: Axillary lymph node metastasis
