# Supplementary material for: Deciphering the biological underpinnings behind prognostic MRI-based imaging signatures in breast cancer: a systematic review
Source: J Transl Med. 2025 Dec 17;23:1402. doi: 10.1186/s12967-025-07341-1 (PMC12709816; doi:10.1186/s12967-025-07341-1)
Supplement: Supplementary file 1 — Supplementary Material 1 [file 12967_2025_7341_MOESM1_ESM.docx]

**Supplementary Material**

**Search Strategy**

**1.Cochrane search strategy (n = 768)**

#1 MeSH descriptor: [Breast Neoplasms] explode all trees

#2 (Breast Neoplasm):ab,ti,kw OR (Breast Neoplasms):ab,ti,kw OR (Neoplasm, Breast):ab,ti,kw OR (Neoplasms, Breast):ab,ti,kw OR (Breast Tumors):ab,ti,kw OR (Breast Tumor):ab,ti,kw OR (Tumor, Breast):ab,ti,kw OR (Tumors, Breast):ab,ti,kw OR (Breast Cancer):ab,ti,kw OR (Cancer, Breast):ab,ti,kw OR (Cancer of Breast):ab,ti,kw OR (Cancer of the Breast):ab,ti,kw OR (Malignant Neoplasm of Breast):ab,ti,kw OR (Breast Malignant Neoplasm):ab,ti,kw OR (Breast Malignant Neoplasms):ab,ti,kw OR (Malignant Tumor of Breast):ab,ti,kw OR (Breast Malignant Tumor):ab,ti,kw OR (Breast Malignant Tumors):ab,ti,kw OR (Mammary Cancer):ab,ti,kw OR (Cancer, Mammary):ab,ti,kw OR (Cancers, Mammary):ab,ti,kw OR (Mammary Cancers):ab,ti,kw OR (Mammary Neoplasms, Human):ab,ti,kw OR (Human Mammary Neoplasm):ab,ti,kw OR (Human Mammary Neoplasms):ab,ti,kw OR (Neoplasm, Human Mammary):ab,ti,kw OR (Neoplasms, Human Mammary):ab,ti,kw OR (Mammary Neoplasm, Human):ab,ti,kw OR (Breast Carcinoma):ab,ti,kw OR (Breast Carcinomas):ab,ti,kw OR (Carcinoma, Breast):ab,ti,kw OR (Carcinomas, Breast):ab,ti,kw OR (Mammary Carcinoma, Human):ab,ti,kw OR (Carcinoma, Human Mammary):ab,ti,kw OR (Carcinomas, Human Mammary):ab,ti,kw OR (Human Mammary Carcinomas):ab,ti,kw OR (Mammary Carcinomas, Human):ab,ti,kw OR (Human Mammary Carcinoma):ab,ti,kw OR (bilateral breast neoplasm):ab,ti,kw OR (bilateral breast tumor):ab,ti,kw OR (bilateral breast tumour):ab,ti,kw OR (breast gland tumor):ab,ti,kw OR (breast gland tumour):ab,ti,kw OR (breast mass):ab,ti,kw OR (breast neoplasia):ab,ti,kw OR (breast neoplasm):ab,ti,kw OR (breast neoplasms):ab,ti,kw OR (breast neoplasms, male):ab,ti,kw OR (breast tumorigenesis):ab,ti,kw OR (breast tumour):ab,ti,kw OR (female breast neoplasm):ab,ti,kw OR (female breast tumor):ab,ti,kw OR (female breast tumour):ab,ti,kw OR (male breast neoplasm):ab,ti,kw OR (male breast tumor):ab,ti,kw OR (male breast tumour):ab,ti,kw OR (mamma tumor):ab,ti,kw OR (mamma tumour):ab,ti,kw OR (mammary gland neoplasia):ab,ti,kw OR (mammary gland neoplasm):ab,ti,kw OR (mammary gland tumor):ab,ti,kw OR (mammary gland tumorigenesis):ab,ti,kw OR (mammary gland tumour):ab,ti,kw OR (mammary neoplasia):ab,ti,kw OR (mammary neoplasm):ab,ti,kw OR (mammary neoplasms):ab,ti,kw OR (mammary tumor):ab,ti,kw OR (mammary tumor cell):ab,ti,kw OR (mammary tumorigenesis):ab,ti,kw OR (mammary tumour):ab,ti,kw OR (mammary tumour cell):ab,ti,kw OR (mass in the breast):ab,ti,kw OR (masses in the breast):ab,ti,kw OR (neoplasia of the breast):ab,ti,kw OR (neoplasm of the breast):ab,ti,kw OR (neoplasm of the mammary gland):ab,ti,kw OR (neoplastic breast):ab,ti,kw OR (neoplastic mammary):ab,ti,kw OR (neoplastic mammary gland):ab,ti,kw OR (tumor of the breast):ab,ti,kw OR (tumor of the female breast):ab,ti,kw OR (tumor of the male breast):ab,ti,kw OR (tumor of the mammary gland):ab,ti,kw OR (tumorigenesis of the breast):ab,ti,kw OR (tumorigenesis of the mammary gland):ab,ti,kw OR (tumour of the male breast):ab,ti,kw OR (unilateral breast neoplasm):ab,ti,kw OR (unilateral breast neoplasms):ab,ti,kw OR (unilateral breast tumor):ab,ti,kw OR (breast gland cancer):ab,ti,kw OR (breast gland neoplasm):ab,ti,kw OR (breast malignancies):ab,ti,kw OR (breast malignancy):ab,ti,kw OR (breast tumor malignant):ab,ti,kw OR (Ca breast):ab,ti,kw OR (cancer in the mammary gland):ab,ti,kw OR (cancer of the mammary gland):ab,ti,kw OR (cancer, breast):ab,ti,kw OR (malignancies of the breast):ab,ti,kw OR (malignancy of the breast):ab,ti,kw OR (malignant breast neoplasm):ab,ti,kw OR (malignant breast tumor):ab,ti,kw OR (malignant neoplasm of the breast):ab,ti,kw OR (malignant tumor of the breast):ab,ti,kw OR (mamma cancer):ab,ti,kw OR (mammary gland cancer):ab,ti,kw OR (mammary gland malignancy):ab,ti,kw OR (mammary malignancies):ab,ti,kw OR (mammary malignancy):ab,ti,kw OR (breast carcinomata):ab,ti,kw OR (breast carcinomatosis):ab,ti,kw OR (carcinoma in the mammary gland):ab,ti,kw OR (carcinoma of the breast):ab,ti,kw OR (carcinoma of the mamma):ab,ti,kw OR (carcinoma of the mammary gland):ab,ti,kw OR (carcinoma, mammary):ab,ti,kw OR (carcinomata of the breast):ab,ti,kw OR (carcinomatous breast):ab,ti,kw OR (carcinomatous mammary):ab,ti,kw OR (carcinomatous mammary gland):ab,ti,kw OR (human mammary carcinoma):ab,ti,kw OR (mamma carcinoma):ab,ti,kw OR (mammary carcinoma):ab,ti,kw OR (mammary carcinomata):ab,ti,kw OR (mammary carcinomatosis):ab,ti,kw OR (mammary gland carcinoma):ab,ti,kw OR (adenocarcinoma of the breast):ab,ti,kw OR (adenocarcinoma of the mamma):ab,ti,kw OR (adenocarcinoma of the mammary gland):ab,ti,kw OR (mammary adenocarcinoma):ab,ti,kw OR (breast adenocarcinoma):ab,ti,kw OR (Carcinoma, Ductal, Breast):ab,ti,kw OR (Carcinoma, Infiltrating Duct):ab,ti,kw OR (Carcinomas, Infiltrating Duct):ab,ti,kw OR (Carcinoma, Invasive Ductal, Breast):ab,ti,kw OR (Invasive Ductal Carcinoma, Breast):ab,ti,kw OR (Carcinoma, Mammary Ductal):ab,ti,kw OR (Carcinomas, Mammary Ductal):ab,ti,kw OR (Mammary Ductal Carcinomas):ab,ti,kw OR (Mammary Ductal Carcinoma):ab,ti,kw OR (breast duct carcinoma):ab,ti,kw OR (breast ductal adenocarcinoma):ab,ti,kw OR (carcinoma, ductal, breast):ab,ti,kw OR (ductal adenocarcinoma of the breast):ab,ti,kw OR (ductal breast adenocarcinoma):ab,ti,kw OR (ductal breast carcinoma):ab,ti,kw OR (ductal carcinoma of the breast):ab,ti,kw OR (ductal mammary carcinoma):ab,ti,kw OR (mammary duct carcinoma):ab,ti,kw OR (breast ductal carcinoma):ab,ti,kw OR (breast invasive ductal carcinoma):ab,ti,kw OR (ductal invasive breast carcinoma):ab,ti,kw OR (infiltrating duct carcinoma of breast):ab,ti,kw OR (infiltrating ductal breast cancer):ab,ti,kw OR (infiltrating ductal breast carcinoma):ab,ti,kw OR (infiltrating ductal carcinoma of the breast):ab,ti,kw OR (invasive ductal breast cancer):ab,ti,kw OR (invasive ductal carcinoma of the breast):ab,ti,kw OR (invasive ductal breast carcinoma):ab,ti,kw OR (Carcinoma, Lobular):ab,ti,kw OR (Carcinomas, Lobular):ab,ti,kw OR (Lobular Carcinoma):ab,ti,kw OR (Lobular Carcinomas):ab,ti,kw OR (carcinoma, lobular):ab,ti,kw OR (lobular adenocarcinoma):ab,ti,kw OR (lobular adenocarcinoma of the breast):ab,ti,kw OR (lobular breast adenocarcinoma):ab,ti,kw OR (lobular breast carcinoma):ab,ti,kw OR (lobular cancer):ab,ti,kw OR (lobular cancer of the breast):ab,ti,kw OR (lobular carcinoma of the breast):ab,ti,kw OR (lobular carcinoma):ab,ti,kw OR (infiltrating lobular breast cancer):ab,ti,kw OR (infiltrating lobular breast carcinoma):ab,ti,kw OR (infiltrating lobular carcinoma):ab,ti,kw OR (infiltrative lobular carcinoma):ab,ti,kw OR (invasive lobular breast cancer):ab,ti,kw OR (invasive lobular cancer of the breast):ab,ti,kw OR (invasive lobular carcinoma):ab,ti,kw OR (invasive lobular carcinoma of the breast):ab,ti,kw OR (invasive lobular breast carcinoma):ab,ti,kw

#3 MeSH descriptor: [Artificial Intelligence] explode all trees

#4 (computer assisted diagnosis):ab,ti,kw OR (automatic diagnosis):ab,ti,kw OR (computer diagnosis):ab,ti,kw OR (computer-assisted diagnosis):ab,ti,kw OR (computer-assisted image interpretation):ab,ti,kw OR (computer-assisted radiographic image interpretation):ab,ti,kw OR (diagnosis, computer):ab,ti,kw OR (diagnosis, computer-assisted):ab,ti,kw OR (image interpretation, computer-assisted):ab,ti,kw OR (radiographic image interpretation, computer-assisted):ab,ti,kw OR (Intelligence, Artificial):ab,ti,kw OR (Computer Reasoning):ab,ti,kw OR (Reasoning, Computer):ab,ti,kw OR (AI (Artificial Intelligence)):ab,ti,kw OR (Machine Intelligence):ab,ti,kw OR (Intelligence, Machine):ab,ti,kw OR (Computational Intelligence):ab,ti,kw OR (Intelligence, Computational):ab,ti,kw OR (Computer Vision Systems):ab,ti,kw OR (Computer Vision System):ab,ti,kw OR (System, Computer Vision):ab,ti,kw OR (Systems, Computer Vision):ab,ti,kw OR (Vision System, Computer):ab,ti,kw OR (Vision Systems, Computer):ab,ti,kw OR (artificial intelligence):ab,ti,kw OR (machine learning):ab,ti,kw OR (learning machine):ab,ti,kw OR (learning machines):ab,ti,kw OR (Learning, Machine):ab,ti,kw OR (Transfer Learning):ab,ti,kw OR (Learning, Transfer):ab,ti,kw OR (artificial neural network):ab,ti,kw OR (algorithmic neural network):ab,ti,kw OR (ANN):ab,ti,kw OR (ANN analysis):ab,ti,kw OR (ANN approach):ab,ti,kw OR (ANN method):ab,ti,kw OR (ANN methodology):ab,ti,kw OR (ANN methods):ab,ti,kw OR (ANN model):ab,ti,kw OR (ANN modeling):ab,ti,kw OR (ANN modelling):ab,ti,kw OR (ANN models):ab,ti,kw OR (ANN output):ab,ti,kw OR (ANN technique):ab,ti,kw OR (ANN techniques):ab,ti,kw OR (ANN training):ab,ti,kw OR (ANNs):ab,ti,kw OR (artificial neural networks):ab,ti,kw OR (artificial NN):ab,ti,kw OR (artificial NNs):ab,ti,kw OR (computational neural network):ab,ti,kw OR (computer neural network):ab,ti,kw OR (computer neural networks):ab,ti,kw OR (computerized neural network):ab,ti,kw OR (connectionist model):ab,ti,kw OR (connectionist network):ab,ti,kw OR (connectionist neural network):ab,ti,kw OR (connectionist system):ab,ti,kw OR (mathematical neural network):ab,ti,kw OR (neural network):ab,ti,kw OR (neural network algorithm):ab,ti,kw OR (neural network model):ab,ti,kw OR (neural networks ):ab,ti,kw OR (neural networks, computer):ab,ti,kw OR (deep learning):ab,ti,kw OR (deep machine learning):ab,ti,kw OR (deep ML):ab,ti,kw OR (hierarchical learning):ab,ti,kw OR (Learning, Deep):ab,ti,kw OR (Learning, Hierarchical):ab,ti,kw

#5 MeSH descriptor: [Radiology] explode all trees

#6 MeSH descriptor: [Diagnostic Imaging] explode all trees

#7 (imaging, diagnostic):ab,ti,kw OR (imaging, medical):ab,ti,kw OR (medical imaging):ab,ti,kw OR (ophthalmo diaphanoscopy):ab,ti,kw OR (diagnostic imaging):ab,ti,kw OR (imaging, magnetization transfer):ab,ti,kw OR (magnetic resonance imaging):ab,ti,kw OR (magnetic resonance tomography):ab,ti,kw OR (magnetization transfer imaging):ab,ti,kw OR (mr imaging):ab,ti,kw OR (MRI):ab,ti,kw OR (nuclear magnetic resonance imaging):ab,ti,kw OR (Imaging, Magnetic Resonance):ab,ti,kw OR (NMR Imaging):ab,ti,kw OR (Imaging, NMR):ab,ti,kw OR (Zeugmatography):ab,ti,kw OR (Tomography, MR):ab,ti,kw OR (Steady-State Free Precession MRI):ab,ti,kw OR (Steady State Free Precession MRI):ab,ti,kw OR (NMR Tomography):ab,ti,kw OR (Tomography, NMR):ab,ti,kw OR (MR Tomography):ab,ti,kw OR (Tomography, Proton Spin):ab,ti,kw OR (Proton Spin Tomography):ab,ti,kw OR (Magnetization Transfer Contrast Imaging):ab,ti,kw OR (fMRI):ab,ti,kw OR (Magnetic Resonance Imaging, Functional):ab,ti,kw OR (MRI, Functional):ab,ti,kw OR (Functional MRI):ab,ti,kw OR (Functional MRIs):ab,ti,kw OR (MRIs, Functional):ab,ti,kw OR (Functional Magnetic Resonance Imaging):ab,ti,kw OR (MRI Scans):ab,ti,kw OR (MRI Scan):ab,ti,kw OR (Scan, MRI):ab,ti,kw OR (Scans, MRI):ab,ti,kw OR (Imaging, Chemical Shift):ab,ti,kw OR (Chemical Shift Imagings):ab,ti,kw OR (Imagings, Chemical Shift):ab,ti,kw OR (Shift Imaging, Chemical):ab,ti,kw OR (Shift Imagings, Chemical):ab,ti,kw OR (Chemical Shift Imaging):ab,ti,kw OR (Spin Echo Imaging):ab,ti,kw OR (Echo Imaging, Spin):ab,ti,kw OR (Echo Imagings, Spin):ab,ti,kw OR (Imaging, Spin Echo):ab,ti,kw OR (Imagings, Spin Echo):ab,ti,kw OR (Spin Echo Imagings):ab,ti,kw OR (Magnetic Resonance Image):ab,ti,kw OR (Image, Magnetic Resonance):ab,ti,kw OR (Magnetic Resonance Images):ab,ti,kw OR (Resonance Image, Magnetic):ab,ti,kw OR (breast MRI):ab,ti,kw OR (magnetic resonance breast imaging):ab,ti,kw OR (magnetic resonance mammography):ab,ti,kw OR (breast magnetic resonance imaging):ab,ti,kw OR (Mammography):ab,ti,kw OR (mamilloscopy):ab,ti,kw OR (mammilloscopy):ab,ti,kw OR (mammo-graphy):ab,ti,kw OR (mammogram):ab,ti,kw OR (mastography Mammographies):ab,ti,kw OR (Digital Breast Tomosynthesis):ab,ti,kw OR (Breast Tomosyntheses, Digital):ab,ti,kw OR (Breast Tomosynthesis, Digital):ab,ti,kw OR (Digital Breast Tomosyntheses):ab,ti,kw OR (X-ray Breast Tomosynthesis):ab,ti,kw OR (Breast Tomosyntheses, X-ray):ab,ti,kw OR (Breast Tomosynthesis, X-ray):ab,ti,kw OR (X-ray Breast Tomosyntheses):ab,ti,kw OR (X ray Breast Tomosynthesis):ab,ti,kw OR (3D-Mammography):ab,ti,kw OR (3D-Mammographies):ab,ti,kw OR (3D Mammography):ab,ti,kw OR (Digital Mammography):ab,ti,kw OR (Digital Mammographies):ab,ti,kw OR (Mammographies, Digital):ab,ti,kw OR (Mammography, Digital):ab,ti,kw OR (diagnostic radiology):ab,ti,kw OR (radio diagnosis):ab,ti,kw OR (radiographic examination):ab,ti,kw OR (radiologic diagnosis):ab,ti,kw OR (radiologic examination):ab,ti,kw OR (radiological diagnosis):ab,ti,kw OR (radiological examination):ab,ti,kw OR (radiology, diagnostic):ab,ti,kw OR (roentgen diagnosis):ab,ti,kw OR (roentgen diagnostics):ab,ti,kw OR (roentgen examination):ab,ti,kw OR (roentgen screening):ab,ti,kw OR (roentgenologic diagnosis):ab,ti,kw OR (roentgenologic diagnostics):ab,ti,kw OR (roentgenologic examination):ab,ti,kw OR (roentgenologic screening):ab,ti,kw OR (roentgenological diagnosis):ab,ti,kw OR (roentgenological diagnostics):ab,ti,kw OR (roentgenological examination):ab,ti,kw OR (roentgenological screening):ab,ti,kw OR (rontgen diagnosis):ab,ti,kw OR (rontgen examination):ab,ti,kw OR (rontgenologic diagnosis):ab,ti,kw OR (rontgenologic examination):ab,ti,kw OR (rontgenological diagnosis):ab,ti,kw OR (rontgenological examination):ab,ti,kw OR (X ray diagnosis):ab,ti,kw OR (X ray examination):ab,ti,kw OR (radiodiagnosis):ab,ti,kw OR (radiologic*):ab,ti,kw OR (roentgenological*):ab,ti,kw OR (roentgenology):ab,ti,kw OR (radiology):ab,ti,kw OR (Radiography):ab,ti,kw OR (Diagnostic X-Ray):ab,ti,kw OR (Diagnostic X Ray):ab,ti,kw OR (Diagnostic X-Rays):ab,ti,kw OR (X-Rays, Diagnostic):ab,ti,kw OR (X-Ray, Diagnostic):ab,ti,kw OR (X Ray, Diagnostic):ab,ti,kw OR (Diagnostic X-Ray Radiology):ab,ti,kw OR (Diagnostic X Ray Radiology):ab,ti,kw OR (X-Ray Radiology, Diagnostic):ab,ti,kw OR (X Ray Radiology, Diagnostic):ab,ti,kw OR (Radiology, Diagnostic X-Ray):ab,ti,kw OR (Radiology, Diagnostic X Ray):ab,ti,kw OR (dual-energy scanned projection radiography):ab,ti,kw OR (electroradiography):ab,ti,kw OR (pneumoradiography):ab,ti,kw OR (radiogram):ab,ti,kw OR (radiographic method):ab,ti,kw OR (radiography, dual-energy scanned projection):ab,ti,kw OR (radioimaging):ab,ti,kw OR (radiophotography):ab,ti,kw OR (roentgen photography):ab,ti,kw OR (roentgenography):ab,ti,kw OR (roentgenoscopy):ab,ti,kw OR (rontgenography):ab,ti,kw OR (X ray imaging):ab,ti,kw OR (x ray photography ):ab,ti,kw OR (computer assisted positron emission tomography):ab,ti,kw OR (positron emission computed tomography):ab,ti,kw OR (positron emission tomography computed tomography):ab,ti,kw OR (positron emission tomography, computer assisted):ab,ti,kw OR (positron-emission tomography and computed tomography):ab,ti,kw OR (positron emission tomography-computed tomography):ab,ti,kw OR (PET-CT Scan):ab,ti,kw OR (PET-CT Scans):ab,ti,kw OR (Scan, PET-CT):ab,ti,kw OR (Scans, PET-CT):ab,ti,kw OR (PET CT Scan):ab,ti,kw OR (CT Scan, PET):ab,ti,kw OR (CT Scans, PET):ab,ti,kw OR (PET CT Scans):ab,ti,kw OR (Scan, PET CT):ab,ti,kw OR (Scans, PET CT):ab,ti,kw OR (PET-CT):ab,ti,kw OR (CT PET):ab,ti,kw OR (CT PET Scan):ab,ti,kw OR (CT PET Scans):ab,ti,kw OR (PET Scan, CT):ab,ti,kw OR (PET Scans, CT):ab,ti,kw OR (Scan, CT PET):ab,ti,kw OR (Scans, CT PET):ab,ti,kw OR (p.e.t.):ab,ti,kw OR (PET scanning):ab,ti,kw OR (positron emission tomographic scan):ab,ti,kw OR (positron emission tomographic scanning):ab,ti,kw OR (positron tomography):ab,ti,kw OR (positron-emission tomography):ab,ti,kw OR (tomography, positron):ab,ti,kw OR (positron emission tomography):ab,ti,kw OR (PET Imaging):ab,ti,kw OR (Imaging, PET):ab,ti,kw OR (PET Imagings):ab,ti,kw OR (Positron-Emission Tomography Imaging):ab,ti,kw OR (Imaging, Positron-Emission Tomography):ab,ti,kw OR (Positron Emission Tomography Imaging):ab,ti,kw OR (Positron-Emission Tomography Imagings):ab,ti,kw OR (Tomography Imaging, Positron-Emission):ab,ti,kw OR (PET Scan):ab,ti,kw OR (PET Scans):ab,ti,kw OR (Scan, PET):ab,ti,kw OR (Tomography, Positron-Emission):ab,ti,kw OR (Tomography, Positron Emission):ab,ti,kw OR (diagnostic ultrasonic examination):ab,ti,kw OR (diagnostic ultrasonic imaging):ab,ti,kw OR (diagnostic ultrasonic method):ab,ti,kw OR (doptone):ab,ti,kw OR (duplex echography):ab,ti,kw OR (echogram):ab,ti,kw OR (echographic evaluation):ab,ti,kw OR (echoscopy):ab,ti,kw OR (echosound):ab,ti,kw OR (high resolution echography):ab,ti,kw OR (scanning, ultrasonic):ab,ti,kw OR (sonogram):ab,ti,kw OR (sonographic examination):ab,ti,kw OR (sonographic screening):ab,ti,kw OR (sonography):ab,ti,kw OR (ultrasonic detection):ab,ti,kw OR (ultrasonic echo):ab,ti,kw OR (ultrasonic examination):ab,ti,kw OR (ultrasonic scanning):ab,ti,kw OR (ultrasonic scintillation):ab,ti,kw OR (ultrasonogram):ab,ti,kw OR (ultrasonographic examination):ab,ti,kw OR (ultrasonographic screening):ab,ti,kw OR (ultrasonography):ab,ti,kw OR (ultrasound diagnosis):ab,ti,kw OR (ultrasound scanning ):ab,ti,kw OR (Echotomography):ab,ti,kw OR (Ultrasonic Imaging):ab,ti,kw OR (Imaging, Ultrasonic):ab,ti,kw OR (Sonography, Medical):ab,ti,kw OR (Medical Sonography):ab,ti,kw OR (Ultrasound Imaging):ab,ti,kw OR (Imagings, Ultrasound):ab,ti,kw OR (Imaging, Ultrasound):ab,ti,kw OR (Ultrasonographic Imaging):ab,ti,kw OR (Imagings, Ultrasonographic):ab,ti,kw OR (Imaging, Ultrasonographic):ab,ti,kw OR (Ultrasonographic Imagings):ab,ti,kw OR (Echography):ab,ti,kw OR (Diagnostic Ultrasound):ab,ti,kw OR (Diagnostic Ultrasounds):ab,ti,kw OR (Ultrasound, Diagnostic):ab,ti,kw OR (Ultrasounds, Diagnostic):ab,ti,kw OR (Echotomography, Computer):ab,ti,kw OR (Computer Echotomography):ab,ti,kw OR (Tomography, Ultrasonic):ab,ti,kw OR (Ultrasonic Tomography):ab,ti,kw OR (Diagnosis, Ultrasonic):ab,ti,kw OR (Diagnoses, Ultrasonic):ab,ti,kw OR (Ultrasonic Diagnoses):ab,ti,kw OR (Ultrasonic Diagnosis):ab,ti,kw OR (Echomammography):ab,ti,kw OR (breast echography):ab,ti,kw OR (breast ultrasound):ab,ti,kw OR (echography, breast):ab,ti,kw OR (sonography, breast):ab,ti,kw OR (Ultrasonography, Mammary):ab,ti,kw OR (Ultrasonic Mammography):ab,ti,kw OR (Mammographies, Ultrasonic):ab,ti,kw OR (Ultrasonic Mammographies):ab,ti,kw OR (Mammography, Ultrasonic):ab,ti,kw OR (Mammography, Ultrasound):ab,ti,kw OR (Mammographies, Ultrasound):ab,ti,kw OR (Ultrasound Mammographies):ab,ti,kw OR (Ultrasound Mammography):ab,ti,kw OR (Mammary Ultrasonography):ab,ti,kw OR (Mammary Ultrasonographies):ab,ti,kw OR (Ultrasonographies, Mammary):ab,ti,kw OR (Ultrasonography, Breast):ab,ti,kw OR (Breast Ultrasonography):ab,ti,kw OR (Breast Ultrasonographies):ab,ti,kw OR (Ultrasonographies, Breast):ab,ti,kw

#8 MeSH descriptor: [Image Processing, Computer-Assisted] explode all trees

#9 (computer-assisted image processing):ab,ti,kw OR (processing, image):ab,ti,kw OR (image processing):ab,ti,kw OR (Image Processing, Computer Assisted):ab,ti,kw OR (Computer-Assisted Image Processing):ab,ti,kw OR (Computer Assisted Image Processing):ab,ti,kw OR (Image Analysis, Computer-Assisted):ab,ti,kw OR (Computer-Assisted Image Analyses):ab,ti,kw OR (Image Analyses, Computer-Assisted):ab,ti,kw OR (Image Analysis, Computer Assisted):ab,ti,kw OR (Analysis, Computer-Assisted Image):ab,ti,kw OR (Computer-Assisted Image Analysis):ab,ti,kw OR (Computer Assisted Image Analysis):ab,ti,kw OR (Image Reconstruction):ab,ti,kw OR (Image Reconstructions):ab,ti,kw OR (Reconstruction, Image):ab,ti,kw OR (Reconstructions, Image):ab,ti,kw OR (Digital Image Processing):ab,ti,kw OR (Image Processing, Digital):ab,ti,kw OR (Processing, Digital Image):ab,ti,kw OR (Processings, Digital Image):ab,ti,kw OR (Medical Image Processing):ab,ti,kw OR (Image Processing, Medical):ab,ti,kw OR (Image Processings, Medical):ab,ti,kw OR (Medical Image Processings):ab,ti,kw OR (Processing, Medical Image):ab,ti,kw OR (Processings, Medical Image):ab,ti,kw OR (Biomedical Image Processing):ab,ti,kw OR (Image Processing, Biomedical):ab,ti,kw OR (Processing, Biomedical Image):ab,ti,kw OR (Image Interpretation, Computer-Assisted):ab,ti,kw OR (Computer-Assisted Image Interpretation):ab,ti,kw OR (Computer-Assisted Image Interpretations):ab,ti,kw OR (Image Interpretations, Computer-Assisted):ab,ti,kw OR (Interpretation, Computer-Assisted Image):ab,ti,kw OR (Interpretations, Computer-Assisted Image):ab,ti,kw OR (Image Interpretation, Computer Assisted):ab,ti,kw OR (computer assisted diagnosis):ab,ti,kw OR (automatic diagnosis):ab,ti,kw OR (computer diagnosis):ab,ti,kw OR (computer-assisted diagnosis):ab,ti,kw OR (computer-assisted radiographic image interpretation):ab,ti,kw OR (diagnosis, computer):ab,ti,kw OR (diagnosis, computer-assisted):ab,ti,kw OR (radiographic image interpretation, computer-assisted):ab,ti,kw OR (computer assisted diagnosis):ab,ti,kw OR (Radiomi*):ab,ti,kw

#10 MeSH descriptor: [DNA Methylation] explode all trees

#11 MeSH descriptor: [Gene Expression] explode all trees

#12 MeSH descriptor: [Gene Expression Regulation, Neoplastic] explode all trees

#13 MeSH descriptor: [Gene Ontology] explode all trees

#14 MeSH descriptor: [Genetic Code] explode all trees

#15 MeSH descriptor: [RNA, Messenger] explode all trees

#16 MeSH descriptor: [Sequence Analysis, DNA] explode all trees

#17 MeSH descriptor: [Sequence Analysis, Protein] explode all trees

#18 MeSH descriptor: [Sequence Analysis, RNA] explode all trees

#19 MeSH descriptor: [Transcriptome] explode all trees

#20 MeSH descriptor: [Whole Genome Sequencing] explode all trees

#21 MeSH descriptor: [DNA Mutational Analysis] explode all trees

#22 (Comparative Genomics):ab,ti,kw OR (Genomics, Comparative):ab,ti,kw OR (Structural Genomics):ab,ti,kw OR (Genomics, Structural):ab,ti,kw OR (Functional Genomics):ab,ti,kw OR (Genomics, Functional):ab,ti,kw OR (genome components):ab,ti,kw OR (genome, helminth):ab,ti,kw OR (genome, protozoan):ab,ti,kw OR (genome, protozoon):ab,ti,kw OR (protozoan genome):ab,ti,kw OR (protozoon genome):ab,ti,kw OR (Genome*):ab,ti,kw OR (expression*, gene):ab,ti,kw OR (genetic expression):ab,ti,kw OR (genome expression):ab,ti,kw OR (gene expression*):ab,ti,kw OR (dosage compensation):ab,ti,kw OR (dosage compensation, genetic):ab,ti,kw OR (gene dosage compensation):ab,ti,kw OR (gene expression regulation, archaeal):ab,ti,kw OR (gene expression regulation, bacterial):ab,ti,kw OR (gene expression regulation, developmental):ab,ti,kw OR (gene expression regulation, enzymologic):ab,ti,kw OR (gene expression regulation, fungal):ab,ti,kw OR (gene expression regulation, leukaemic):ab,ti,kw OR (gene expression regulation, leukemic):ab,ti,kw OR (gene expression regulation, neoplastic):ab,ti,kw OR (gene expression regulation, plant):ab,ti,kw OR (gene expression regulation, viral):ab,ti,kw OR (genetic dosage compensation):ab,ti,kw OR (Regulation, Gene Action):ab,ti,kw OR (Gene Action Regulation):ab,ti,kw OR (Expression Regulation, Gene):ab,ti,kw OR (Regulation, Gene Expression):ab,ti,kw OR (Regulation of Gene Expression):ab,ti,kw OR (gene expression regulation):ab,ti,kw OR (epigenomic*):ab,ti,kw OR (Epigenetic*):ab,ti,kw OR (Gene Ontologies):ab,ti,kw OR (Ontologies, Gene):ab,ti,kw OR (Ontology, Gene):ab,ti,kw OR (Gene Ontology Project):ab,ti,kw OR (Gene Ontology Projects):ab,ti,kw OR (Ontology Project, Gene):ab,ti,kw OR (Ontology Projects, Gene):ab,ti,kw OR (Project, Gene Ontology):ab,ti,kw OR (Projects, Gene Ontology):ab,ti,kw OR (gene ontology):ab,ti,kw OR (gene expression analysis):ab,ti,kw OR (gene expression profile):ab,ti,kw OR (gene product profiling):ab,ti,kw OR (gene expression profiling*):ab,ti,kw OR (Profiling, Gene Expression):ab,ti,kw OR (Profilings, Gene Expression):ab,ti,kw OR (Gene Expression Monitoring):ab,ti,kw OR (Gene Expression Monitorings):ab,ti,kw OR (Monitoring, Gene Expression):ab,ti,kw OR (Monitorings, Gene Expression):ab,ti,kw OR (Gene Expression Pattern Analysis):ab,ti,kw OR (Transcript Expression Analysis):ab,ti,kw OR (Analyses, Transcript Expression):ab,ti,kw OR (Analysis, Transcript Expression):ab,ti,kw OR (Transcript Expression Analyses):ab,ti,kw OR (Transcriptome Analysis):ab,ti,kw OR (Analyses, Transcriptome):ab,ti,kw OR (Analysis, Transcriptome):ab,ti,kw OR (Transcriptome Analyses):ab,ti,kw OR (Transcriptome Profiling):ab,ti,kw OR (Profilings, Transcriptome):ab,ti,kw OR (Profiling, Transcriptome):ab,ti,kw OR (Transcriptome Profilings):ab,ti,kw OR (Transcriptomics):ab,ti,kw OR (Gene Expression Analysis):ab,ti,kw OR (Analyses, Gene Expression):ab,ti,kw OR (Analysis, Gene Expression):ab,ti,kw OR (Expression Analyses, Gene):ab,ti,kw OR (Expression Analysis, Gene):ab,ti,kw OR (Gene Expression Analyses):ab,ti,kw OR (mRNA Differential Display):ab,ti,kw OR (Differential Display, mRNA):ab,ti,kw OR (Differential Displays, mRNA):ab,ti,kw OR (mRNA Differential Displays):ab,ti,kw OR (code, genetic):ab,ti,kw OR (deoxyribonucleic acid code):ab,ti,kw OR (dna code):ab,ti,kw OR (genetic coding):ab,ti,kw OR (genetic code*):ab,ti,kw OR (codes, genetic):ab,ti,kw OR (transcriptome*):ab,ti,kw OR (Gene Expression Profiles):ab,ti,kw OR (Expression Profile, Gene):ab,ti,kw OR (Expression Profiles, Gene):ab,ti,kw OR (Gene Expression Profile):ab,ti,kw OR (Profile, Gene Expression):ab,ti,kw OR (Profiles, Gene Expression):ab,ti,kw OR (Gene Expression Signatures):ab,ti,kw OR (Expression Signature, Gene):ab,ti,kw OR (Expression Signatures, Gene):ab,ti,kw OR (Gene Expression Signature):ab,ti,kw OR (Signature, Gene Expression):ab,ti,kw OR (Signatures, Gene Expression):ab,ti,kw OR (Transcriptome Profiles):ab,ti,kw OR (Profiles, Transcriptome):ab,ti,kw OR (Profile, Transcriptome):ab,ti,kw OR (Transcriptome Profile):ab,ti,kw OR (b rna):ab,ti,kw OR (brna):ab,ti,kw OR (messenger ribonucleic acid):ab,ti,kw OR (ribonucleic acid, messenger):ab,ti,kw OR (rna, messenger):ab,ti,kw OR (RNA, messenger, stored):ab,ti,kw OR (rna, messenger):ab,ti,kw OR (Messenger RNA):ab,ti,kw OR (mRNA):ab,ti,kw OR (mRNA, Non-Polyadenylated):ab,ti,kw OR (mRNA, Non Polyadenylated):ab,ti,kw OR (Non-Polyadenylated mRNA):ab,ti,kw OR (Non Polyadenylated mRNA):ab,ti,kw OR (mRNA, Polyadenylated):ab,ti,kw OR (Polyadenylated mRNA):ab,ti,kw OR (Messenger RNA, Polyadenylated):ab,ti,kw OR (Polyadenylated Messenger RNA):ab,ti,kw OR (RNA, Polyadenylated Messenger):ab,ti,kw OR (RNA, Messenger, Polyadenylated):ab,ti,kw OR (RNA, Polyadenylated):ab,ti,kw OR (Polyadenylated RNA):ab,ti,kw OR (RNA seq):ab,ti,kw OR (RNA-Seq):ab,ti,kw OR (RNAseq):ab,ti,kw OR (sequence analysis, RNA):ab,ti,kw OR (Analyses, RNA Sequence):ab,ti,kw OR (RNA Sequence Analyses):ab,ti,kw OR (Sequence Analyses, RNA):ab,ti,kw OR (Analysis, RNA Sequence):ab,ti,kw OR (RNA Sequence Analysis):ab,ti,kw OR (Sequence Determinations, RNA):ab,ti,kw OR (Determination, RNA Sequence):ab,ti,kw OR (Determinations, RNA Sequence):ab,ti,kw OR (RNA Sequence Determination):ab,ti,kw OR (RNA Sequence Determinations):ab,ti,kw OR (RNA Sequencing):ab,ti,kw OR (Sequencing, RNA):ab,ti,kw OR (Sequence Determination, RNA):ab,ti,kw OR (amino acid sequence analysis):ab,ti,kw OR (amino acid sequencing):ab,ti,kw OR (aminoacid sequence analysis):ab,ti,kw OR (genetic sequencing):ab,ti,kw OR (genomic sequencing):ab,ti,kw OR (macromolecular sequencing):ab,ti,kw OR (molecular sequencing):ab,ti,kw OR (peptide sequence analysis):ab,ti,kw OR (peptide sequencing):ab,ti,kw OR (protein sequence analysis):ab,ti,kw OR (protein sequencing):ab,ti,kw OR (sequence analysis, protein):ab,ti,kw OR (Analysis, Sequence):ab,ti,kw OR (Analyses, Sequence):ab,ti,kw OR (Sequence Analyses):ab,ti,kw OR (Sequence Determinations):ab,ti,kw OR (Determination, Sequence):ab,ti,kw OR (Determinations, Sequence):ab,ti,kw OR (Sequence Determination):ab,ti,kw OR (sequence analysis):ab,ti,kw OR (DNA sequence determination):ab,ti,kw OR (DNA sequencing):ab,ti,kw OR (Analyses, DNA Sequence):ab,ti,kw OR (DNA Sequence Analyses):ab,ti,kw OR (Sequence Analys*, DNA):ab,ti,kw OR (Analysis, DNA Sequence):ab,ti,kw OR (DNA Sequence Analysis):ab,ti,kw OR (Sequence Determinations, DNA):ab,ti,kw OR (Determination, DNA Sequence):ab,ti,kw OR (Determinations, DNA Sequence):ab,ti,kw OR (DNA Sequence Determination):ab,ti,kw OR (DNA Sequence Determinations):ab,ti,kw OR (DNA Sequencing):ab,ti,kw OR (Sequencing, DNA):ab,ti,kw OR (Sequence Determination, DNA):ab,ti,kw OR (entire genome sequencing):ab,ti,kw OR (full genome sequencing):ab,ti,kw OR (WGS):ab,ti,kw OR (WGS analysis):ab,ti,kw OR (Genome Sequencing, Whole):ab,ti,kw OR (Sequencing, Whole Genome):ab,ti,kw OR (Complete Genome Sequencing):ab,ti,kw OR (Genome Sequencing, Complete):ab,ti,kw OR (Sequencing, Complete Genome):ab,ti,kw OR (whole genome sequencing):ab,ti,kw OR (prote-omics):ab,ti,kw OR (protein omics):ab,ti,kw OR (proteinomics):ab,ti,kw OR (Proteomics):ab,ti,kw OR (Peptidomics):ab,ti,kw OR (deoxyribonucleic acid methylation):ab,ti,kw OR (DNA hypermethylation):ab,ti,kw OR (DNA hypomethylation):ab,ti,kw OR (methylated deoxyribonucleic acid):ab,ti,kw OR (methylated dna):ab,ti,kw OR (DNA methylation*):ab,ti,kw OR (Methylation, DNA):ab,ti,kw OR (Methylations, DNA):ab,ti,kw OR (Analysis, DNA Mutational):ab,ti,kw OR (Analyses, DNA Mutational):ab,ti,kw OR (DNA Mutational Analyses):ab,ti,kw OR (Mutational Analyses, DNA):ab,ti,kw OR (Mutational Analysis, DNA):ab,ti,kw OR (community genomic):ab,ti,kw OR (community genomics):ab,ti,kw OR (eco-genomics):ab,ti,kw OR (ecogenomic):ab,ti,kw OR (ecogenomics):ab,ti,kw OR (ecological genomic):ab,ti,kw OR (ecological genomics):ab,ti,kw OR (environmental genomic):ab,ti,kw OR (environmental genomics):ab,ti,kw OR (meta-genomics):ab,ti,kw OR (Metagenomic):ab,ti,kw OR (Metagenomics):ab,ti,kw OR (Genomics, Environmental):ab,ti,kw OR (Genomics, Community):ab,ti,kw OR (Population Genomics):ab,ti,kw OR (Genomics, Population):ab,ti

#23 MeSH descriptor: [Imaging Genomics] explode all trees

#24 MeSH descriptor: [Radiation Genomics] explode all trees

#25 MeSH descriptor: [Radiobiology] explode all trees

#26 MeSH descriptor: [Radiation Oncology] explode all trees

#27 (imaging radiogenomics):ab,ti,kw OR (neuroimaging genomics):ab,ti,kw OR (imaging genomics):ab,ti,kw OR (Genomics, Imaging):ab,ti,kw OR (Radiogenomic* ):ab,ti,kw OR (radiation genomics):ab,ti,kw OR (radio-genomics):ab,ti,kw OR (Oncology, Radiation):ab,ti,kw OR (Therapeutic Radiology):ab,ti,kw OR (Radiology, Therapeutic):ab,ti,kw OR (radiation oncology):ab,ti,kw OR (Biology, Radiation):ab,ti,kw OR (Radiation Biology):ab,ti,kw OR (radio biology):ab,ti,kw OR (radiobiology):ab,ti,kw

#28 #1 OR #2

#29 #3 OR #4

#30 #5 OR #6 OR #7

#31 #8 OR #9

#32 #10 OR #11 OR #12 OR #13 OR #14 OR #15 OR #16 OR #17 OR #18 OR #19 OR #20 OR #21 OR #22

#33 #23 OR #24 OR #25 OR #26 OR #27

#34 #29 AND #30

#35 #34 OR #31

#36 #35 AND #32

#37 #36 OR #33

#38 #37 AND #28

1. **Web of Science Core Collection search strategy (n = 7503)**
2. **TS=(Breast Neoplasm OR Breast Neoplasms OR Neoplasm, Breast OR Neoplasms, Breast OR Breast Tumors OR Breast Tumor OR Tumor, Breast OR Tumors, Breast OR Breast Cancer OR Cancer, Breast OR Cancer of Breast OR Cancer of the Breast OR Malignant Neoplasm of Breast OR Breast Malignant Neoplasm OR Breast Malignant Neoplasms OR Malignant Tumor of Breast OR Breast Malignant Tumor OR Breast Malignant Tumors OR Mammary Cancer OR Cancer, Mammary OR Cancers, Mammary OR Mammary Cancers OR Mammary Neoplasms, Human OR Human Mammary Neoplasm OR Human Mammary Neoplasms OR Neoplasm, Human Mammary OR Neoplasms, Human Mammary OR Mammary Neoplasm, Human OR Breast Carcinoma OR Breast Carcinomas OR Carcinoma, Breast OR Carcinomas, Breast OR Mammary Carcinoma, Human OR Carcinoma, Human Mammary OR Carcinomas, Human Mammary OR Human Mammary Carcinomas OR Mammary Carcinomas, Human OR Human Mammary Carcinoma OR bilateral breast neoplasm OR bilateral breast tumor OR bilateral breast tumour OR breast gland tumor OR breast gland tumour OR breast mass OR breast neoplasia OR breast neoplasm OR breast neoplasms OR breast neoplasms, male OR breast tumorigenesis OR breast tumour OR female breast neoplasm OR female breast tumor OR female breast tumour OR male breast neoplasm OR male breast tumor OR male breast tumour OR mamma tumor OR mamma tumour OR mammary gland neoplasia OR mammary gland neoplasm OR mammary gland tumor OR mammary gland tumorigenesis OR mammary gland tumour OR mammary neoplasia OR mammary neoplasm OR mammary neoplasms OR mammary tumor OR mammary tumor cell OR mammary tumorigenesis OR mammary tumour OR mammary tumour cell OR mass in the breast OR masses in the breast OR neoplasia of the breast OR neoplasm of the breast OR neoplasm of the mammary gland OR neoplastic breast OR neoplastic mammary OR neoplastic mammary gland OR tumor of the breast OR tumor of the female breast OR tumor of the male breast OR tumor of the mammary gland OR tumorigenesis of the breast OR tumorigenesis of the mammary gland OR tumour of the male breast OR unilateral breast neoplasm OR unilateral breast neoplasms OR unilateral breast tumor OR breast gland cancer OR breast gland neoplasm OR breast malignancies OR breast malignancy OR breast tumor malignant OR Ca breast OR cancer in the mammary gland OR cancer of the mammary gland OR cancer, breast OR malignancies of the breast OR malignancy of the breast OR malignant breast neoplasm OR malignant breast tumor OR malignant neoplasm of the breast OR malignant tumor of the breast OR mamma cancer OR mammary gland cancer OR mammary gland malignancy OR mammary malignancies OR mammary malignancy OR breast carcinomata OR breast carcinomatosis OR carcinoma in the mammary gland OR carcinoma of the breast OR carcinoma of the mamma OR carcinoma of the mammary gland OR carcinoma, mammary OR carcinomata of the breast OR carcinomatous breast OR carcinomatous mammary OR carcinomatous mammary gland OR human mammary carcinoma OR mamma carcinoma OR mammary carcinoma OR mammary carcinomata OR mammary carcinomatosis OR mammary gland carcinoma OR adenocarcinoma of the breast OR adenocarcinoma of the mamma OR adenocarcinoma of the mammary gland OR mammary adenocarcinoma OR breast adenocarcinoma OR Carcinoma, Ductal, Breast OR Carcinoma, Infiltrating Duct OR Carcinomas, Infiltrating Duct OR Carcinoma, Invasive Ductal, Breast OR Invasive Ductal Carcinoma, Breast OR Carcinoma, Mammary Ductal OR Carcinomas, Mammary Ductal OR Mammary Ductal Carcinomas OR Mammary Ductal Carcinoma OR breast duct carcinoma OR breast ductal adenocarcinoma OR carcinoma, ductal, breast OR ductal adenocarcinoma of the breast OR ductal breast adenocarcinoma OR ductal breast carcinoma OR ductal carcinoma of the breast OR ductal mammary carcinoma OR mammary duct carcinoma OR breast ductal carcinoma OR breast invasive ductal carcinoma OR ductal invasive breast carcinoma OR infiltrating duct carcinoma of breast OR infiltrating ductal breast cancer OR infiltrating ductal breast carcinoma OR infiltrating ductal carcinoma of the breast OR invasive ductal breast cancer OR invasive ductal carcinoma of the breast OR invasive ductal breast carcinoma OR Carcinoma, Lobular OR Carcinomas, Lobular OR Lobular Carcinoma OR Lobular Carcinomas OR carcinoma, lobular OR lobular adenocarcinoma OR lobular adenocarcinoma of the breast OR lobular breast adenocarcinoma OR lobular breast carcinoma OR lobular cancer OR lobular cancer of the breast OR lobular carcinoma of the breast OR lobular carcinoma OR infiltrating lobular breast cancer OR infiltrating lobular breast carcinoma OR infiltrating lobular carcinoma OR infiltrative lobular carcinoma OR invasive lobular breast cancer OR invasive lobular cancer of the breast OR invasive lobular carcinoma OR invasive lobular carcinoma of the breast OR invasive lobular breast carcinoma)**
3. **TS=(computer assisted diagnosis OR automatic diagnosis OR computer diagnosis OR computer-assisted diagnosis OR computer-assisted image interpretation OR computer-assisted radiographic image interpretation OR diagnosis, computer OR diagnosis, computer-assisted OR image interpretation, computer-assisted OR radiographic image interpretation, computer-assisted OR Intelligence, Artificial OR Computer Reasoning OR Reasoning, Computer OR AI (Artificial Intelligence) OR Machine Intelligence OR Intelligence, Machine OR Computational Intelligence OR Intelligence, Computational OR Computer Vision Systems OR Computer Vision System OR System, Computer Vision OR Systems, Computer Vision OR Vision System, Computer OR Vision Systems, Computer OR artificial intelligence OR machine learning OR learning machine OR learning machines OR Learning, Machine OR Transfer Learning OR Learning, Transfer OR artificial neural network OR algorithmic neural network OR ANN OR ANN analysis OR ANN approach OR ANN method OR ANN methodology OR ANN methods OR ANN model OR ANN modeling OR ANN modelling OR ANN models OR ANN output OR ANN technique OR ANN techniques OR ANN training OR ANNs OR artificial neural networks OR artificial NN OR artificial NNs OR computational neural network OR computer neural network OR computer neural networks OR computerized neural network OR connectionist model OR connectionist network OR connectionist neural network OR connectionist system OR mathematical neural network OR neural network OR neural network algorithm OR neural network model OR neural networks OR neural networks, computer OR deep learning OR deep machine learning OR deep ML OR hierarchical learning OR Learning, Deep OR Learning, Hierarchical)**
4. **TS=(imaging, diagnostic OR imaging, medical OR medical imaging OR ophthalmo diaphanoscopy OR diagnostic imaging OR imaging, magnetization transfer OR magnetic resonance imaging OR magnetic resonance tomography OR magnetization transfer imaging OR mr imaging OR MRI OR nuclear magnetic resonance imaging OR Imaging, Magnetic Resonance OR NMR Imaging OR Imaging, NMR OR Zeugmatography OR Tomography, MR OR Steady-State Free Precession MRI OR Steady State Free Precession MRI OR NMR Tomography OR Tomography, NMR OR MR Tomography OR Tomography, Proton Spin OR Proton Spin Tomography OR Magnetization Transfer Contrast Imaging OR fMRI OR Magnetic Resonance Imaging, Functional OR MRI, Functional OR Functional MRI OR Functional MRIs OR MRIs, Functional OR Functional Magnetic Resonance Imaging OR MRI Scans OR MRI Scan OR Scan, MRI OR Scans, MRI OR Imaging, Chemical Shift OR Chemical Shift Imagings OR Imagings, Chemical Shift OR Shift Imaging, Chemical OR Shift Imagings, Chemical OR Chemical Shift Imaging OR Spin Echo Imaging OR Echo Imaging, Spin OR Echo Imagings, Spin OR Imaging, Spin Echo OR Imagings, Spin Echo OR Spin Echo Imagings OR Magnetic Resonance Image OR Image, Magnetic Resonance OR Magnetic Resonance Images OR Resonance Image, Magnetic OR breast MRI OR magnetic resonance breast imaging OR magnetic resonance mammography OR breast magnetic resonance imaging OR Mammography OR mamilloscopy OR mammilloscopy OR mammo-graphy OR mammogram OR mastography Mammographies OR Digital Breast Tomosynthesis OR Breast Tomosyntheses, Digital OR Breast Tomosynthesis, Digital OR Digital Breast Tomosyntheses OR X-ray Breast Tomosynthesis OR Breast Tomosyntheses, X-ray OR Breast Tomosynthesis, X-ray OR X-ray Breast Tomosyntheses OR X ray Breast Tomosynthesis OR 3D-Mammography OR 3D-Mammographies OR 3D Mammography OR Digital Mammography OR Digital Mammographies OR Mammographies, Digital OR Mammography, Digital OR diagnostic radiology OR radio diagnosis OR radiographic examination OR radiologic diagnosis OR radiologic examination OR radiological diagnosis OR radiological examination OR radiology, diagnostic OR roentgen diagnosis OR roentgen diagnostics OR roentgen examination OR roentgen screening OR roentgenologic diagnosis OR roentgenologic diagnostics OR roentgenologic examination OR roentgenologic screening OR roentgenological diagnosis OR roentgenological diagnostics OR roentgenological examination OR roentgenological screening OR rontgen diagnosis OR rontgen examination OR rontgenologic diagnosis OR rontgenologic examination OR rontgenological diagnosis OR rontgenological examination OR X ray diagnosis OR X ray examination OR radiodiagnosis OR radiologic* OR roentgenological* OR roentgenology OR radiology OR Radiography OR Diagnostic X-Ray OR Diagnostic X Ray OR Diagnostic X-Rays OR X-Rays, Diagnostic OR X-Ray, Diagnostic OR X Ray, Diagnostic OR Diagnostic X-Ray Radiology OR Diagnostic X Ray Radiology OR X-Ray Radiology, Diagnostic OR X Ray Radiology, Diagnostic OR Radiology, Diagnostic X-Ray OR Radiology, Diagnostic X Ray OR dual-energy scanned projection radiography OR electroradiography OR pneumoradiography OR radiogram OR radiographic method OR radiography, dual-energy scanned projection OR radioimaging OR radiophotography OR roentgen photography OR roentgenography OR roentgenoscopy OR rontgenography OR X ray imaging OR x ray photography OR computer assisted positron emission tomography OR positron emission computed tomography OR positron emission tomography computed tomography OR positron emission tomography, computer assisted OR positron-emission tomography and computed tomography OR positron emission tomography-computed tomography OR PET-CT Scan OR PET-CT Scans OR Scan, PET-CT OR Scans, PET-CT OR PET CT Scan OR CT Scan, PET OR CT Scans, PET OR PET CT Scans OR Scan, PET CT OR Scans, PET CT OR PET-CT OR CT PET OR CT PET Scan OR CT PET Scans OR PET Scan, CT OR PET Scans, CT OR Scan, CT PET OR Scans, CT PET OR p.e.t. OR PET scanning OR positron emission tomographic scan OR positron emission tomographic scanning OR positron tomography OR positron-emission tomography OR tomography, positron OR positron emission tomography OR PET Imaging OR Imaging, PET OR PET Imagings OR Positron-Emission Tomography Imaging OR Imaging, Positron-Emission Tomography OR Positron Emission Tomography Imaging OR Positron-Emission Tomography Imagings OR Tomography Imaging, Positron-Emission OR PET Scan OR PET Scans OR Scan, PET OR Tomography, Positron-Emission OR Tomography, Positron Emission OR diagnostic ultrasonic examination OR diagnostic ultrasonic imaging OR diagnostic ultrasonic method OR doptone OR duplex echography OR echogram OR echographic evaluation OR echoscopy OR echosound OR high resolution echography OR scanning, ultrasonic OR sonogram OR sonographic examination OR sonographic screening OR sonography OR ultrasonic detection OR ultrasonic echo OR ultrasonic examination OR ultrasonic scanning OR ultrasonic scintillation OR ultrasonogram OR ultrasonographic examination OR ultrasonographic screening OR ultrasonography OR ultrasound diagnosis OR ultrasound scanning OR Echotomography OR Ultrasonic Imaging OR Imaging, Ultrasonic OR Sonography, Medical OR Medical Sonography OR Ultrasound Imaging OR Imagings, Ultrasound OR Imaging, Ultrasound OR Ultrasonographic Imaging OR Imagings, Ultrasonographic OR Imaging, Ultrasonographic OR Ultrasonographic Imagings OR Echography OR Diagnostic Ultrasound OR Diagnostic Ultrasounds OR Ultrasound, Diagnostic OR Ultrasounds, Diagnostic OR Echotomography, Computer OR Computer Echotomography OR Tomography, Ultrasonic OR Ultrasonic Tomography OR Diagnosis, Ultrasonic OR Diagnoses, Ultrasonic OR Ultrasonic Diagnoses OR Ultrasonic Diagnosis OR Echomammography OR breast echography OR breast ultrasound OR echography, breast OR sonography, breast OR Ultrasonography, Mammary OR Ultrasonic Mammography OR Mammographies, Ultrasonic OR Ultrasonic Mammographies OR Mammography, Ultrasonic OR Mammography, Ultrasound OR Mammographies, Ultrasound OR Ultrasound Mammographies OR Ultrasound Mammography OR Mammary Ultrasonography OR Mammary Ultrasonographies OR Ultrasonographies, Mammary OR Ultrasonography, Breast OR Breast Ultrasonography OR Breast Ultrasonographies OR Ultrasonographies, Breast)**
5. **TS=(computer-assisted image processing OR processing, image OR image processing OR Image Processing, Computer Assisted OR Computer-Assisted Image Processing OR Computer Assisted Image Processing OR Image Analysis, Computer-Assisted OR Computer-Assisted Image Analyses OR Image Analyses, Computer-Assisted OR Image Analysis, Computer Assisted OR Analysis, Computer-Assisted Image OR Computer-Assisted Image Analysis OR Computer Assisted Image Analysis OR Image Reconstruction OR Image Reconstructions OR Reconstruction, Image OR Reconstructions, Image OR Digital Image Processing OR Image Processing, Digital OR Processing, Digital Image OR Processings, Digital Image OR Medical Image Processing OR Image Processing, Medical OR Image Processings, Medical OR Medical Image Processings OR Processing, Medical Image OR Processings, Medical Image OR Biomedical Image Processing OR Image Processing, Biomedical OR Processing, Biomedical Image OR Image Interpretation, Computer-Assisted OR Computer-Assisted Image Interpretation OR Computer-Assisted Image Interpretations OR Image Interpretations, Computer-Assisted OR Interpretation, Computer-Assisted Image OR Interpretations, Computer-Assisted Image OR Image Interpretation, Computer Assisted OR computer assisted diagnosis OR automatic diagnosis OR computer diagnosis OR computer-assisted diagnosis OR computer-assisted radiographic image interpretation OR diagnosis, computer OR diagnosis, computer-assisted OR radiographic image interpretation, computer-assisted OR computer assisted diagnosis OR Radiomi*)**
6. **TS=(Comparative Genomics OR Genomics, Comparative OR Structural Genomics OR Genomics, Structural OR Functional Genomics OR Genomics, Functional OR genome components OR genome, helminth OR genome, protozoan OR genome, protozoon OR protozoan genome OR protozoon genome OR Genome* OR expression*, gene OR genetic expression OR genome expression OR gene expression* OR dosage compensation OR dosage compensation, genetic OR gene dosage compensation OR gene expression regulation, archaeal OR gene expression regulation, bacterial OR gene expression regulation, developmental OR gene expression regulation, enzymologic OR gene expression regulation, fungal OR gene expression regulation, leukaemic OR gene expression regulation, leukemic OR gene expression regulation, neoplastic OR gene expression regulation, plant OR gene expression regulation, viral OR genetic dosage compensation OR Regulation, Gene Action OR Gene Action Regulation OR Expression Regulation, Gene OR Regulation, Gene Expression OR Regulation of Gene Expression OR gene expression regulation OR epigenomic* OR Epigenetic* OR Gene Ontologies OR Ontologies, Gene OR Ontology, Gene OR Gene Ontology Project OR Gene Ontology Projects OR Ontology Project, Gene OR Ontology Projects, Gene OR Project, Gene Ontology OR Projects, Gene Ontology OR gene ontology OR gene expression analysis OR gene expression profile OR gene product profiling OR gene expression profiling* OR Profiling, Gene Expression OR Profilings, Gene Expression OR Gene Expression Monitoring OR Gene Expression Monitorings OR Monitoring, Gene Expression OR Monitorings, Gene Expression OR Gene Expression Pattern Analysis OR Transcript Expression Analysis OR Analyses, Transcript Expression OR Analysis, Transcript Expression OR Transcript Expression Analyses OR Transcriptome Analysis OR Analyses, Transcriptome OR Analysis, Transcriptome OR Transcriptome Analyses OR Transcriptome Profiling OR Profilings, Transcriptome OR Profiling, Transcriptome OR Transcriptome Profilings OR Transcriptomics OR Gene Expression Analysis OR Analyses, Gene Expression OR Analysis, Gene Expression OR Expression Analyses, Gene OR Expression Analysis, Gene OR Gene Expression Analyses OR mRNA Differential Display OR Differential Display, mRNA OR Differential Displays, mRNA OR mRNA Differential Displays OR code, genetic OR deoxyribonucleic acid code OR dna code OR genetic coding OR genetic code* OR codes, genetic OR transcriptome* OR Gene Expression Profiles OR Expression Profile, Gene OR Expression Profiles, Gene OR Gene Expression Profile OR Profile, Gene Expression OR Profiles, Gene Expression OR Gene Expression Signatures OR Expression Signature, Gene OR Expression Signatures, Gene OR Gene Expression Signature OR Signature, Gene Expression OR Signatures, Gene Expression OR Transcriptome Profiles OR Profiles, Transcriptome OR Profile, Transcriptome OR Transcriptome Profile OR b rna OR brna OR messenger ribonucleic acid OR ribonucleic acid, messenger OR rna, messenger OR RNA, messenger, stored OR rna, messenger OR Messenger RNA OR mRNA OR mRNA, Non-Polyadenylated OR mRNA, Non Polyadenylated OR Non-Polyadenylated mRNA OR Non Polyadenylated mRNA OR mRNA, Polyadenylated OR Polyadenylated mRNA OR Messenger RNA, Polyadenylated OR Polyadenylated Messenger RNA OR RNA, Polyadenylated Messenger OR RNA, Messenger, Polyadenylated OR Poly(A)+ mRNA OR RNA, Polyadenylated OR Polyadenylated RNA OR RNA seq OR RNA-Seq OR RNAseq OR sequence analysis, RNA OR Analyses, RNA Sequence OR RNA Sequence Analyses OR Sequence Analyses, RNA OR Analysis, RNA Sequence OR RNA Sequence Analysis OR Sequence Determinations, RNA OR Determination, RNA Sequence OR Determinations, RNA Sequence OR RNA Sequence Determination OR RNA Sequence Determinations OR RNA Sequencing OR Sequencing, RNA OR Sequence Determination, RNA OR amino acid sequence analysis OR amino acid sequencing OR aminoacid sequence analysis OR genetic sequencing OR genomic sequencing OR macromolecular sequencing OR molecular sequencing OR peptide sequence analysis OR peptide sequencing OR protein sequence analysis OR protein sequencing OR sequence analysis, protein OR Analysis, Sequence OR Analyses, Sequence OR Sequence Analyses OR Sequence Determinations OR Determination, Sequence OR Determinations, Sequence OR Sequence Determination OR sequence analysis OR DNA sequence determination OR DNA sequencing OR Analyses, DNA Sequence OR DNA Sequence Analyses OR Sequence Analys*, DNA OR Analysis, DNA Sequence OR DNA Sequence Analysis OR Sequence Determinations, DNA OR Determination, DNA Sequence OR Determinations, DNA Sequence OR DNA Sequence Determination OR DNA Sequence Determinations OR DNA Sequencing OR Sequencing, DNA OR Sequence Determination, DNA OR entire genome sequencing OR full genome sequencing OR WGS OR WGS analysis OR Genome Sequencing, Whole OR Sequencing, Whole Genome OR Complete Genome Sequencing OR Genome Sequencing, Complete OR Sequencing, Complete Genome OR whole genome sequencing OR prote-omics OR protein omics OR proteinomics OR Proteomics OR Peptidomics OR deoxyribonucleic acid methylation OR DNA hypermethylation OR DNA hypomethylation OR methylated deoxyribonucleic acid OR methylated dna OR DNA methylation* OR Methylation, DNA OR Methylations, DNA OR Analysis, DNA Mutational OR Analyses, DNA Mutational OR DNA Mutational Analyses OR Mutational Analyses, DNA OR Mutational Analysis, DNA OR community genomic OR community genomics OR eco-genomics OR ecogenomic OR ecogenomics OR ecological genomic OR ecological genomics OR environmental genomic OR environmental genomics OR meta-genomics OR Metagenomic OR Metagenomics OR Genomics, Environmental OR Genomics, Community OR Population Genomics OR Genomics, Population)**
7. **TS=(imaging radiogenomics OR neuroimaging genomics OR imaging genomics OR Genomics, Imaging OR Radiogenomic* OR radiation genomics OR radio-genomics OR Oncology, Radiation OR Therapeutic Radiology OR Radiology, Therapeutic OR radiation oncology OR Biology, Radiation OR Radiation Biology OR radio biology OR radiobiology)**
8. **#2 AND #3**
9. **#7 OR #4**
10. **#8 AND #5**
11. **#9 OR #6**
12. **#10 AND #1**
13. **Embase (n = 3540)**
14. 'breast tumor'/exp
15. 'Breast Neoplasm':ab,ti OR 'Breast Neoplasms':ab,ti OR 'Neoplasm, Breast':ab,ti OR 'Neoplasms, Breast':ab,ti OR 'Breast Tumors':ab,ti OR 'Breast Tumor':ab,ti OR 'Tumor, Breast':ab,ti OR 'Tumors, Breast':ab,ti OR 'Breast Cancer':ab,ti OR 'Cancer, Breast':ab,ti OR 'Cancer of Breast':ab,ti OR 'Cancer of the Breast':ab,ti OR 'Malignant Neoplasm of Breast':ab,ti OR 'Breast Malignant Neoplasm':ab,ti OR 'Breast Malignant Neoplasms':ab,ti OR 'Malignant Tumor of Breast':ab,ti OR 'Breast Malignant Tumor':ab,ti OR 'Breast Malignant Tumors':ab,ti OR 'Mammary Cancer':ab,ti OR 'Cancer, Mammary':ab,ti OR 'Cancers, Mammary':ab,ti OR 'Mammary Cancers':ab,ti OR 'Mammary Neoplasms, Human':ab,ti OR 'Human Mammary Neoplasm':ab,ti OR 'Human Mammary Neoplasms':ab,ti OR 'Neoplasm, Human Mammary':ab,ti OR 'Neoplasms, Human Mammary':ab,ti OR 'Mammary Neoplasm, Human':ab,ti OR 'Breast Carcinoma':ab,ti OR 'Breast Carcinomas':ab,ti OR 'Carcinoma, Breast':ab,ti OR 'Carcinomas, Breast':ab,ti OR 'Mammary Carcinoma, Human':ab,ti OR 'Carcinoma, Human Mammary':ab,ti OR 'Carcinomas, Human Mammary':ab,ti OR 'Human Mammary Carcinomas':ab,ti OR 'Mammary Carcinomas, Human':ab,ti OR 'Human Mammary Carcinoma':ab,ti OR 'bilateral breast neoplasm':ab,ti OR 'bilateral breast tumor':ab,ti OR 'bilateral breast tumour':ab,ti OR 'breast gland tumor':ab,ti OR 'breast gland tumour':ab,ti OR 'breast mass':ab,ti OR 'breast neoplasia':ab,ti OR 'breast neoplasm':ab,ti OR 'breast neoplasms':ab,ti OR 'breast neoplasms, male':ab,ti OR 'breast tumorigenesis':ab,ti OR 'breast tumour':ab,ti OR 'female breast neoplasm':ab,ti OR 'female breast tumor':ab,ti OR 'female breast tumour':ab,ti OR 'male breast neoplasm':ab,ti OR 'male breast tumor':ab,ti OR 'male breast tumour':ab,ti OR 'mamma tumor':ab,ti OR 'mamma tumour':ab,ti OR 'mammary gland neoplasia':ab,ti OR 'mammary gland neoplasm':ab,ti OR 'mammary gland tumor':ab,ti OR 'mammary gland tumorigenesis':ab,ti OR 'mammary gland tumour':ab,ti OR 'mammary neoplasia':ab,ti OR 'mammary neoplasm':ab,ti OR 'mammary neoplasms':ab,ti OR 'mammary tumor':ab,ti OR 'mammary tumor cell':ab,ti OR 'mammary tumorigenesis':ab,ti OR 'mammary tumour':ab,ti OR 'mammary tumour cell':ab,ti OR 'mass in the breast':ab,ti OR 'masses in the breast':ab,ti OR 'neoplasia of the breast':ab,ti OR 'neoplasm of the breast':ab,ti OR 'neoplasm of the mammary gland':ab,ti OR 'neoplastic breast':ab,ti OR 'neoplastic mammary':ab,ti OR 'neoplastic mammary gland':ab,ti OR 'tumor of the breast':ab,ti OR 'tumor of the female breast':ab,ti OR 'tumor of the male breast':ab,ti OR 'tumor of the mammary gland':ab,ti OR 'tumorigenesis of the breast':ab,ti OR 'tumorigenesis of the mammary gland':ab,ti OR 'tumour of the male breast':ab,ti OR 'unilateral breast neoplasm':ab,ti OR 'unilateral breast neoplasms':ab,ti OR 'unilateral breast tumor':ab,ti OR 'breast gland cancer':ab,ti OR 'breast gland neoplasm':ab,ti OR 'breast malignancies':ab,ti OR 'breast malignancy':ab,ti OR 'breast tumor malignant':ab,ti OR 'Ca breast':ab,ti OR 'cancer in the mammary gland':ab,ti OR 'cancer of the mammary gland':ab,ti OR 'cancer, breast':ab,ti OR 'malignancies of the breast':ab,ti OR 'malignancy of the breast':ab,ti OR 'malignant breast neoplasm':ab,ti OR 'malignant breast tumor':ab,ti OR 'malignant neoplasm of the breast':ab,ti OR 'malignant tumor of the breast':ab,ti OR 'mamma cancer':ab,ti OR 'mammary gland cancer':ab,ti OR 'mammary gland malignancy':ab,ti OR 'mammary malignancies':ab,ti OR 'mammary malignancy':ab,ti OR 'breast carcinomata':ab,ti OR 'breast carcinomatosis':ab,ti OR 'carcinoma in the mammary gland':ab,ti OR 'carcinoma of the breast':ab,ti OR 'carcinoma of the mamma':ab,ti OR 'carcinoma of the mammary gland':ab,ti OR 'carcinoma, mammary':ab,ti OR 'carcinomata of the breast':ab,ti OR 'carcinomatous breast':ab,ti OR 'carcinomatous mammary':ab,ti OR 'carcinomatous mammary gland':ab,ti OR 'human mammary carcinoma':ab,ti OR 'mamma carcinoma':ab,ti OR 'mammary carcinoma':ab,ti OR 'mammary carcinomata':ab,ti OR 'mammary carcinomatosis':ab,ti OR 'mammary gland carcinoma':ab,ti OR 'adenocarcinoma of the breast':ab,ti OR 'adenocarcinoma of the mamma':ab,ti OR 'adenocarcinoma of the mammary gland':ab,ti OR 'mammary adenocarcinoma':ab,ti OR 'breast adenocarcinoma':ab,ti OR 'Carcinoma, Ductal, Breast':ab,ti OR 'Carcinoma, Infiltrating Duct':ab,ti OR 'Carcinomas, Infiltrating Duct':ab,ti OR 'Carcinoma, Invasive Ductal, Breast':ab,ti OR 'Invasive Ductal Carcinoma, Breast':ab,ti OR 'Carcinoma, Mammary Ductal':ab,ti OR 'Carcinomas, Mammary Ductal':ab,ti OR 'Mammary Ductal Carcinomas':ab,ti OR 'Mammary Ductal Carcinoma':ab,ti OR 'breast duct carcinoma':ab,ti OR 'breast ductal adenocarcinoma':ab,ti OR 'carcinoma, ductal, breast':ab,ti OR 'ductal adenocarcinoma of the breast':ab,ti OR 'ductal breast adenocarcinoma':ab,ti OR 'ductal breast carcinoma':ab,ti OR 'ductal carcinoma of the breast':ab,ti OR 'ductal mammary carcinoma':ab,ti OR 'mammary duct carcinoma':ab,ti OR 'breast ductal carcinoma':ab,ti OR 'breast invasive ductal carcinoma':ab,ti OR 'ductal invasive breast carcinoma':ab,ti OR 'infiltrating duct carcinoma of breast':ab,ti OR 'infiltrating ductal breast cancer':ab,ti OR 'infiltrating ductal breast carcinoma':ab,ti OR 'infiltrating ductal carcinoma of the breast':ab,ti OR 'invasive ductal breast cancer':ab,ti OR 'invasive ductal carcinoma of the breast':ab,ti OR 'invasive ductal breast carcinoma':ab,ti OR 'Carcinoma, Lobular':ab,ti OR 'Carcinomas, Lobular':ab,ti OR 'Lobular Carcinoma':ab,ti OR 'Lobular Carcinomas':ab,ti OR 'carcinoma, lobular':ab,ti OR 'lobular adenocarcinoma':ab,ti OR 'lobular adenocarcinoma of the breast':ab,ti OR 'lobular breast adenocarcinoma':ab,ti OR 'lobular breast carcinoma':ab,ti OR 'lobular cancer':ab,ti OR 'lobular cancer of the breast':ab,ti OR 'lobular carcinoma of the breast':ab,ti OR 'lobular carcinoma':ab,ti OR 'infiltrating lobular breast cancer':ab,ti OR 'infiltrating lobular breast carcinoma':ab,ti OR 'infiltrating lobular carcinoma':ab,ti OR 'infiltrative lobular carcinoma':ab,ti OR 'invasive lobular breast cancer':ab,ti OR 'invasive lobular cancer of the breast':ab,ti OR 'invasive lobular carcinoma':ab,ti OR 'invasive lobular carcinoma of the breast':ab,ti OR 'invasive lobular breast carcinoma':ab,ti
16. 'artificial intelligence'/exp
17. 'computer assisted diagnosis':ab,ti OR 'automatic diagnosis':ab,ti OR 'computer diagnosis':ab,ti OR 'computer-assisted diagnosis':ab,ti OR 'computer-assisted image interpretation':ab,ti OR 'computer-assisted radiographic image interpretation':ab,ti OR 'diagnosis, computer':ab,ti OR 'diagnosis, computer-assisted':ab,ti OR 'image interpretation, computer-assisted':ab,ti OR 'radiographic image interpretation, computer-assisted':ab,ti OR 'Intelligence, Artificial':ab,ti OR 'Computer Reasoning':ab,ti OR 'Reasoning, Computer':ab,ti OR 'AI (Artificial Intelligence)':ab,ti OR 'Machine Intelligence':ab,ti OR 'Intelligence, Machine':ab,ti OR 'Computational Intelligence':ab,ti OR 'Intelligence, Computational':ab,ti OR 'Computer Vision Systems':ab,ti OR 'Computer Vision System':ab,ti OR 'System, Computer Vision':ab,ti OR 'Systems, Computer Vision':ab,ti OR 'Vision System, Computer':ab,ti OR 'Vision Systems, Computer':ab,ti OR 'artificial intelligence':ab,ti OR 'machine learning':ab,ti OR 'learning machine':ab,ti OR 'learning machines':ab,ti OR 'Learning, Machine':ab,ti OR 'Transfer Learning':ab,ti OR 'Learning, Transfer':ab,ti OR 'artificial neural network':ab,ti OR 'algorithmic neural network':ab,ti OR 'ANN':ab,ti OR 'ANN analysis':ab,ti OR 'ANN approach':ab,ti OR 'ANN method':ab,ti OR 'ANN methodology':ab,ti OR 'ANN methods':ab,ti OR 'ANN model':ab,ti OR 'ANN modeling':ab,ti OR 'ANN modelling':ab,ti OR 'ANN models':ab,ti OR 'ANN output':ab,ti OR 'ANN technique':ab,ti OR 'ANN techniques':ab,ti OR 'ANN training':ab,ti OR 'ANNs':ab,ti OR 'artificial neural networks':ab,ti OR 'artificial NN':ab,ti OR 'artificial NNs':ab,ti OR 'computational neural network':ab,ti OR 'computer neural network':ab,ti OR 'computer neural networks':ab,ti OR 'computerized neural network':ab,ti OR 'connectionist model':ab,ti OR 'connectionist network':ab,ti OR 'connectionist neural network':ab,ti OR 'connectionist system':ab,ti OR 'mathematical neural network':ab,ti OR 'neural network':ab,ti OR 'neural network algorithm':ab,ti OR 'neural network model':ab,ti OR 'neural networks ':ab,ti OR 'neural networks, computer':ab,ti OR 'deep learning':ab,ti OR 'deep machine learning':ab,ti OR 'deep ML':ab,ti OR 'hierarchical learning':ab,ti OR 'Learning, Deep':ab,ti OR 'Learning, Hierarchical':ab,ti
18. 'Radiology'/exp
19. 'diagnostic imaging'/exp
20. 'imaging, diagnostic':ab,ti OR 'imaging, medical':ab,ti OR 'medical imaging':ab,ti OR 'ophthalmo diaphanoscopy':ab,ti OR 'diagnostic imaging':ab,ti OR 'imaging, magnetization transfer':ab,ti OR 'magnetic resonance imaging':ab,ti OR 'magnetic resonance tomography':ab,ti OR 'magnetization transfer imaging':ab,ti OR 'mr imaging':ab,ti OR 'MRI':ab,ti OR 'nuclear magnetic resonance imaging':ab,ti OR 'Imaging, Magnetic Resonance':ab,ti OR 'NMR Imaging':ab,ti OR 'Imaging, NMR':ab,ti OR 'Zeugmatography':ab,ti OR 'Tomography, MR':ab,ti OR 'Steady-State Free Precession MRI':ab,ti OR 'Steady State Free Precession MRI':ab,ti OR 'NMR Tomography':ab,ti OR 'Tomography, NMR':ab,ti OR 'MR Tomography':ab,ti OR 'Tomography, Proton Spin':ab,ti OR 'Proton Spin Tomography':ab,ti OR 'Magnetization Transfer Contrast Imaging':ab,ti OR 'fMRI':ab,ti OR 'Magnetic Resonance Imaging, Functional':ab,ti OR 'MRI, Functional':ab,ti OR 'Functional MRI':ab,ti OR 'Functional MRIs':ab,ti OR 'MRIs, Functional':ab,ti OR 'Functional Magnetic Resonance Imaging':ab,ti OR 'MRI Scans':ab,ti OR 'MRI Scan':ab,ti OR 'Scan, MRI':ab,ti OR 'Scans, MRI':ab,ti OR 'Imaging, Chemical Shift':ab,ti OR 'Chemical Shift Imagings':ab,ti OR 'Imagings, Chemical Shift':ab,ti OR 'Shift Imaging, Chemical':ab,ti OR 'Shift Imagings, Chemical':ab,ti OR 'Chemical Shift Imaging':ab,ti OR 'Spin Echo Imaging':ab,ti OR 'Echo Imaging, Spin':ab,ti OR 'Echo Imagings, Spin':ab,ti OR 'Imaging, Spin Echo':ab,ti OR 'Imagings, Spin Echo':ab,ti OR 'Spin Echo Imagings':ab,ti OR 'Magnetic Resonance Image':ab,ti OR 'Image, Magnetic Resonance':ab,ti OR 'Magnetic Resonance Images':ab,ti OR 'Resonance Image, Magnetic':ab,ti OR 'breast MRI':ab,ti OR 'magnetic resonance breast imaging':ab,ti OR 'magnetic resonance mammography':ab,ti OR 'breast magnetic resonance imaging':ab,ti OR 'Mammography':ab,ti OR 'mamilloscopy':ab,ti OR 'mammilloscopy':ab,ti OR 'mammo-graphy':ab,ti OR 'mammogram':ab,ti OR 'mastography Mammographies':ab,ti OR 'Digital Breast Tomosynthesis':ab,ti OR 'Breast Tomosyntheses, Digital':ab,ti OR 'Breast Tomosynthesis, Digital':ab,ti OR 'Digital Breast Tomosyntheses':ab,ti OR 'X-ray Breast Tomosynthesis':ab,ti OR 'Breast Tomosyntheses, X-ray':ab,ti OR 'Breast Tomosynthesis, X-ray':ab,ti OR 'X-ray Breast Tomosyntheses':ab,ti OR 'X ray Breast Tomosynthesis':ab,ti OR '3D-Mammography':ab,ti OR '3D-Mammographies':ab,ti OR '3D Mammography':ab,ti OR 'Digital Mammography':ab,ti OR 'Digital Mammographies':ab,ti OR 'Mammographies, Digital':ab,ti OR 'Mammography, Digital':ab,ti OR 'diagnostic radiology':ab,ti OR 'radio diagnosis':ab,ti OR 'radiographic examination':ab,ti OR 'radiologic diagnosis':ab,ti OR 'radiologic examination':ab,ti OR 'radiological diagnosis':ab,ti OR 'radiological examination':ab,ti OR 'radiology, diagnostic':ab,ti OR 'roentgen diagnosis':ab,ti OR 'roentgen diagnostics':ab,ti OR 'roentgen examination':ab,ti OR 'roentgen screening':ab,ti OR 'roentgenologic diagnosis':ab,ti OR 'roentgenologic diagnostics':ab,ti OR 'roentgenologic examination':ab,ti OR 'roentgenologic screening':ab,ti OR 'roentgenological diagnosis':ab,ti OR 'roentgenological diagnostics':ab,ti OR 'roentgenological examination':ab,ti OR 'roentgenological screening':ab,ti OR 'rontgen diagnosis':ab,ti OR 'rontgen examination':ab,ti OR 'rontgenologic diagnosis':ab,ti OR 'rontgenologic examination':ab,ti OR 'rontgenological diagnosis':ab,ti OR 'rontgenological examination':ab,ti OR 'X ray diagnosis':ab,ti OR 'X ray examination':ab,ti OR 'radiodiagnosis':ab,ti OR 'radiologic*':ab,ti OR 'roentgenological*':ab,ti OR 'roentgenology':ab,ti OR 'radiology':ab,ti OR 'Radiography':ab,ti OR 'Diagnostic X-Ray':ab,ti OR 'Diagnostic X Ray':ab,ti OR 'Diagnostic X-Rays':ab,ti OR 'X-Rays, Diagnostic':ab,ti OR 'X-Ray, Diagnostic':ab,ti OR 'X Ray, Diagnostic':ab,ti OR 'Diagnostic X-Ray Radiology':ab,ti OR 'Diagnostic X Ray Radiology':ab,ti OR 'X-Ray Radiology, Diagnostic':ab,ti OR 'X Ray Radiology, Diagnostic':ab,ti OR 'Radiology, Diagnostic X-Ray':ab,ti OR 'Radiology, Diagnostic X Ray':ab,ti OR 'dual-energy scanned projection radiography':ab,ti OR 'electroradiography':ab,ti OR 'pneumoradiography':ab,ti OR 'radiogram':ab,ti OR 'radiographic method':ab,ti OR 'radiography, dual-energy scanned projection':ab,ti OR 'radioimaging':ab,ti OR 'radiophotography':ab,ti OR 'roentgen photography':ab,ti OR 'roentgenography':ab,ti OR 'roentgenoscopy':ab,ti OR 'rontgenography':ab,ti OR 'X ray imaging':ab,ti OR 'x ray photography ':ab,ti OR 'computer assisted positron emission tomography':ab,ti OR 'positron emission computed tomography':ab,ti OR 'positron emission tomography computed tomography':ab,ti OR 'positron emission tomography, computer assisted':ab,ti OR 'positron-emission tomography and computed tomography':ab,ti OR 'positron emission tomography-computed tomography':ab,ti OR 'PET-CT Scan':ab,ti OR 'PET-CT Scans':ab,ti OR 'Scan, PET-CT':ab,ti OR 'Scans, PET-CT':ab,ti OR 'PET CT Scan':ab,ti OR 'CT Scan, PET':ab,ti OR 'CT Scans, PET':ab,ti OR 'PET CT Scans':ab,ti OR 'Scan, PET CT':ab,ti OR 'Scans, PET CT':ab,ti OR 'PET-CT':ab,ti OR 'CT PET':ab,ti OR 'CT PET Scan':ab,ti OR 'CT PET Scans':ab,ti OR 'PET Scan, CT':ab,ti OR 'PET Scans, CT':ab,ti OR 'Scan, CT PET':ab,ti OR 'Scans, CT PET':ab,ti OR 'p.e.t.':ab,ti OR 'PET scanning':ab,ti OR 'positron emission tomographic scan':ab,ti OR 'positron emission tomographic scanning':ab,ti OR 'positron tomography':ab,ti OR 'positron-emission tomography':ab,ti OR 'tomography, positron':ab,ti OR 'positron emission tomography':ab,ti OR 'PET Imaging':ab,ti OR 'Imaging, PET':ab,ti OR 'PET Imagings':ab,ti OR 'Positron-Emission Tomography Imaging':ab,ti OR 'Imaging, Positron-Emission Tomography':ab,ti OR 'Positron Emission Tomography Imaging':ab,ti OR 'Positron-Emission Tomography Imagings':ab,ti OR 'Tomography Imaging, Positron-Emission':ab,ti OR 'PET Scan':ab,ti OR 'PET Scans':ab,ti OR 'Scan, PET':ab,ti OR 'Tomography, Positron-Emission':ab,ti OR 'Tomography, Positron Emission':ab,ti OR 'diagnostic ultrasonic examination':ab,ti OR 'diagnostic ultrasonic imaging':ab,ti OR 'diagnostic ultrasonic method':ab,ti OR 'doptone':ab,ti OR 'duplex echography':ab,ti OR 'echogram':ab,ti OR 'echographic evaluation':ab,ti OR 'echoscopy':ab,ti OR 'echosound':ab,ti OR 'high resolution echography':ab,ti OR 'scanning, ultrasonic':ab,ti OR 'sonogram':ab,ti OR 'sonographic examination':ab,ti OR 'sonographic screening':ab,ti OR 'sonography':ab,ti OR 'ultrasonic detection':ab,ti OR 'ultrasonic echo':ab,ti OR 'ultrasonic examination':ab,ti OR 'ultrasonic scanning':ab,ti OR 'ultrasonic scintillation':ab,ti OR 'ultrasonogram':ab,ti OR 'ultrasonographic examination':ab,ti OR 'ultrasonographic screening':ab,ti OR 'ultrasonography':ab,ti OR 'ultrasound diagnosis':ab,ti OR 'ultrasound scanning ':ab,ti OR 'Echotomography':ab,ti OR 'Ultrasonic Imaging':ab,ti OR 'Imaging, Ultrasonic':ab,ti OR 'Sonography, Medical':ab,ti OR 'Medical Sonography':ab,ti OR 'Ultrasound Imaging':ab,ti OR 'Imagings, Ultrasound':ab,ti OR 'Imaging, Ultrasound':ab,ti OR 'Ultrasonographic Imaging':ab,ti OR 'Imagings, Ultrasonographic':ab,ti OR 'Imaging, Ultrasonographic':ab,ti OR 'Ultrasonographic Imagings':ab,ti OR 'Echography':ab,ti OR 'Diagnostic Ultrasound':ab,ti OR 'Diagnostic Ultrasounds':ab,ti OR 'Ultrasound, Diagnostic':ab,ti OR 'Ultrasounds, Diagnostic':ab,ti OR 'Echotomography, Computer':ab,ti OR 'Computer Echotomography':ab,ti OR 'Tomography, Ultrasonic':ab,ti OR 'Ultrasonic Tomography':ab,ti OR 'Diagnosis, Ultrasonic':ab,ti OR 'Diagnoses, Ultrasonic':ab,ti OR 'Ultrasonic Diagnoses':ab,ti OR 'Ultrasonic Diagnosis':ab,ti OR 'Echomammography':ab,ti OR 'breast echography':ab,ti OR 'breast ultrasound':ab,ti OR 'echography, breast':ab,ti OR 'sonography, breast':ab,ti OR 'Ultrasonography, Mammary':ab,ti OR 'Ultrasonic Mammography':ab,ti OR 'Mammographies, Ultrasonic':ab,ti OR 'Ultrasonic Mammographies':ab,ti OR 'Mammography, Ultrasonic':ab,ti OR 'Mammography, Ultrasound':ab,ti OR 'Mammographies, Ultrasound':ab,ti OR 'Ultrasound Mammographies':ab,ti OR 'Ultrasound Mammography':ab,ti OR 'Mammary Ultrasonography':ab,ti OR 'Mammary Ultrasonographies':ab,ti OR 'Ultrasonographies, Mammary':ab,ti OR 'Ultrasonography, Breast':ab,ti OR 'Breast Ultrasonography':ab,ti OR 'Breast Ultrasonographies':ab,ti OR 'Ultrasonographies, Breast':ab,ti
21. 'image processing'/exp
22. 'computer-assisted image processing':ab,ti OR 'processing, image':ab,ti OR 'image processing':ab,ti OR 'Image Processing, Computer Assisted':ab,ti OR 'Computer-Assisted Image Processing':ab,ti OR 'Computer Assisted Image Processing':ab,ti OR 'Image Analysis, Computer-Assisted':ab,ti OR 'Computer-Assisted Image Analyses':ab,ti OR 'Image Analyses, Computer-Assisted':ab,ti OR 'Image Analysis, Computer Assisted':ab,ti OR 'Analysis, Computer-Assisted Image':ab,ti OR 'Computer-Assisted Image Analysis':ab,ti OR 'Computer Assisted Image Analysis':ab,ti OR 'Image Reconstruction':ab,ti OR 'Image Reconstructions':ab,ti OR 'Reconstruction, Image':ab,ti OR 'Reconstructions, Image':ab,ti OR 'Digital Image Processing':ab,ti OR 'Image Processing, Digital':ab,ti OR 'Processing, Digital Image':ab,ti OR 'Processings, Digital Image':ab,ti OR 'Medical Image Processing':ab,ti OR 'Image Processing, Medical':ab,ti OR 'Image Processings, Medical':ab,ti OR 'Medical Image Processings':ab,ti OR 'Processing, Medical Image':ab,ti OR 'Processings, Medical Image':ab,ti OR 'Biomedical Image Processing':ab,ti OR 'Image Processing, Biomedical':ab,ti OR 'Processing, Biomedical Image':ab,ti OR 'Image Interpretation, Computer-Assisted':ab,ti OR 'Computer-Assisted Image Interpretation':ab,ti OR 'Computer-Assisted Image Interpretations':ab,ti OR 'Image Interpretations, Computer-Assisted':ab,ti OR 'Interpretation, Computer-Assisted Image':ab,ti OR 'Interpretations, Computer-Assisted Image':ab,ti OR 'Image Interpretation, Computer Assisted':ab,ti OR 'computer assisted diagnosis':ab,ti OR 'automatic diagnosis':ab,ti OR 'computer diagnosis':ab,ti OR 'computer-assisted diagnosis':ab,ti OR 'computer-assisted radiographic image interpretation':ab,ti OR 'diagnosis, computer':ab,ti OR 'diagnosis, computer-assisted':ab,ti OR 'radiographic image interpretation, computer-assisted':ab,ti OR 'computer assisted diagnosis':ab,ti OR 'Radiomi*':ab,ti
23. 'Genomics'/exp
24. 'Genome'/exp
25. 'gene expression'/exp
26. 'gene expression regulation'/exp
27. 'Epigenetics'/exp
28. 'gene ontology'/exp
29. 'gene expression profiling'/exp
30. 'genetic code'/exp
31. 'Transcriptome'/exp
32. 'messenger RNA'/exp
33. 'RNA sequencing'/exp
34. 'sequence analysis'/exp
35. 'DNA sequencing'/exp
36. 'whole genome sequencing'/exp
37. 'Proteomics'/exp
38. 'DNA methylation'/exp
39. 'dna mutational analysis'/exp
40. 'Metagenomics'/exp
41. 'Comparative Genomics':ab,ti OR 'Genomics, Comparative':ab,ti OR 'Structural Genomics':ab,ti OR 'Genomics, Structural':ab,ti OR 'Functional Genomics':ab,ti OR 'Genomics, Functional':ab,ti OR 'genome components':ab,ti OR 'genome, helminth':ab,ti OR 'genome, protozoan':ab,ti OR 'genome, protozoon':ab,ti OR 'protozoan genome':ab,ti OR 'protozoon genome':ab,ti OR 'Genome*':ab,ti OR 'expression*, gene':ab,ti OR 'genetic expression':ab,ti OR 'genome expression':ab,ti OR 'gene expression*':ab,ti OR 'dosage compensation':ab,ti OR 'dosage compensation, genetic':ab,ti OR 'gene dosage compensation':ab,ti OR 'gene expression regulation, archaeal':ab,ti OR 'gene expression regulation, bacterial':ab,ti OR 'gene expression regulation, developmental':ab,ti OR 'gene expression regulation, enzymologic':ab,ti OR 'gene expression regulation, fungal':ab,ti OR 'gene expression regulation, leukaemic':ab,ti OR 'gene expression regulation, leukemic':ab,ti OR 'gene expression regulation, neoplastic':ab,ti OR 'gene expression regulation, plant':ab,ti OR 'gene expression regulation, viral':ab,ti OR 'genetic dosage compensation':ab,ti OR 'Regulation, Gene Action':ab,ti OR 'Gene Action Regulation':ab,ti OR 'Expression Regulation, Gene':ab,ti OR 'Regulation, Gene Expression':ab,ti OR 'Regulation of Gene Expression':ab,ti OR 'gene expression regulation':ab,ti OR 'epigenomic*':ab,ti OR 'Epigenetic*':ab,ti OR 'Gene Ontologies':ab,ti OR 'Ontologies, Gene':ab,ti OR 'Ontology, Gene':ab,ti OR 'Gene Ontology Project':ab,ti OR 'Gene Ontology Projects':ab,ti OR 'Ontology Project, Gene':ab,ti OR 'Ontology Projects, Gene':ab,ti OR 'Project, Gene Ontology':ab,ti OR 'Projects, Gene Ontology':ab,ti OR 'gene ontology':ab,ti OR 'gene expression analysis':ab,ti OR 'gene expression profile':ab,ti OR 'gene product profiling':ab,ti OR 'gene expression profiling*':ab,ti OR 'Profiling, Gene Expression':ab,ti OR 'Profilings, Gene Expression':ab,ti OR 'Gene Expression Monitoring':ab,ti OR 'Gene Expression Monitorings':ab,ti OR 'Monitoring, Gene Expression':ab,ti OR 'Monitorings, Gene Expression':ab,ti OR 'Gene Expression Pattern Analysis':ab,ti OR 'Transcript Expression Analysis':ab,ti OR 'Analyses, Transcript Expression':ab,ti OR 'Analysis, Transcript Expression':ab,ti OR 'Transcript Expression Analyses':ab,ti OR 'Transcriptome Analysis':ab,ti OR 'Analyses, Transcriptome':ab,ti OR 'Analysis, Transcriptome':ab,ti OR 'Transcriptome Analyses':ab,ti OR 'Transcriptome Profiling':ab,ti OR 'Profilings, Transcriptome':ab,ti OR 'Profiling, Transcriptome':ab,ti OR 'Transcriptome Profilings':ab,ti OR 'Transcriptomics':ab,ti OR 'Gene Expression Analysis':ab,ti OR 'Analyses, Gene Expression':ab,ti OR 'Analysis, Gene Expression':ab,ti OR 'Expression Analyses, Gene':ab,ti OR 'Expression Analysis, Gene':ab,ti OR 'Gene Expression Analyses':ab,ti OR 'mRNA Differential Display':ab,ti OR 'Differential Display, mRNA':ab,ti OR 'Differential Displays, mRNA':ab,ti OR 'mRNA Differential Displays':ab,ti OR 'code, genetic':ab,ti OR 'deoxyribonucleic acid code':ab,ti OR 'dna code':ab,ti OR 'genetic coding':ab,ti OR 'genetic code*':ab,ti OR 'codes, genetic':ab,ti OR 'transcriptome*':ab,ti OR 'Gene Expression Profiles':ab,ti OR 'Expression Profile, Gene':ab,ti OR 'Expression Profiles, Gene':ab,ti OR 'Gene Expression Profile':ab,ti OR 'Profile, Gene Expression':ab,ti OR 'Profiles, Gene Expression':ab,ti OR 'Gene Expression Signatures':ab,ti OR 'Expression Signature, Gene':ab,ti OR 'Expression Signatures, Gene':ab,ti OR 'Gene Expression Signature':ab,ti OR 'Signature, Gene Expression':ab,ti OR 'Signatures, Gene Expression':ab,ti OR 'Transcriptome Profiles':ab,ti OR 'Profiles, Transcriptome':ab,ti OR 'Profile, Transcriptome':ab,ti OR 'Transcriptome Profile':ab,ti OR 'b rna':ab,ti OR 'brna':ab,ti OR 'messenger ribonucleic acid':ab,ti OR 'ribonucleic acid, messenger':ab,ti OR 'rna, messenger':ab,ti OR 'RNA, messenger, stored':ab,ti OR 'rna, messenger':ab,ti OR 'Messenger RNA':ab,ti OR 'mRNA':ab,ti OR 'mRNA, Non-Polyadenylated':ab,ti OR 'mRNA, Non Polyadenylated':ab,ti OR 'Non-Polyadenylated mRNA':ab,ti OR 'Non Polyadenylated mRNA':ab,ti OR 'mRNA, Polyadenylated':ab,ti OR 'Polyadenylated mRNA':ab,ti OR 'Messenger RNA, Polyadenylated':ab,ti OR 'Polyadenylated Messenger RNA':ab,ti OR 'RNA, Polyadenylated Messenger':ab,ti OR 'RNA, Messenger, Polyadenylated':ab,ti OR 'Poly(A)+ mRNA':ab,ti OR 'RNA, Polyadenylated':ab,ti OR 'Polyadenylated RNA':ab,ti OR 'RNA seq':ab,ti OR 'RNA-Seq':ab,ti OR 'RNAseq':ab,ti OR 'sequence analysis, RNA':ab,ti OR 'Analyses, RNA Sequence':ab,ti OR 'RNA Sequence Analyses':ab,ti OR 'Sequence Analyses, RNA':ab,ti OR 'Analysis, RNA Sequence':ab,ti OR 'RNA Sequence Analysis':ab,ti OR 'Sequence Determinations, RNA':ab,ti OR 'Determination, RNA Sequence':ab,ti OR 'Determinations, RNA Sequence':ab,ti OR 'RNA Sequence Determination':ab,ti OR 'RNA Sequence Determinations':ab,ti OR 'RNA Sequencing':ab,ti OR 'Sequencing, RNA':ab,ti OR 'Sequence Determination, RNA':ab,ti OR 'amino acid sequence analysis':ab,ti OR 'amino acid sequencing':ab,ti OR 'aminoacid sequence analysis':ab,ti OR 'genetic sequencing':ab,ti OR 'genomic sequencing':ab,ti OR 'macromolecular sequencing':ab,ti OR 'molecular sequencing':ab,ti OR 'peptide sequence analysis':ab,ti OR 'peptide sequencing':ab,ti OR 'protein sequence analysis':ab,ti OR 'protein sequencing':ab,ti OR 'sequence analysis, protein':ab,ti OR 'Analysis, Sequence':ab,ti OR 'Analyses, Sequence':ab,ti OR 'Sequence Analyses':ab,ti OR 'Sequence Determinations':ab,ti OR 'Determination, Sequence':ab,ti OR 'Determinations, Sequence':ab,ti OR 'Sequence Determination':ab,ti OR 'sequence analysis':ab,ti OR 'DNA sequence determination':ab,ti OR 'DNA sequencing':ab,ti OR 'Analyses, DNA Sequence':ab,ti OR 'DNA Sequence Analyses':ab,ti OR 'Sequence Analys*, DNA':ab,ti OR 'Analysis, DNA Sequence':ab,ti OR 'DNA Sequence Analysis':ab,ti OR 'Sequence Determinations, DNA':ab,ti OR 'Determination, DNA Sequence':ab,ti OR 'Determinations, DNA Sequence':ab,ti OR 'DNA Sequence Determination':ab,ti OR 'DNA Sequence Determinations':ab,ti OR 'DNA Sequencing':ab,ti OR 'Sequencing, DNA':ab,ti OR 'Sequence Determination, DNA':ab,ti OR 'entire genome sequencing':ab,ti OR 'full genome sequencing':ab,ti OR 'WGS':ab,ti OR 'WGS analysis':ab,ti OR 'Genome Sequencing, Whole':ab,ti OR 'Sequencing, Whole Genome':ab,ti OR 'Complete Genome Sequencing':ab,ti OR 'Genome Sequencing, Complete':ab,ti OR 'Sequencing, Complete Genome':ab,ti OR 'whole genome sequencing':ab,ti OR 'prote-omics':ab,ti OR 'protein omics':ab,ti OR 'proteinomics':ab,ti OR 'Proteomics':ab,ti OR 'Peptidomics':ab,ti OR 'deoxyribonucleic acid methylation':ab,ti OR 'DNA hypermethylation':ab,ti OR 'DNA hypomethylation':ab,ti OR 'methylated deoxyribonucleic acid':ab,ti OR 'methylated dna':ab,ti OR 'DNA methylation*':ab,ti OR 'Methylation, DNA':ab,ti OR 'Methylations, DNA':ab,ti OR 'Analysis, DNA Mutational':ab,ti OR 'Analyses, DNA Mutational':ab,ti OR 'DNA Mutational Analyses':ab,ti OR 'Mutational Analyses, DNA':ab,ti OR 'Mutational Analysis, DNA':ab,ti OR 'community genomic':ab,ti OR 'community genomics':ab,ti OR 'eco-genomics':ab,ti OR 'ecogenomic':ab,ti OR 'ecogenomics':ab,ti OR 'ecological genomic':ab,ti OR 'ecological genomics':ab,ti OR 'environmental genomic':ab,ti OR 'environmental genomics':ab,ti OR 'meta-genomics':ab,ti OR 'Metagenomic':ab,ti OR 'Metagenomics':ab,ti OR 'Genomics, Environmental':ab,ti OR 'Genomics, Community':ab,ti OR 'Population Genomics':ab,ti OR 'Genomics, Population':ab,ti
42. 'imaging genomics'/exp
43. 'Radiogenomics'/exp
44. 'radiation oncology'/exp
45. 'Radiobiology'/exp
46. 'imaging radiogenomics':ab,ti OR 'neuroimaging genomics':ab,ti OR 'imaging genomics':ab,ti OR 'Genomics, Imaging':ab,ti OR 'Radiogenomic* ':ab,ti OR 'radiation genomics':ab,ti OR 'radio-genomics':ab,ti OR 'Oncology, Radiation':ab,ti OR 'Therapeutic Radiology':ab,ti OR 'Radiology, Therapeutic':ab,ti OR 'radiation oncology':ab,ti OR 'Biology, Radiation':ab,ti OR 'Radiation Biology':ab,ti OR 'radio biology':ab,ti OR 'radiobiology':ab,ti
47. #1 OR #2
48. #3 OR #4
49. #5 OR #6 OR #7
50. #8 OR #9
51. #10 OR #11 OR #12 OR #13 OR #14 OR #15 OR #16 OR #17 OR #18 OR #19 OR #20 OR #21 OR #22 OR #23 OR #24 OR #25 OR #26 OR #27 OR #28
52. #29 OR #30 OR #31 OR #32 OR #33
53. #35 AND #36
54. #37 OR #40
55. #38 AND #41
56. #39 OR #42
57. #34 AND #43
58. **PubMed search strategy (n = 2166)**

#1 Breast Neoplasms[MeSH Terms]

**#2** (((((((((((((((((((((((((((((((((((((((((((((((((((((((((((((((((((((((((((((((((((((((((((((((((((((((((((((((((((((((((((((((((((((((((((((((((((((((((((((((((((((((((((((((((((((Breast Neoplasm[Title/Abstract])) OR (Breast Neoplasms[Title/Abstract])) OR (Neoplasm, Breast[Title/Abstract])) OR (Neoplasms, Breast[Title/Abstract])) OR (Breast Tumors[Title/Abstract])) OR (Breast Tumor[Title/Abstract])) OR (Tumor, Breast[Title/Abstract])) OR (Tumors, Breast[Title/Abstract])) OR (Breast Cancer[Title/Abstract])) OR (Cancer, Breast[Title/Abstract])) OR (Cancer of Breast[Title/Abstract])) OR (Cancer of the Breast[Title/Abstract])) OR (Malignant Neoplasm of Breast[Title/Abstract])) OR (Breast Malignant Neoplasm[Title/Abstract])) OR (Breast Malignant Neoplasms[Title/Abstract])) OR (Malignant Tumor of Breast[Title/Abstract])) OR (Breast Malignant Tumor[Title/Abstract])) OR (Breast Malignant Tumors[Title/Abstract])) OR (Mammary Cancer[Title/Abstract])) OR (Cancer, Mammary[Title/Abstract])) OR (Cancers, Mammary[Title/Abstract])) OR (Mammary Cancers[Title/Abstract])) OR (Mammary Neoplasms, Human[Title/Abstract])) OR (Human Mammary Neoplasm[Title/Abstract])) OR (Human Mammary Neoplasms[Title/Abstract])) OR (Neoplasm, Human Mammary[Title/Abstract])) OR (Neoplasms, Human Mammary[Title/Abstract])) OR (Mammary Neoplasm, Human[Title/Abstract])) OR (Breast Carcinoma[Title/Abstract])) OR (Breast Carcinomas[Title/Abstract])) OR (Carcinoma, Breast[Title/Abstract])) OR (Carcinomas, Breast[Title/Abstract])) OR (Mammary Carcinoma, Human[Title/Abstract])) OR (Carcinoma, Human Mammary[Title/Abstract])) OR (Carcinomas, Human Mammary[Title/Abstract])) OR (Human Mammary Carcinomas[Title/Abstract])) OR (Mammary Carcinomas, Human[Title/Abstract])) OR (Human Mammary Carcinoma[Title/Abstract])) OR (bilateral breast neoplasm[Title/Abstract])) OR (bilateral breast tumor[Title/Abstract])) OR (bilateral breast tumour[Title/Abstract])) OR (breast gland tumor[Title/Abstract])) OR (breast gland tumour[Title/Abstract])) OR (breast mass[Title/Abstract])) OR (breast neoplasia[Title/Abstract])) OR (breast neoplasm[Title/Abstract])) OR (breast neoplasms[Title/Abstract])) OR (breast neoplasms, male[Title/Abstract])) OR (breast tumorigenesis[Title/Abstract])) OR (breast tumour[Title/Abstract])) OR (female breast neoplasm[Title/Abstract])) OR (female breast tumor[Title/Abstract])) OR (female breast tumour[Title/Abstract])) OR (male breast neoplasm[Title/Abstract])) OR (male breast tumor[Title/Abstract])) OR (male breast tumour[Title/Abstract])) OR (mamma tumor[Title/Abstract])) OR (mamma tumour[Title/Abstract])) OR (mammary gland neoplasia[Title/Abstract])) OR (mammary gland neoplasm[Title/Abstract])) OR (mammary gland tumor[Title/Abstract])) OR (mammary gland tumorigenesis[Title/Abstract])) OR (mammary gland tumour[Title/Abstract])) OR (mammary neoplasia[Title/Abstract])) OR (mammary neoplasm[Title/Abstract])) OR (mammary neoplasms[Title/Abstract])) OR (mammary tumor[Title/Abstract])) OR (mammary tumor cell[Title/Abstract])) OR (mammary tumorigenesis[Title/Abstract])) OR (mammary tumour[Title/Abstract])) OR (mammary tumour cell[Title/Abstract])) OR (mass in the breast[Title/Abstract])) OR (masses in the breast[Title/Abstract])) OR (neoplasia of the breast[Title/Abstract])) OR (neoplasm of the breast[Title/Abstract])) OR (neoplasm of the mammary gland[Title/Abstract])) OR (neoplastic breast[Title/Abstract])) OR (neoplastic mammary[Title/Abstract])) OR (neoplastic mammary gland[Title/Abstract])) OR (tumor of the breast[Title/Abstract])) OR (tumor of the female breast[Title/Abstract])) OR (tumor of the male breast[Title/Abstract])) OR (tumor of the mammary gland[Title/Abstract])) OR (tumorigenesis of the breast[Title/Abstract])) OR (tumorigenesis of the mammary gland[Title/Abstract])) OR (tumour of the male breast[Title/Abstract])) OR (unilateral breast neoplasm[Title/Abstract])) OR (unilateral breast neoplasms[Title/Abstract])) OR (unilateral breast tumor[Title/Abstract])) OR (breast gland cancer[Title/Abstract])) OR (breast gland neoplasm[Title/Abstract])) OR (breast malignancies[Title/Abstract])) OR (breast malignancy[Title/Abstract])) OR (breast tumor malignant[Title/Abstract])) OR (Ca breast[Title/Abstract])) OR (cancer in the mammary gland[Title/Abstract])) OR (cancer of the mammary gland[Title/Abstract])) OR (cancer, breast[Title/Abstract])) OR (malignancies of the breast[Title/Abstract])) OR (malignancy of the breast[Title/Abstract])) OR (malignant breast neoplasm[Title/Abstract])) OR (malignant breast tumor[Title/Abstract])) OR (malignant neoplasm of the breast[Title/Abstract])) OR (malignant tumor of the breast[Title/Abstract])) OR (mamma cancer[Title/Abstract])) OR (mammary gland cancer[Title/Abstract])) OR (mammary gland malignancy[Title/Abstract])) OR (mammary malignancies[Title/Abstract])) OR (mammary malignancy[Title/Abstract])) OR (breast carcinomata[Title/Abstract])) OR (breast carcinomatosis[Title/Abstract])) OR (carcinoma in the mammary gland[Title/Abstract])) OR (carcinoma of the breast[Title/Abstract])) OR (carcinoma of the mamma[Title/Abstract])) OR (carcinoma of the mammary gland[Title/Abstract])) OR (carcinoma, mammary[Title/Abstract])) OR (carcinomata of the breast[Title/Abstract])) OR (carcinomatous breast[Title/Abstract])) OR (carcinomatous mammary[Title/Abstract])) OR (carcinomatous mammary gland[Title/Abstract])) OR (human mammary carcinoma[Title/Abstract])) OR (mamma carcinoma[Title/Abstract])) OR (mammary carcinoma[Title/Abstract])) OR (mammary carcinomata[Title/Abstract])) OR (mammary carcinomatosis[Title/Abstract])) OR (mammary gland carcinoma[Title/Abstract])) OR (adenocarcinoma of the breast[Title/Abstract])) OR (adenocarcinoma of the mamma[Title/Abstract])) OR (adenocarcinoma of the mammary gland[Title/Abstract])) OR (mammary adenocarcinoma[Title/Abstract])) OR (breast adenocarcinoma[Title/Abstract])) OR (Carcinoma, Ductal, Breast[Title/Abstract])) OR (Carcinoma, Infiltrating Duct[Title/Abstract])) OR (Carcinomas, Infiltrating Duct[Title/Abstract])) OR (Carcinoma, Invasive Ductal, Breast[Title/Abstract])) OR (Invasive Ductal Carcinoma, Breast[Title/Abstract])) OR (Carcinoma, Mammary Ductal[Title/Abstract])) OR (Carcinomas, Mammary Ductal[Title/Abstract])) OR (Mammary Ductal Carcinomas[Title/Abstract])) OR (Mammary Ductal Carcinoma[Title/Abstract])) OR (breast duct carcinoma[Title/Abstract])) OR (breast ductal adenocarcinoma[Title/Abstract])) OR (carcinoma, ductal, breast[Title/Abstract])) OR (ductal adenocarcinoma of the breast[Title/Abstract])) OR (ductal breast adenocarcinoma[Title/Abstract])) OR (ductal breast carcinoma[Title/Abstract])) OR (ductal carcinoma of the breast[Title/Abstract])) OR (ductal mammary carcinoma[Title/Abstract])) OR (mammary duct carcinoma[Title/Abstract])) OR (breast ductal carcinoma[Title/Abstract])) OR (breast invasive ductal carcinoma[Title/Abstract])) OR (ductal invasive breast carcinoma[Title/Abstract])) OR (infiltrating duct carcinoma of breast[Title/Abstract])) OR (infiltrating ductal breast cancer[Title/Abstract])) OR (infiltrating ductal breast carcinoma[Title/Abstract])) OR (infiltrating ductal carcinoma of the breast[Title/Abstract])) OR (invasive ductal breast cancer[Title/Abstract])) OR (invasive ductal carcinoma of the breast[Title/Abstract])) OR (invasive ductal breast carcinoma[Title/Abstract])) OR (Carcinoma, Lobular[Title/Abstract])) OR (Carcinomas, Lobular[Title/Abstract])) OR (Lobular Carcinoma[Title/Abstract])) OR (Lobular Carcinomas[Title/Abstract])) OR (carcinoma, lobular[Title/Abstract])) OR (lobular adenocarcinoma[Title/Abstract])) OR (lobular adenocarcinoma of the breast[Title/Abstract])) OR (lobular breast adenocarcinoma[Title/Abstract])) OR (lobular breast carcinoma[Title/Abstract])) OR (lobular cancer[Title/Abstract])) OR (lobular cancer of the breast[Title/Abstract])) OR (lobular carcinoma of the breast[Title/Abstract])) OR (lobular carcinoma[Title/Abstract])) OR (infiltrating lobular breast cancer[Title/Abstract])) OR (infiltrating lobular breast carcinoma[Title/Abstract])) OR (infiltrating lobular carcinoma[Title/Abstract])) OR (infiltrative lobular carcinoma[Title/Abstract])) OR (invasive lobular breast cancer[Title/Abstract])) OR (invasive lobular cancer of the breast[Title/Abstract])) OR (invasive lobular carcinoma[Title/Abstract])) OR (invasive lobular carcinoma of the breast[Title/Abstract])) OR (invasive lobular breast carcinoma[Title/Abstract])

#3 artificial intelligence[Mesh Terms]

#4 (((((((((((((((((((((((((((((((((((((((((((((((((((((((((((((((((((((((computer assisted diagnosis[Title/Abstract])) OR (automatic diagnosis[Title/Abstract])) OR (computer diagnosis[Title/Abstract])) OR (computer-assisted diagnosis[Title/Abstract])) OR (computer-assisted image interpretation[Title/Abstract])) OR (computer-assisted radiographic image interpretation[Title/Abstract])) OR (diagnosis, computer[Title/Abstract])) OR (diagnosis, computer-assisted[Title/Abstract])) OR (image interpretation, computer-assisted[Title/Abstract])) OR (radiographic image interpretation, computer-assisted[Title/Abstract])) OR (Intelligence, Artificial[Title/Abstract])) OR (Computer Reasoning[Title/Abstract])) OR (Reasoning, Computer[Title/Abstract])) OR (AI[Title/Abstract])) OR (Machine Intelligence[Title/Abstract])) OR (Intelligence, Machine[Title/Abstract])) OR (Computational Intelligence[Title/Abstract])) OR (Intelligence, Computational[Title/Abstract])) OR (Computer Vision Systems[Title/Abstract])) OR (Computer Vision System[Title/Abstract])) OR (System, Computer Vision[Title/Abstract])) OR (Systems, Computer Vision[Title/Abstract])) OR (Vision System, Computer[Title/Abstract])) OR (Vision Systems, Computer[Title/Abstract])) OR (artificial intelligence[Title/Abstract])) OR (machine learning[Title/Abstract])) OR (learning machine[Title/Abstract])) OR (learning machines[Title/Abstract])) OR (Learning, Machine[Title/Abstract])) OR (Transfer Learning[Title/Abstract])) OR (Learning, Transfer[Title/Abstract])) OR (artificial neural network[Title/Abstract])) OR (algorithmic neural network[Title/Abstract])) OR (ANN[Title/Abstract])) OR (ANN analysis[Title/Abstract])) OR (ANN approach[Title/Abstract])) OR (ANN method[Title/Abstract])) OR (ANN methodology[Title/Abstract])) OR (ANN methods[Title/Abstract])) OR (ANN model[Title/Abstract])) OR (ANN modeling[Title/Abstract])) OR (ANN modelling[Title/Abstract])) OR (ANN models[Title/Abstract])) OR (ANN output[Title/Abstract])) OR (ANN technique[Title/Abstract])) OR (ANN techniques[Title/Abstract])) OR (ANN training[Title/Abstract])) OR (ANNs[Title/Abstract])) OR (artificial neural networks[Title/Abstract])) OR (artificial NN[Title/Abstract])) OR (artificial NNs[Title/Abstract])) OR (computational neural network[Title/Abstract])) OR (computer neural network[Title/Abstract])) OR (computer neural networks[Title/Abstract])) OR (computerized neural network[Title/Abstract])) OR (connectionist model[Title/Abstract])) OR (connectionist network[Title/Abstract])) OR (connectionist neural network[Title/Abstract])) OR (connectionist system[Title/Abstract])) OR (mathematical neural network[Title/Abstract])) OR (neural network[Title/Abstract])) OR (neural network algorithm[Title/Abstract])) OR (neural network model[Title/Abstract])) OR (neural networks [Title/Abstract])) OR (neural networks, computer[Title/Abstract])) OR (deep learning[Title/Abstract])) OR (deep machine learning[Title/Abstract])) OR (deep ML[Title/Abstract])) OR (hierarchical learning[Title/Abstract])) OR (Learning, Deep[Title/Abstract])) OR (Learning, Hierarchical[Title/Abstract])

#5 (Diagnostic Imaging[MeSH Terms]) AND (Radiology[MeSH Terms])

#6 (((((((((((((((((((((((((((((((((((((((((((((((((((((((((((((((((((((((((((((((((((((((((((((((((((((((((((((((((((((((((((((((((((((((((((((((((((((((((((((((((((((((((((((((((((((((((((((((((((((((((((((((((((((((((((((((((((((((((((((((((((((((((((((((imaging, diagnostic[Title/Abstract])) OR (imaging, medical[Title/Abstract])) OR (medical imaging[Title/Abstract])) OR (ophthalmo diaphanoscopy[Title/Abstract])) OR (diagnostic imaging[Title/Abstract])) OR (imaging, magnetization transfer[Title/Abstract])) OR (magnetic resonance imaging[Title/Abstract])) OR (magnetic resonance tomography[Title/Abstract])) OR (magnetization transfer imaging[Title/Abstract])) OR (mr imaging[Title/Abstract])) OR (MRI[Title/Abstract])) OR (nuclear magnetic resonance imaging[Title/Abstract])) OR (Imaging, Magnetic Resonance[Title/Abstract])) OR (NMR Imaging[Title/Abstract])) OR (Imaging, NMR[Title/Abstract])) OR (Zeugmatography[Title/Abstract])) OR (Tomography, MR[Title/Abstract])) OR (Steady-State Free Precession MRI[Title/Abstract])) OR (Steady State Free Precession MRI[Title/Abstract])) OR (NMR Tomography[Title/Abstract])) OR (Tomography, NMR[Title/Abstract])) OR (MR Tomography[Title/Abstract])) OR (Tomography, Proton Spin[Title/Abstract])) OR (Proton Spin Tomography[Title/Abstract])) OR (Magnetization Transfer Contrast Imaging[Title/Abstract])) OR (fMRI[Title/Abstract])) OR (Magnetic Resonance Imaging, Functional[Title/Abstract])) OR (MRI, Functional[Title/Abstract])) OR (Functional MRI[Title/Abstract])) OR (Functional MRIs[Title/Abstract])) OR (MRIs, Functional[Title/Abstract])) OR (Functional Magnetic Resonance Imaging[Title/Abstract])) OR (MRI Scans[Title/Abstract])) OR (MRI Scan[Title/Abstract])) OR (Scan, MRI[Title/Abstract])) OR (Scans, MRI[Title/Abstract])) OR (Imaging, Chemical Shift[Title/Abstract])) OR (Chemical Shift Imagings[Title/Abstract])) OR (Imagings, Chemical Shift[Title/Abstract])) OR (Shift Imaging, Chemical[Title/Abstract])) OR (Shift Imagings, Chemical[Title/Abstract])) OR (Chemical Shift Imaging[Title/Abstract])) OR (Spin Echo Imaging[Title/Abstract])) OR (Echo Imaging, Spin[Title/Abstract])) OR (Echo Imagings, Spin[Title/Abstract])) OR (Imaging, Spin Echo[Title/Abstract])) OR (Imagings, Spin Echo[Title/Abstract])) OR (Spin Echo Imagings[Title/Abstract])) OR (Magnetic Resonance Image[Title/Abstract])) OR (Image, Magnetic Resonance[Title/Abstract])) OR (Magnetic Resonance Images[Title/Abstract])) OR (Resonance Image, Magnetic[Title/Abstract])) OR (breast MRI[Title/Abstract])) OR (magnetic resonance breast imaging[Title/Abstract])) OR (magnetic resonance mammography[Title/Abstract])) OR (breast magnetic resonance imaging[Title/Abstract])) OR (Mammography[Title/Abstract])) OR (mamilloscopy[Title/Abstract])) OR (mammilloscopy[Title/Abstract])) OR (mammo-graphy[Title/Abstract])) OR (mammogram[Title/Abstract])) OR (mastography Mammographies[Title/Abstract])) OR (Digital Breast Tomosynthesis[Title/Abstract])) OR (Breast Tomosyntheses, Digital[Title/Abstract])) OR (Breast Tomosynthesis, Digital[Title/Abstract])) OR (Digital Breast Tomosyntheses[Title/Abstract])) OR (X-ray Breast Tomosynthesis[Title/Abstract])) OR (Breast Tomosyntheses, X-ray[Title/Abstract])) OR (Breast Tomosynthesis, X-ray[Title/Abstract])) OR (X-ray Breast Tomosyntheses[Title/Abstract])) OR (X ray Breast Tomosynthesis[Title/Abstract])) OR (3D-Mammography[Title/Abstract])) OR (3D-Mammographies[Title/Abstract])) OR (3D Mammography[Title/Abstract])) OR (Digital Mammography[Title/Abstract])) OR (Digital Mammographies[Title/Abstract])) OR (Mammographies, Digital[Title/Abstract])) OR (Mammography, Digital[Title/Abstract])) OR (diagnostic radiology[Title/Abstract])) OR (radio diagnosis[Title/Abstract])) OR (radiographic examination[Title/Abstract])) OR (radiologic diagnosis[Title/Abstract])) OR (radiologic examination[Title/Abstract])) OR (radiological diagnosis[Title/Abstract])) OR (radiological examination[Title/Abstract])) OR (radiology, diagnostic[Title/Abstract])) OR (roentgen diagnosis[Title/Abstract])) OR (roentgen diagnostics[Title/Abstract])) OR (roentgen examination[Title/Abstract])) OR (roentgen screening[Title/Abstract])) OR (roentgenologic diagnosis[Title/Abstract])) OR (roentgenologic diagnostics[Title/Abstract])) OR (roentgenologic examination[Title/Abstract])) OR (roentgenologic screening[Title/Abstract])) OR (roentgenological diagnosis[Title/Abstract])) OR (roentgenological diagnostics[Title/Abstract])) OR (roentgenological examination[Title/Abstract])) OR (roentgenological screening[Title/Abstract])) OR (rontgen diagnosis[Title/Abstract])) OR (rontgen examination[Title/Abstract])) OR (rontgenologic diagnosis[Title/Abstract])) OR (rontgenologic examination[Title/Abstract])) OR (rontgenological diagnosis[Title/Abstract])) OR (rontgenological examination[Title/Abstract])) OR (X ray diagnosis[Title/Abstract])) OR (X ray examination[Title/Abstract])) OR (radiodiagnosis[Title/Abstract])) OR (radiologic*[Title/Abstract])) OR (roentgenological*[Title/Abstract])) OR (roentgenology[Title/Abstract])) OR (radiology[Title/Abstract])) OR (Radiography[Title/Abstract])) OR (Diagnostic X-Ray[Title/Abstract])) OR (Diagnostic X Ray[Title/Abstract])) OR (Diagnostic X-Rays[Title/Abstract])) OR (X-Rays, Diagnostic[Title/Abstract])) OR (X-Ray, Diagnostic[Title/Abstract])) OR (X Ray, Diagnostic[Title/Abstract])) OR (Diagnostic X-Ray Radiology[Title/Abstract])) OR (Diagnostic X Ray Radiology[Title/Abstract])) OR (X-Ray Radiology, Diagnostic[Title/Abstract])) OR (X Ray Radiology, Diagnostic[Title/Abstract])) OR (Radiology, Diagnostic X-Ray[Title/Abstract])) OR (Radiology, Diagnostic X Ray[Title/Abstract])) OR (dual-energy scanned projection radiography[Title/Abstract])) OR (electroradiography[Title/Abstract])) OR (pneumoradiography[Title/Abstract])) OR (radiogram[Title/Abstract])) OR (radiographic method[Title/Abstract])) OR (radiography, dual-energy scanned projection[Title/Abstract])) OR (radioimaging[Title/Abstract])) OR (radiophotography[Title/Abstract])) OR (roentgen photography[Title/Abstract])) OR (roentgenography[Title/Abstract])) OR (roentgenoscopy[Title/Abstract])) OR (rontgenography[Title/Abstract])) OR (X ray imaging[Title/Abstract])) OR (x ray photography [Title/Abstract])) OR (computer assisted positron emission tomography[Title/Abstract])) OR (positron emission computed tomography[Title/Abstract])) OR (positron emission tomography computed tomography[Title/Abstract])) OR (positron emission tomography, computer assisted[Title/Abstract])) OR (positron-emission tomography and computed tomography[Title/Abstract])) OR (positron emission tomography-computed tomography[Title/Abstract])) OR (PET-CT Scan[Title/Abstract])) OR (PET-CT Scans[Title/Abstract])) OR (Scan, PET-CT[Title/Abstract])) OR (Scans, PET-CT[Title/Abstract])) OR (PET CT Scan[Title/Abstract])) OR (CT Scan, PET[Title/Abstract])) OR (CT Scans, PET[Title/Abstract])) OR (PET CT Scans[Title/Abstract])) OR (Scan, PET CT[Title/Abstract])) OR (Scans, PET CT[Title/Abstract])) OR (PET-CT[Title/Abstract])) OR (CT PET[Title/Abstract])) OR (CT PET Scan[Title/Abstract])) OR (CT PET Scans[Title/Abstract])) OR (PET Scan, CT[Title/Abstract])) OR (PET Scans, CT[Title/Abstract])) OR (Scan, CT PET[Title/Abstract])) OR (Scans, CT PET[Title/Abstract])) OR (p.e.t.[Title/Abstract])) OR (PET scanning[Title/Abstract])) OR (positron emission tomographic scan[Title/Abstract])) OR (positron emission tomographic scanning[Title/Abstract])) OR (positron tomography[Title/Abstract])) OR (positron-emission tomography[Title/Abstract])) OR (tomography, positron[Title/Abstract])) OR (positron emission tomography[Title/Abstract])) OR (PET Imaging[Title/Abstract])) OR (Imaging, PET[Title/Abstract])) OR (PET Imagings[Title/Abstract])) OR (Positron-Emission Tomography Imaging[Title/Abstract])) OR (Imaging, Positron-Emission Tomography[Title/Abstract])) OR (Positron Emission Tomography Imaging[Title/Abstract])) OR (Positron-Emission Tomography Imagings[Title/Abstract])) OR (Tomography Imaging, Positron-Emission[Title/Abstract])) OR (PET Scan[Title/Abstract])) OR (PET Scans[Title/Abstract])) OR (Scan, PET[Title/Abstract])) OR (Tomography, Positron-Emission[Title/Abstract])) OR (Tomography, Positron Emission[Title/Abstract])) OR (diagnostic ultrasonic examination[Title/Abstract])) OR (diagnostic ultrasonic imaging[Title/Abstract])) OR (diagnostic ultrasonic method[Title/Abstract])) OR (doptone[Title/Abstract])) OR (duplex echography[Title/Abstract])) OR (echogram[Title/Abstract])) OR (echographic evaluation[Title/Abstract])) OR (echoscopy[Title/Abstract])) OR (echosound[Title/Abstract])) OR (high resolution echography[Title/Abstract])) OR (scanning, ultrasonic[Title/Abstract])) OR (sonogram[Title/Abstract])) OR (sonographic examination[Title/Abstract])) OR (sonographic screening[Title/Abstract])) OR (sonography[Title/Abstract])) OR (ultrasonic detection[Title/Abstract])) OR (ultrasonic echo[Title/Abstract])) OR (ultrasonic examination[Title/Abstract])) OR (ultrasonic scanning[Title/Abstract])) OR (ultrasonic scintillation[Title/Abstract])) OR (ultrasonogram[Title/Abstract])) OR (ultrasonographic examination[Title/Abstract])) OR (ultrasonographic screening[Title/Abstract])) OR (ultrasonography[Title/Abstract])) OR (ultrasound diagnosis[Title/Abstract])) OR (ultrasound scanning [Title/Abstract])) OR (Echotomography[Title/Abstract])) OR (Ultrasonic Imaging[Title/Abstract])) OR (Imaging, Ultrasonic[Title/Abstract])) OR (Sonography, Medical[Title/Abstract])) OR (Medical Sonography[Title/Abstract])) OR (Ultrasound Imaging[Title/Abstract])) OR (Imagings, Ultrasound[Title/Abstract])) OR (Imaging, Ultrasound[Title/Abstract])) OR (Ultrasonographic Imaging[Title/Abstract])) OR (Imagings, Ultrasonographic[Title/Abstract])) OR (Imaging, Ultrasonographic[Title/Abstract])) OR (Ultrasonographic Imagings[Title/Abstract])) OR (Echography[Title/Abstract])) OR (Diagnostic Ultrasound[Title/Abstract])) OR (Diagnostic Ultrasounds[Title/Abstract])) OR (Ultrasound, Diagnostic[Title/Abstract])) OR (Ultrasounds, Diagnostic[Title/Abstract])) OR (Echotomography, Computer[Title/Abstract])) OR (Computer Echotomography[Title/Abstract])) OR (Tomography, Ultrasonic[Title/Abstract])) OR (Ultrasonic Tomography[Title/Abstract])) OR (Diagnosis, Ultrasonic[Title/Abstract])) OR (Diagnoses, Ultrasonic[Title/Abstract])) OR (Ultrasonic Diagnoses[Title/Abstract])) OR (Ultrasonic Diagnosis[Title/Abstract])) OR (Echomammography[Title/Abstract])) OR (breast echography[Title/Abstract])) OR (breast ultrasound[Title/Abstract])) OR (echography, breast[Title/Abstract])) OR (sonography, breast[Title/Abstract])) OR (Ultrasonography, Mammary[Title/Abstract])) OR (Ultrasonic Mammography[Title/Abstract])) OR (Mammographies, Ultrasonic[Title/Abstract])) OR (Ultrasonic Mammographies[Title/Abstract])) OR (Mammography, Ultrasonic[Title/Abstract])) OR (Mammography, Ultrasound[Title/Abstract])) OR (Mammographies, Ultrasound[Title/Abstract])) OR (Ultrasound Mammographies[Title/Abstract])) OR (Ultrasound Mammography[Title/Abstract])) OR (Mammary Ultrasonography[Title/Abstract])) OR (Mammary Ultrasonographies[Title/Abstract])) OR (Ultrasonographies, Mammary[Title/Abstract])) OR (Ultrasonography, Breast[Title/Abstract])) OR (Breast Ultrasonography[Title/Abstract])) OR (Breast Ultrasonographies[Title/Abstract])) OR (Ultrasonographies, Breast[Title/Abstract])

#7 Image Processing, Computer-Assisted[MeSH Terms]

#8 (((((((((((((((((((((((((((((((((((((((((((((((computer-assisted image processing[Title/Abstract])) OR (processing, image[Title/Abstract])) OR (image processing[Title/Abstract])) OR (Image Processing, Computer Assisted[Title/Abstract])) OR (Computer-Assisted Image Processing[Title/Abstract])) OR (Computer Assisted Image Processing[Title/Abstract])) OR (Image Analysis, Computer-Assisted[Title/Abstract])) OR (Computer-Assisted Image Analyses[Title/Abstract])) OR (Image Analyses, Computer-Assisted[Title/Abstract])) OR (Image Analysis, Computer Assisted[Title/Abstract])) OR (Analysis, Computer-Assisted Image[Title/Abstract])) OR (Computer-Assisted Image Analysis[Title/Abstract])) OR (Computer Assisted Image Analysis[Title/Abstract])) OR (Image Reconstruction[Title/Abstract])) OR (Image Reconstructions[Title/Abstract])) OR (Reconstruction, Image[Title/Abstract])) OR (Reconstructions, Image[Title/Abstract])) OR (Digital Image Processing[Title/Abstract])) OR (Image Processing, Digital[Title/Abstract])) OR (Processing, Digital Image[Title/Abstract])) OR (Processings, Digital Image[Title/Abstract])) OR (Medical Image Processing[Title/Abstract])) OR (Image Processing, Medical[Title/Abstract])) OR (Image Processings, Medical[Title/Abstract])) OR (Medical Image Processings[Title/Abstract])) OR (Processing, Medical Image[Title/Abstract])) OR (Processings, Medical Image[Title/Abstract])) OR (Biomedical Image Processing[Title/Abstract])) OR (Image Processing, Biomedical[Title/Abstract])) OR (Processing, Biomedical Image[Title/Abstract])) OR (Image Interpretation, Computer-Assisted[Title/Abstract])) OR (Computer-Assisted Image Interpretation[Title/Abstract])) OR (Computer-Assisted Image Interpretations[Title/Abstract])) OR (Image Interpretations, Computer-Assisted[Title/Abstract])) OR (Interpretation, Computer-Assisted Image[Title/Abstract])) OR (Interpretations, Computer-Assisted Image[Title/Abstract])) OR (Image Interpretation, Computer Assisted[Title/Abstract])) OR (computer assisted diagnosis[Title/Abstract])) OR (automatic diagnosis[Title/Abstract])) OR (computer diagnosis[Title/Abstract])) OR (computer-assisted diagnosis[Title/Abstract])) OR (computer-assisted radiographic image interpretation[Title/Abstract])) OR (diagnosis, computer[Title/Abstract])) OR (diagnosis, computer-assisted[Title/Abstract])) OR (radiographic image interpretation, computer-assisted[Title/Abstract])) OR (computer assisted diagnosis[Title/Abstract])) OR (Radiomi*[Title/Abstract])

#9 ((((((((((((((((Genomics[MeSH Terms]) OR (Proteomics[MeSH Terms])) OR (Gene Ontology[MeSH Terms])) OR (Gene Expression[MeSH Terms])) OR (Gene Expression Regulation, Neoplastic[MeSH Terms])) OR (Transcriptome[MeSH Terms])) OR (Gene Expression Profiling[MeSH Terms])) OR (Sequence Analysis, DNA[MeSH Terms])) OR (Sequence Analysis, Protein[MeSH Terms])) OR (Sequence Analysis, RNA[MeSH Terms])) OR (RNA, Messenger[MeSH Terms])) OR (Genetic Code[MeSH Terms])) OR (Genome[MeSH Terms])) OR (DNA Methylation[MeSH Terms])) OR (DNA Mutational Analysis[MeSH Terms])) OR (Whole Genome Sequencing[MeSH Terms])) OR (Metagenomics[MeSH Terms])

#10 (((((((((((((((((((((((((((((((((((((((((((((((((((((((((((((((((((((((((((((((((((((((((((((((((((((((((((((((((((((((((((((((((((((((((((((((((((((((((((((((((((((((((((((((((((((((((((((((((((((((((((((((((((((((((((((((Comparative Genomics[Title/Abstract])) OR (Genomics, Comparative[Title/Abstract])) OR (Structural Genomics[Title/Abstract])) OR (Genomics, Structural[Title/Abstract])) OR (Functional Genomics[Title/Abstract])) OR (Genomics, Functional[Title/Abstract])) OR (genome components[Title/Abstract])) OR (genome, helminth[Title/Abstract])) OR (genome, protozoan[Title/Abstract])) OR (genome, protozoon[Title/Abstract])) OR (protozoan genome[Title/Abstract])) OR (protozoon genome[Title/Abstract])) OR (Genome*[Title/Abstract])) OR (expression*, gene[Title/Abstract])) OR (genetic expression[Title/Abstract])) OR (genome expression[Title/Abstract])) OR (gene expression*[Title/Abstract])) OR (dosage compensation[Title/Abstract])) OR (dosage compensation, genetic[Title/Abstract])) OR (gene dosage compensation[Title/Abstract])) OR (gene expression regulation, archaeal[Title/Abstract])) OR (gene expression regulation, bacterial[Title/Abstract])) OR (gene expression regulation, developmental[Title/Abstract])) OR (gene expression regulation, enzymologic[Title/Abstract])) OR (gene expression regulation, fungal[Title/Abstract])) OR (gene expression regulation, leukaemic[Title/Abstract])) OR (gene expression regulation, leukemic[Title/Abstract])) OR (gene expression regulation, neoplastic[Title/Abstract])) OR (gene expression regulation, plant[Title/Abstract])) OR (gene expression regulation, viral[Title/Abstract])) OR (genetic dosage compensation[Title/Abstract])) OR (Regulation, Gene Action[Title/Abstract])) OR (Gene Action Regulation[Title/Abstract])) OR (Expression Regulation, Gene[Title/Abstract])) OR (Regulation, Gene Expression[Title/Abstract])) OR (Regulation of Gene Expression[Title/Abstract])) OR (gene expression regulation[Title/Abstract])) OR (epigenomic*[Title/Abstract])) OR (Epigenetic*[Title/Abstract])) OR (Gene Ontologies[Title/Abstract])) OR (Ontologies, Gene[Title/Abstract])) OR (Ontology, Gene[Title/Abstract])) OR (Gene Ontology Project[Title/Abstract])) OR (Gene Ontology Projects[Title/Abstract])) OR (Ontology Project, Gene[Title/Abstract])) OR (Ontology Projects, Gene[Title/Abstract])) OR (Project, Gene Ontology[Title/Abstract])) OR (Projects, Gene Ontology[Title/Abstract])) OR (gene ontology[Title/Abstract])) OR (gene expression analysis[Title/Abstract])) OR (gene expression profile[Title/Abstract])) OR (gene product profiling[Title/Abstract])) OR (gene expression profiling*[Title/Abstract])) OR (Profiling, Gene Expression[Title/Abstract])) OR (Profilings, Gene Expression[Title/Abstract])) OR (Gene Expression Monitoring[Title/Abstract])) OR (Gene Expression Monitorings[Title/Abstract])) OR (Monitoring, Gene Expression[Title/Abstract])) OR (Monitorings, Gene Expression[Title/Abstract])) OR (Gene Expression Pattern Analysis[Title/Abstract])) OR (Transcript Expression Analysis[Title/Abstract])) OR (Analyses, Transcript Expression[Title/Abstract])) OR (Analysis, Transcript Expression[Title/Abstract])) OR (Transcript Expression Analyses[Title/Abstract])) OR (Transcriptome Analysis[Title/Abstract])) OR (Analyses, Transcriptome[Title/Abstract])) OR (Analysis, Transcriptome[Title/Abstract])) OR (Transcriptome Analyses[Title/Abstract])) OR (Transcriptome Profiling[Title/Abstract])) OR (Profilings, Transcriptome[Title/Abstract])) OR (Profiling, Transcriptome[Title/Abstract])) OR (Transcriptome Profilings[Title/Abstract])) OR (Transcriptomics[Title/Abstract])) OR (Gene Expression Analysis[Title/Abstract])) OR (Analyses, Gene Expression[Title/Abstract])) OR (Analysis, Gene Expression[Title/Abstract])) OR (Expression Analyses, Gene[Title/Abstract])) OR (Expression Analysis, Gene[Title/Abstract])) OR (Gene Expression Analyses[Title/Abstract])) OR (mRNA Differential Display[Title/Abstract])) OR (Differential Display, mRNA[Title/Abstract])) OR (Differential Displays, mRNA[Title/Abstract])) OR (mRNA Differential Displays[Title/Abstract])) OR (code, genetic[Title/Abstract])) OR (deoxyribonucleic acid code[Title/Abstract])) OR (dna code[Title/Abstract])) OR (genetic coding[Title/Abstract])) OR (genetic code*[Title/Abstract])) OR (codes, genetic[Title/Abstract])) OR (transcriptome*[Title/Abstract])) OR (Gene Expression Profiles[Title/Abstract])) OR (Expression Profile, Gene[Title/Abstract])) OR (Expression Profiles, Gene[Title/Abstract])) OR (Gene Expression Profile[Title/Abstract])) OR (Profile, Gene Expression[Title/Abstract])) OR (Profiles, Gene Expression[Title/Abstract])) OR (Gene Expression Signatures[Title/Abstract])) OR (Expression Signature, Gene[Title/Abstract])) OR (Expression Signatures, Gene[Title/Abstract])) OR (Gene Expression Signature[Title/Abstract])) OR (Signature, Gene Expression[Title/Abstract])) OR (Signatures, Gene Expression[Title/Abstract])) OR (Transcriptome Profiles[Title/Abstract])) OR (Profiles, Transcriptome[Title/Abstract])) OR (Profile, Transcriptome[Title/Abstract])) OR (Transcriptome Profile[Title/Abstract])) OR (b rna[Title/Abstract])) OR (brna[Title/Abstract])) OR (messenger ribonucleic acid[Title/Abstract])) OR (ribonucleic acid, messenger[Title/Abstract])) OR (rna, messenger[Title/Abstract])) OR (RNA, messenger, stored[Title/Abstract])) OR (rna, messenger[Title/Abstract])) OR (Messenger RNA[Title/Abstract])) OR (mRNA[Title/Abstract])) OR (mRNA, Non-Polyadenylated[Title/Abstract])) OR (mRNA, Non Polyadenylated[Title/Abstract])) OR (Non-Polyadenylated mRNA[Title/Abstract])) OR (Non Polyadenylated mRNA[Title/Abstract])) OR (mRNA, Polyadenylated[Title/Abstract])) OR (Polyadenylated mRNA[Title/Abstract])) OR (Messenger RNA, Polyadenylated[Title/Abstract])) OR (Polyadenylated Messenger RNA[Title/Abstract])) OR (RNA, Polyadenylated Messenger[Title/Abstract])) OR (RNA, Messenger, Polyadenylated[Title/Abstract])) OR (RNA, Polyadenylated[Title/Abstract])) OR (Polyadenylated RNA[Title/Abstract])) OR (RNA seq[Title/Abstract])) OR (RNA-Seq[Title/Abstract])) OR (RNAseq[Title/Abstract])) OR (sequence analysis, RNA[Title/Abstract])) OR (Analyses, RNA Sequence[Title/Abstract])) OR (RNA Sequence Analyses[Title/Abstract])) OR (Sequence Analyses, RNA[Title/Abstract])) OR (Analysis, RNA Sequence[Title/Abstract])) OR (RNA Sequence Analysis[Title/Abstract])) OR (Sequence Determinations, RNA[Title/Abstract])) OR (Determination, RNA Sequence[Title/Abstract])) OR (Determinations, RNA Sequence[Title/Abstract])) OR (RNA Sequence Determination[Title/Abstract])) OR (RNA Sequence Determinations[Title/Abstract])) OR (RNA Sequencing[Title/Abstract])) OR (Sequencing, RNA[Title/Abstract])) OR (Sequence Determination, RNA[Title/Abstract])) OR (amino acid sequence analysis[Title/Abstract])) OR (amino acid sequencing[Title/Abstract])) OR (aminoacid sequence analysis[Title/Abstract])) OR (genetic sequencing[Title/Abstract])) OR (genomic sequencing[Title/Abstract])) OR (macromolecular sequencing[Title/Abstract])) OR (molecular sequencing[Title/Abstract])) OR (peptide sequence analysis[Title/Abstract])) OR (peptide sequencing[Title/Abstract])) OR (protein sequence analysis[Title/Abstract])) OR (protein sequencing[Title/Abstract])) OR (sequence analysis, protein[Title/Abstract])) OR (Analysis, Sequence[Title/Abstract])) OR (Analyses, Sequence[Title/Abstract])) OR (Sequence Analyses[Title/Abstract])) OR (Sequence Determinations[Title/Abstract])) OR (Determination, Sequence[Title/Abstract])) OR (Determinations, Sequence[Title/Abstract])) OR (Sequence Determination[Title/Abstract])) OR (sequence analysis[Title/Abstract])) OR (DNA sequence determination[Title/Abstract])) OR (DNA sequencing[Title/Abstract])) OR (Analyses, DNA Sequence[Title/Abstract])) OR (DNA Sequence Analyses[Title/Abstract])) OR (Sequence Analys*, DNA[Title/Abstract])) OR (Analysis, DNA Sequence[Title/Abstract])) OR (DNA Sequence Analysis[Title/Abstract])) OR (Sequence Determinations, DNA[Title/Abstract])) OR (Determination, DNA Sequence[Title/Abstract])) OR (Determinations, DNA Sequence[Title/Abstract])) OR (DNA Sequence Determination[Title/Abstract])) OR (DNA Sequence Determinations[Title/Abstract])) OR (DNA Sequencing[Title/Abstract])) OR (Sequencing, DNA[Title/Abstract])) OR (Sequence Determination, DNA[Title/Abstract])) OR (entire genome sequencing[Title/Abstract])) OR (full genome sequencing[Title/Abstract])) OR (WGS[Title/Abstract])) OR (WGS analysis[Title/Abstract])) OR (Genome Sequencing, Whole[Title/Abstract])) OR (Sequencing, Whole Genome[Title/Abstract])) OR (Complete Genome Sequencing[Title/Abstract])) OR (Genome Sequencing, Complete[Title/Abstract])) OR (Sequencing, Complete Genome[Title/Abstract])) OR (whole genome sequencing[Title/Abstract])) OR (prote-omics[Title/Abstract])) OR (protein omics[Title/Abstract])) OR (proteinomics[Title/Abstract])) OR (Proteomics[Title/Abstract])) OR (Peptidomics[Title/Abstract])) OR (deoxyribonucleic acid methylation[Title/Abstract])) OR (DNA hypermethylation[Title/Abstract])) OR (DNA hypomethylation[Title/Abstract])) OR (methylated deoxyribonucleic acid[Title/Abstract])) OR (methylated dna[Title/Abstract])) OR (DNA methylation*[Title/Abstract])) OR (Methylation, DNA[Title/Abstract])) OR (Methylations, DNA[Title/Abstract])) OR (Analysis, DNA Mutational[Title/Abstract])) OR (Analyses, DNA Mutational[Title/Abstract])) OR (DNA Mutational Analyses[Title/Abstract])) OR (Mutational Analyses, DNA[Title/Abstract])) OR (Mutational Analysis, DNA[Title/Abstract])) OR (community genomic[Title/Abstract])) OR (community genomics[Title/Abstract])) OR (eco-genomics[Title/Abstract])) OR (ecogenomic[Title/Abstract])) OR (ecogenomics[Title/Abstract])) OR (ecological genomic[Title/Abstract])) OR (ecological genomics[Title/Abstract])) OR (environmental genomic[Title/Abstract])) OR (environmental genomics[Title/Abstract])) OR (meta-genomics[Title/Abstract])) OR (Metagenomic[Title/Abstract])) OR (Metagenomics[Title/Abstract])) OR (Genomics, Environmental[Title/Abstract])) OR (Genomics, Community[Title/Abstract])) OR (Population Genomics[Title/Abstract])) OR (Genomics, Population[Title/Abstract])

#11 (((Imaging Genomics[MeSH Terms]) OR (Radiation Genomics[MeSH Terms])) OR (Radiation Oncology[MeSH Terms])) OR (Radiobiology[MeSH Terms])

#12 (((((((((((((((imaging radiogenomics[Title/Abstract])) OR (neuroimaging genomics[Title/Abstract])) OR (imaging genomics[Title/Abstract])) OR (Genomics, Imaging[Title/Abstract])) OR (Radiogenomic* [Title/Abstract])) OR (radiation genomics[Title/Abstract])) OR (radio-genomics[Title/Abstract])) OR (Oncology, Radiation[Title/Abstract])) OR (Therapeutic Radiology[Title/Abstract])) OR (Radiology, Therapeutic[Title/Abstract])) OR (radiation oncology[Title/Abstract])) OR (Biology, Radiation[Title/Abstract])) OR (Radiation Biology[Title/Abstract])) OR (radio biology[Title/Abstract])) OR (radiobiology'[Title/Abstract])

#13 #1 OR #2

#14 #3 OR #4

#15 #5 OR #6

#16 #7 OR #8

#17 #9 OR #10

#18 #11 OR #12

#19 #14 AND #15

#20 #19 OR #16

#21 #20 AND #17

#22 #21 OR #18

#23 #22 AND #13

**Table S1** The Newcastle-Ottawa Scale score of 11 included studies.

| The Newcastle-Ottawa Scale (NOS) | | | | | | | | | |
| --- | --- | --- | --- | --- | --- | --- | --- | --- | --- |
| Study id | Selection | | | | Comparability | Outcome | | | Total |
|  | Representativeness of the exposed cohort | Selection of the non exposed cohort | Ascertainment of exposure to implants | Demonstration that outcome of interest was not present at start of study | Comparability of cohorts on the basis of the design or analysis | Assessment of outcome | Was follow up long enough for outcomes to occur | Adequacy of follow up of cohorts |  |
| Shota Yamamoto, 2015 [25] | 1 | 1 | 1 | 1 | 2 | 0 | 1 | 1 | 8 |
| Jia Wu,2017 [26] | 1 | 1 | 1 | 1 | 2 | 1 | 0 | 1 | 8 |
| Ming Fan, 2019 [27] | 1 | 1 | 1 | 1 | 0 | 1 | 1 | 1 | 7 |
| Yunfang Yu, 2021[28] | 1 | 1 | 1 | 0 | 2 | 1 | 1 | 1 | 8 |
| Xuanyi Wang, 2022 [29] | 1 | 1 | 1 | 1 | 2 | 1 | 1 | 0 | 8 |
| Wenlong Ming, 2022 [30] | 1 | 1 | 1 | 1 | 1 | 1 | 0 | 0 | 6 |
| Yunfang YU,2023 [31] | 1 | 1 | 1 | 1 | 0 | 1 | 0 | 0 | 5 |
| Guan-Hua Su, 2023 [32] | 1 | 1 | 1 | 1 | 2 | 1 | 0 | 1 | 8 |
| Chao You, 2024 [33] | 1 | 1 | 1 | 1 | 2 | 1 | 1 | 1 | 9 |
| Xinyu Zhang, 2024 [34] | 1 | 1 | 1 | 1 | 2 | 1 | 1 | 1 | 8 |
| Ming Fan,2024 [35] | 1 | 1 | 1 | 1 | 1 | 1 | 1 | 0 | 7 |
| Mingping Hong, 2024 [36] | 1 | 1 | 1 | 0 | 2 | 1 | 1 | 1 | 8 |
| Xue Li, 2024 [37] | 1 | 1 | 1 | 0 | 0 | 0 | 1 | 1 | 5 |
| Wenci Liu, 2024 [38] | 1 | 1 | 1 | 0 | 0 | 1 | 1 | 1 | 6 |
| Ziyin Li，2024 [39] | 1 | 1 | 1 | 0 | 2 | 1 | 1 | 1 | 8 |
| Yu Hong Huang, 2025 [40] | 1 | 1 | 1 | 1 | 2 | 1 | 1 | 1 | 8 |

**Table S2** Details of Newcastle-Ottawa Scale scores with 16 included studies, reviewed by reader 1.

| The Newcastle-Ottawa Scale (NOS) | | | | | | | | | |
| --- | --- | --- | --- | --- | --- | --- | --- | --- | --- |
| Study id | Selection | | | | Comparability | Outcome | | | Total |
|  | Representativeness of the exposed cohort | Selection of the non exposed cohort | Ascertainment of exposure to implants | Demonstration that outcome of interest was not present at start of study | Comparability of cohorts on the basis of the design or analysis | Assessment of outcome | Was follow up long enough for outcomes to occur | Adequacy of follow up of cohorts |  |
| Shota Yamamoto, 2015 [25] | 1 | 1 | 1 | 1 | 2 | 0 | 1 | 1 | 8 |
| Jia Wu,2017 [26] | 1 | 1 | 1 | 1 | 0 | 1 | 0 | 1 | 6 |
| Ming Fan, 2019 [27] | 1 | 1 | 1 | 1 | 0 | 1 | 1 | 1 | 7 |
| Yunfang Yu, 2021[28] | 1 | 1 | 1 | 0 | 2 | 1 | 1 | 1 | 8 |
| Xuanyi Wang, 2022 [29] | 1 | 1 | 1 | 1 | 2 | 0 | 1 | 0 | 7 |
| Wenlong Ming, 2022 [30] | 1 | 1 | 1 | 1 | 2 | 0 | 0 | 0 | 6 |
| Yunfang YU,2023 [31] | 1 | 1 | 1 | 1 | 0 | 0 | 0 | 0 | 5 |
| Guan-Hua Su, 2023 [32] | 1 | 1 | 1 | 1 | 0 | 1 | 0 | 1 | 6 |
| Chao You, 2024 [33] | 1 | 1 | 1 | 1 | 2 | 1 | 1 | 1 | 9 |
| Xinyu Zhang, 2024 [34] | 1 | 1 | 1 | 1 | 2 | 1 | 0 | 1 | 8 |
| Ming Fan,2024 [35] | 1 | 1 | 1 | 1 | 2 | 1 | 0 | 0 | 7 |
| Mingping Hong, 2024 [36] | 1 | 1 | 1 | 0 | 2 | 1 | 1 | 1 | 8 |
| Xue Li, 2024 [37] | 1 | 1 | 1 | 0 | 0 | 0 | 1 | 1 | 5 |
| Wenci Liu, 2024 [38] | 1 | 1 | 1 | 0 | 0 | 1 | 1 | 1 | 6 |
| Ziyin Li，2024 [39] | 1 | 1 | 1 | 0 | 2 | 1 | 1 | 1 | 8 |
| Yu Hong Huang, 2025 [40] | 1 | 1 | 1 | 1 | 2 | 0 | 1 | 1 | 8 |

**Table S3** Details of Newcastle-Ottawa Scale scores with 16 included studies, reviewed by reader 2.

| The Newcastle-Ottawa Scale (NOS) | | | | | | | | | |
| --- | --- | --- | --- | --- | --- | --- | --- | --- | --- |
| Study id | Selection | | | | Comparability | Outcome | | | Total |
|  | Representativeness of the exposed cohort | Selection of the non exposed cohort | Ascertainment of exposure to implants | Demonstration that outcome of interest was not present at start of study | Comparability of cohorts on the basis of the design or analysis | Assessment of outcome | Was follow up long enough for outcomes to occur | Adequacy of follow up of cohorts |  |
| Shota Yamamoto, 2015 [25] | 1 | 1 | 1 | 1 | 2 | 0 | 1 | 1 | 8 |
| Jia Wu,2017 [26] | 1 | 1 | 1 | 1 | 0 | 1 | 0 | 1 | 8 |
| Ming Fan, 2019 [27] | 1 | 1 | 1 | 1 | 0 | 1 | 1 | 1 | 7 |
| Yunfang Yu, 2021[28] | 1 | 1 | 1 | 0 | 2 | 1 | 1 | 1 | 8 |
| Xuanyi Wang, 2022 [29] | 1 | 1 | 1 | 1 | 2 | 1 | 1 | 0 | 8 |
| Wenlong Ming, 2022 [30] | 1 | 1 | 1 | 1 | 1 | 1 | 0 | 0 | 6 |
| Yunfang YU,2023 [31] | 1 | 1 | 1 | 1 | 0 | 1 | 0 | 0 | 5 |
| Guan-Hua Su, 2023 [32] | 1 | 1 | 1 | 1 | 2 | 1 | 0 | 1 | 8 |
| Chao You, 2024 [33] | 1 | 1 | 1 | 1 | 2 | 1 | 1 | 1 | 9 |
| Xinyu Zhang, 2024 [34] | 1 | 1 | 1 | 1 | 2 | 1 | 1 | 1 | 8 |
| Ming Fan,2024 [35] | 1 | 1 | 1 | 1 | 1 | 1 | 1 | 0 | 7 |
| Mingping Hong, 2024 [36] | 1 | 1 | 1 | 0 | 2 | 1 | 1 | 1 | 8 |
| Xue Li, 2024 [37] | 1 | 1 | 1 | 0 | 0 | 0 | 1 | 1 | 5 |
| Wenci Liu, 2024 [38] | 1 | 1 | 1 | 0 | 0 | 1 | 1 | 1 | 6 |
| Ziyin Li，2024 [39] | 1 | 1 | 1 | 0 | 2 | 1 | 1 | 1 | 8 |
| Yu Hong Huang, 2025 [40] | 1 | 1 | 1 | 1 | 2 | 1 | 1 | 1 | 9 |

**Table S4** Radiomics quality scores for the included studies reviewed by reader 1.

| RQS number and name | Total sorce | Shota Yamamoto, 2015 [25] | Jia Wu,2017 [26] | Ming Fan,2019 [27] | Yunfang Yu, 2021 [28] | Xuanyi Wang,2022 [29] | Wenlong Ming,2022 [30] | Yunfang YU,2023 [31] | Guan-Hua Su,2023 [32] | Chao You,2024 [33] | Xinyu Zhang,2024 [34] | Ming Fan,2024 [35] | Mingping Hong, 2024 [36] | Xue Li, 2024 [37] | Wenci Liu,2024 [38] | Ziyin Li，2024 [39] | Yuhong Huang,2025 [40] |
| --- | --- | --- | --- | --- | --- | --- | --- | --- | --- | --- | --- | --- | --- | --- | --- | --- | --- |
| Image protocol quality | 2 | 1 | 1 | 1 | 1 | 0 | 1 | 1 | 1 | 1 | 1 | 1 | 1 | 1 | 1 | 1 | 1 |
| Imaging at multiple time points | 1 | 0 | 0 | 0 | 0 | 0 | 0 | 0 | 0 | 0 | 0 | 0 | 0 | 0 | 0 | 0 | 0 |
| Phantom study on all scanners | 1 | 0 | 0 | 0 | 0 | 0 | 0 | 0 | 0 | 0 | 0 | 0 | 0 | 0 | 0 | 0 | 0 |
| Multiple segmentations | 1 | 1 | 1 | 0 | 1 | 1 | 1 | 0 | 0 | 1 | 1 | 0 | 1 | 0 | 1 | 1 | 1 |
| Feature reduction | 3 or -3 | 3 | 3 | 3 | 3 | 3 | 3 | -3 | 3 | 3 | 3 | 3 | 3 | 3 | 3 | -3 | 3 |
| Multivariable analysis | 1 | 0 | 1 | 0 | 1 | 1 | 1 | 0 | 0 | 1 | 1 | 1 | 1 | 0 | 0 | 1 | 1 |
| biological correlates | 1 | 1 | 1 | 1 | 1 | 1 | 1 | 1 | 1 | 1 | 1 | 1 | 1 | 1 | 1 | 1 | 1 |
| Cut-off analyses | 1 | 1 | 1 | 1 | 1 | 1 | 0 | 1 | 0 | 1 | 1 | 1 | 1 | 0 | 1 | 1 | 0 |
| Discrimination statistics | 2 | 0 | 0 | 0 | 1 | 1 | 1 | 1 | 0 | 2 | 1 | 2 | 2 | 2 | 2 | 2 | 1 |
| Calibration statistics | 2 | 0 | 0 | 0 | 0 | 1 | 0 | 0 | 0 | 0 | 1 | 0 | 0 | 0 | 0 | 0 | 0 |
| Prospective study | 7 | 0 | 0 | 0 | 7 | 0 | 0 | 0 | 0 | 0 | 0 | 0 | 0 | 0 | 0 | 7 | 0 |
| Validation | 5 or -5 | 2 | 4 | 4 | 3 | 3 | 3 | 4 | 4 | 4 | 2 | 3 | 5 | 2 | 3 | 5 | 5 |
| Comparison to 'gold standard’ | 2 | 0 | 0 | 0 | 0 | 2 | 0 | 0 | 0 | 0 | 0 | 0 | 0 | 0 | 0 | 2 | 2 |
| Potential clinical utility | 2 | 0 | 0 | 0 | 2 | 2 | 0 | 0 | 0 | 0 | 2 | 0 | 0 | 2 | 2 | 0 | 2 |
| Cost-effectiveness analysis | 1 | 0 | 0 | 0 | 0 | 0 | 0 | 0 | 0 | 0 | 0 | 0 | 0 | 0 | 0 | 0 | 0 |
| Open data | 4 | 0 | 2 | 1 | 1 | 0 | 0 | 0 | 1 | 2 | 2 | 0 | 1 | 1 | 1 | 1 | 0 |
| % RQS (total score) | 100(36) | 25(9) | 39(14) | 31(11) | 67(24) | 44(16) | 31(11) | 14(5) | 28(10) | 44(16) | 44(16) | 33(12) | 44(16) | 33(12) | 39(15) | 53(19) | 47(17) |

**Table S5** Radiomics quality scores for the included studies reviewed by reader 2.

| RQS number and name | Total sorce | Shota Yamamoto, 2015 [25] | Jia Wu,2017 [26] | Ming Fan,2019 [27] | Yunfang Yu, 2021 [28] | Xuanyi Wang,2022 [29] | Wenlong Ming,2022 [30] | Yunfang YU,2023 [31] | Guan-Hua Su,2023 [32] | Chao You,2024 [33] | Xinyu Zhang,2024 [34] | Ming Fan,2024 [35] | Mingping Hong, 2024 [36] | Xue Li, 2024 [37] | Wenci Liu,2024 [38] | Ziyin Li，2024 [39] | Yuhong Huang,2025 [40] |
| --- | --- | --- | --- | --- | --- | --- | --- | --- | --- | --- | --- | --- | --- | --- | --- | --- | --- |
| Image protocol quality | 2 | 1 | 1 | 1 | 1 | 0 | 1 | 1 | 1 | 1 | 1 | 1 | 1 | 1 | 1 | 1 | 1 |
| Imaging at multiple time points | 1 | 0 | 0 | 0 | 0 | 0 | 0 | 0 | 0 | 0 | 0 | 0 | 0 | 0 | 0 | 0 | 0 |
| Phantom study on all scanners | 1 | 0 | 0 | 0 | 0 | 0 | 0 | 0 | 0 | 0 | 0 | 0 | 0 | 0 | 0 | 0 | 0 |
| Multiple segmentations | 1 | 1 | 1 | 0 | 1 | 1 | 1 | 0 | 0 | 1 | 1 | 0 | 1 | 0 | 1 | 1 | 1 |
| Feature reduction | 3 or -3 | 3 | 3 | 3 | 3 | 3 | 3 | -3 | 3 | 3 | 3 | 3 | 3 | 3 | 3 | -3 | 3 |
| Multivariable analysis | 1 | 0 | 0 | 0 | 1 | 1 | 0 | 0 | 1 | 1 | 0 | 1 | 1 | 0 | 1 | 1 | 0 |
| biological correlates | 1 | 1 | 1 | 1 | 1 | 1 | 1 | 1 | 1 | 1 | 1 | 1 | 1 | 1 | 1 | 1 | 1 |
| Cut-off analyses | 1 | 1 | 1 | 1 | 1 | 1 | 0 | 1 | 0 | 1 | 1 | 1 | 1 | 0 | 1 | 1 | 0 |
| Discrimination statistics | 2 | 0 | 0 | 0 | 1 | 2 | 1 | 1 | 0 | 2 | 1 | 2 | 1 | 1 | 1 | 2 | 1 |
| Calibration statistics | 2 | 0 | 0 | 0 | 0 | 1 | 0 | 0 | 0 | 0 | 1 | 0 | 0 | 0 | 0 | 0 | 0 |
| Prospective study | 7 | 0 | 0 | 0 | 0 | 0 | 0 | 0 | 0 | 0 | 0 | 0 | 0 | 0 | 0 | 0 | 0 |
| Validation | 5 or -5 | 2 | 4 | 4 | 4 | 3 | 3 | 4 | 4 | 4 | 2 | 3 | 5 | 2 | 3 | 5 | 5 |
| Comparison to 'gold standard’ | 2 | 0 | 0 | 0 | 0 | 2 | 0 | 0 | 2 | 0 | 0 | 2 | 2 | 0 | 0 | 2 | 2 |
| Potential clinical utility | 2 | 0 | 0 | 0 | 2 | 2 | 0 | 0 | 0 | 0 | 2 | 0 | 0 | 2 | 2 | 0 | 2 |
| Cost-effectiveness analysis | 1 | 0 | 0 | 0 | 0 | 0 | 0 | 0 | 0 | 0 | 0 | 0 | 0 | 0 | 0 | 0 | 0 |
| Open data | 4 | 0 | 1 | 1 | 1 | 1 | 0 | 0 | 1 | 2 | 1 | 0 | 1 | 1 | 1 | 1 | 0 |
| % RQS (total score) | 100(36) | 25(9) | 33(12) | 31(11) | 50(18) | 50(18) | 28(10) | 14(5) | 36(13) | 44(16) | 39(14) | 39(14) | 47(17) | 31(11) | 39(15) | 42(15) | 44(16) |

**Table S6** Quality assessment of prognostic accuracy studies assessment results of included studies by reader 1.

| **Study ID** | **Risk of Bias** | | | | | **Applicability Concern** | | | | **Overall** |
| --- | --- | --- | --- | --- | --- | --- | --- | --- | --- | --- |
|  | **Participants** | **Index Test** | **Outcome** | **Flow and Timing** | **Analysis** | **Participants** | **Index Test** | **Outcome** | **Flow and Timing** |  |
| Shota Yamamoto, 2015 [25] | Low | High | Low | High | High | High | Low | Low | Low | High |
| Jia Wu, 2017 [26] | Low | High | Unclear | High | High | Low | Low | Low | High | High |
| Ming Fan, 2019 [27] | Low | High | Low | High | High | Low | Low | Low | Low | High |
| Yunfang Yu, 2021 [28] | Low | High | Low | High | High | Low | Low | Low | Unclear | High |
| Xuanyi Wang, 2022 [29] | Low | High | Low | High | Unclear | High | Low | Low | Low | High |
| Wenlong Ming, 2022 [30] | Low | Unclear | Low | High | High | Low | Low | Low | Unclear | High |
| Yunfang YU, 2023 [31] | Low | High | Unclear | High | Unclear | Low | Low | Low | High | High |
| Guan-Hua Su, 2023 [32] | Low | High | Low | High | High | Low | Low | Low | High | High |
| Chao You,2024 [33] | Low | High | Low | High | High | Low | Low | Low | Low | High |
| Xinyu Zhang, 2024 [34] | Low | High | Low | High | High | High | Low | Low | Low | High |
| Ming Fan,2024 [35] | Low | High | Low | High | High | Low | Low | Low | Unclear | High |
| Mingping Hong, 2024 [36] | Low | High | Unclear | High | High | Low | Low | Low | Low | High |
| Xue Li, 2024 [37] | Low | High | Low | High | High | Low | Low | Low | Low | High |
| Wenci Liu,2024 [38] | Low | High | Low | High | High | Low | Low | Low | Low | High |
| Ziyin Li，2024 [39] | Low | High | Unclear | High | High | Low | Low | Low | Low | High |
| Yuhong Huang,2025 [40] | Low | Unclear | Low | High | High | Low | Low | Low | Unclear | High |

**Table S7** Quality assessment of prognostic accuracy studies assessment results of included studies by reader 2.

| **Study ID** | **Risk of Bias** | | | | | **Applicability Concern** | | | | **Overall** |
| --- | --- | --- | --- | --- | --- | --- | --- | --- | --- | --- |
|  | **Participants** | **Index Test** | **Outcome** | **Flow and Timing** | **Analysis** | **Participants** | **Index Test** | **Outcome** | **Flow and Timing** |  |
| Shota Yamamoto, 2015 [25] | Low | High | Low | High | High | High | Low | Low | Low | High |
| Jia Wu, 2017 [26] | Low | High | Low | High | High | Low | Low | Low | High | High |
| Ming Fan, 2019 [27] | Low | High | Low | High | High | Low | Low | Low | Low | High |
| Yunfang Yu, 2021 [28] | Low | High | Low | High | Unclear | Low | Low | Low | Low | High |
| Xuanyi Wang, 2022 [29] | Low | High | Low | High | High | High | Low | Low | Low | High |
| Wenlong Ming, 2022 [30] | Low | Unclear | Low | Unclear | High | Low | Low | Low | Unclear | High |
| Yunfang YU, 2023 [31] | Low | High | Low | High | High | Low | Low | Low | High | High |
| Guan-Hua Su, 2023 [32] | Low | High | Low | High | High | Low | Low | Low | High | High |
| Chao You,2024 [33] | Low | High | Low | High | High | Low | Low | Low | Low | High |
| Xinyu Zhang, 2024 [34] | Low | High | Low | High | High | High | Low | Low | Low | High |
| Ming Fan,2024 [35] | Unlear | High | Low | High | High | Low | Low | Low | Unclear | High |
| Mingping Hong, 2024 [36] | Low | Unclear | Low | High | High | Low | Low | Low | High | High |
| Xue Li, 2024 [37] | Unclear | High | Low | High | High | Low | Low | Low | Low | High |
| Wenci Liu,2024 [38] | Unclear | High | Low | High | High | Low | Low | Low | Low | High |
| Ziyin Li，2024 [39] | Low | High | Low | Unclear | High | Low | Low | Low | Unclear | High |
| Yuhong Huang,2025 [40] | Low | High | Low | High | High | Low | Low | Low | Unclear | High |

**Table S8** The ICC values for the NOS assessment

| Intraclass Correlation Coefficient | | | | | | | |
| --- | --- | --- | --- | --- | --- | --- | --- |
|  | Intraclass Correlation^b^ | 95% Confidence Interval | | F Test with True Value 0 | | | |
|  |  | Lower Bound | Upper Bound | Value | df1 | df2 | Sig |
| Single Measures | .780^a^ | .478 | .917 | 8.100 | 15 | 15 | .000 |
| Average Measures | .877 | .647 | .957 | 8.100 | 15 | 15 | .000 |
| Two-way random effects model where both people effects and measures effects are random. | | | | | | | |
| a. The estimator is the same, whether the interaction effect is present or not. | | | | | | | |
| b. Type C intraclass correlation coefficients using a consistency definition. The between-measure variance is excluded from the denominator variance. | | | | | | | |

**Table S9** The ICC values for the RQS assessment

| Intraclass Correlation Coefficient | | | | | | | |
| --- | --- | --- | --- | --- | --- | --- | --- |
|  | Intraclass Correlation^b^ | 95% Confidence Interval | | F Test with True Value 0 | | | |
|  |  | Lower Bound | Upper Bound | Value | df1 | df2 | Sig |
| Single Measures | .845^a^ | .620 | .942 | 11.871 | 15 | 15 | .000 |
| Average Measures | .916 | .765 | .970 | 11.871 | 15 | 15 | .000 |
| Two-way random effects model where both people effects and measures effects are random. | | | | | | | |
| a. The estimator is the same, whether the interaction effect is present or not. | | | | | | | |
| b. Type A intraclass correlation coefficients using an absolute agreement definition. | | | | | | | |

**Table S10** The Kappa values for the QUAPAS assessment

| Study ID | Number of items with consistent QUAPAS | Kappa value |
| --- | --- | --- |
| Shota Yamamoto, 2015 [25] | 10 | 1 |
| Jia Wu, 2017 [26] | 9 | 0.818 |
| Ming Fan,2019 [27] | 10 | 1 |
| Yunfang Yu，2021[28] | 8 | 0.667 |
| Xuanyi Wang,2022 [29] | 9 | 0.818 |
| Wenlong Ming,2022 [30] | 10 | 1 |
| Yunfang YU,2023 [31] | 8 | 0.667 |
| Guan-Hua Su, 2023 [32] | 10 | 1 |
| Chao You,2024 [33] | 10 | 1 |
| Xinyu Zhang,2024 [34] | 9 | 0.818 |
| Ming Fan,2024 [35] | 9 | 0.818 |
| Mingping Hong, 2024[36] | 8 | 0.667 |
| Xue Li, 2024[37] | 9 | 0.818 |
| Wenci Liu,2024[38] | 9 | 0.818 |
| Ziyin Li, 2024[39] | 7 | 0.508 |
| Yuhong Huang, 2025 [40] | 8 | 0.667 |
| Average | 8.94 | 0.818 |
